# Supplementary material for: Chemical Constituents of the Deep-Sea-Derived Penicillium solitum
Source: Mar Drugs. 2021 Oct 17;19(10):580. doi: 10.3390/md19100580 (PMC8540044; doi:10.3390/md19100580)
Supplement: Supplementary file 1 [file marinedrugs-19-00580-s001.zip › marinedrugs-1416452-SI.pdf]

Supporting Information

Chemical Constituents of the Deep-Sea-Derived *Penicillium solitum*

Zhi-Hui He [1,†], Jia Wu [2,†], Lin Xu [1], Man-Yi Hu [1], Ming-Ming Xie [1], You-Jia Hao [1], Shu-Jin Li [1], Zongze Shao [1] and Xian-Wen Yang [1,*]

[1] Key Laboratory of Marine Biogenetic Resources, Third Institute of Oceanography, Ministry of Natural Resources, 184 Daxue Road, Xiamen 361005, China; hezhihui@tio.org.cn (Z.-H.H.); xulin@tio.org.cn (L.X.); humanyi@tio.org.cn (M.-Y.H.); xiemingmin@tio.org.cn (M.-M.X.); haoyoujia888@163.com (Y.-J.H.), lishujin98@163.com (S.-J.L.); shaozongze@tio.org.cn (Z.S.)

[2] Yanjing Medical College, Capital Medical University, 4 Dadong Road, Beijing 101300, China; wujia@ccmu.edu.cn

* Correspondence: yangxianwen@tio.org.cn; +86-592-219-5319

† These authors contributed equally to this work.

Content

1. ECD Calculation

Table S1. Energies analysis for 1*S*,2*S*,8*S*,8a*R*,11*R*,13*R*-**1** at MMFF94 force field.

Table S2. Standard orientations at BP86/6-311G(d,p) level in MeOH.

2. Legends of Figures

Figure S1. ^{1}H NMR spectrum of **1** in MeOH-d_4.

Figure S2. ^{13}C NMR spectrum of **1** in MeOH-d_4.

Figure S3. HSQC NMR spectrum of **1** in MeOH-d_4.

Figure S4. COSY NMR spectrum of **1** in MeOH-d_4.

Figure S5. HMBC NMR spectrum of **1** in MeOH-d_4.

Figure S6. NOESY NMR spectrum of **1** in MeOH-d_4.

Figure S7. The HR-ESI-MS spectrum of **1**.

Figure S8. ^{1}H NMR spectrum of **2** in DMSO-d_6.

Figure S9. ^{13}C NMR spectrum of **2** in DMSO-d_6.

Figure S10. HSQC NMR spectrum of **2** in DMSO-d_6.

Figure S11. COSY NMR spectrum of **2** in DMSO-d_6.

Figure S12. HMBC NMR spectrum of **2** in DMSO-d_6.

Figure S13. NOESY NMR spectrum of **2** in DMSO-d_6.

Figure S14. The HR-ESI-MS spectrum of **2**.

Figure S15. ^{1}H NMR spectrum of **3** in MeOH-d_4.

Figure S16. ^{13}C NMR spectrum of **3** in MeOH-d_4.

Figure S17. HSQC NMR spectrum of **3** in MeOH-d_4.

Figure S18. COSY NMR spectrum of **3** in MeOH-d_4.

Figure S19. HMBC NMR spectrum of **3** in MeOH-d_4.

Figure S20. NOESY NMR spectrum of **3** in MeOH-d_4.

Figure S21. The HR-ESI-MS spectrum of **3**.

Figure S22. ^{1}H NMR spectrum of **4**.

Figure S23. ^{13}C NMR spectrum of **4**.
Figure S24. ^{1}H NMR spectrum of **5**.
Figure S25. ^{13}C NMR spectrum of **5**.
Figure S26. ^{1}H NMR spectrum of **6**.
Figure S27. ^{13}C NMR spectrum of **6**.
Figure S28. ^{1}H NMR spectrum of **7**.
Figure S29. ^{13}C NMR spectrum of **7**.
Figure S30. ^{1}H NMR spectrum of **8**.
Figure S31. ^{13}C NMR spectrum of **8**.
Figure S32. ^{1}H NMR spectrum of **9**.
Figure S33. ^{13}C NMR spectrum of **9**.
Figure S34. ^{1}H NMR spectrum of **10**.
Figure S35. ^{13}C NMR spectrum of **10**.
Figure S36. ^{1}H NMR spectrum of **11**.
Figure S37. ^{13}C NMR spectrum of **11**.
Figure S38. ^{1}H NMR spectrum of **12**.
Figure S39. ^{13}C NMR spectrum of **12**.
Figure S40. ^{1}H NMR spectrum of **13**.
Figure S41. ^{13}C NMR spectrum of **13**.
Figure S42. ^{1}H NMR spectrum of **14**.
Figure S43. ^{13}C NMR spectrum of **14**.
Figure S44. ^{1}H NMR spectrum of **15**.
Figure S45. ^{13}C NMR spectrum of **15**.
Figure S46. ^{1}H NMR spectrum of **16**.
Figure S47. ^{13}C NMR spectrum of **16**.
Figure S48. ^{1}H NMR spectrum of **17**.
Figure S49. ^{13}C NMR spectrum of **17**.
Figure S50. ^{1}H NMR spectrum of **18**.
Figure S51. ^{13}C NMR spectrum of **18**.
Figure S52. ^{1}H NMR spectrum of **19**.
Figure S53. ^{13}C NMR spectrum of **19**.
Figure S54. ^{1}H NMR spectrum of **20**.
Figure S55. ^{13}C NMR spectrum of **20**.
Figure S56. ^{1}H NMR spectrum of **21**.
Figure S57. ^{13}C NMR spectrum of **21**.
Figure S58. ^{1}H NMR spectrum of **22**.
Figure S59. ^{13}C NMR spectrum of **22**.
Figure S60. ^{1}H NMR spectrum of **23**.
Figure S61. ^{13}C NMR spectrum of **23**.
Figure S62. ^{1}H NMR spectrum of **24**.
Figure S63. ^{13}C NMR spectrum of **24**.
Figure S64. ^{1}H NMR spectrum of **25**.
Figure S65. ^{13}C NMR spectrum of **25**.

Table S1. Energies analysis for 1*S*,2*S*,8*S*,8a*R*,11*R*,13*R*-**1** at MMFF94 force field.

Configuration	Conformer	Structure	E (kcal/mol)	Population (%)
1*S*,2*S*,8*S*,8a*R*,11*R*,13*R*-**1**	1	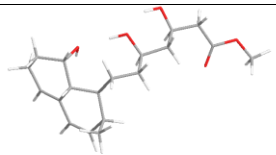	26.35	97.09
1*S*,2*S*,8*S*,8a*R*,11*R*,13*R*-**1**	2	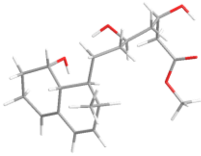	25.46	1.69
1*S*,2*S*,8*S*,8a*R*,11*R*,13*R*-**1**	3	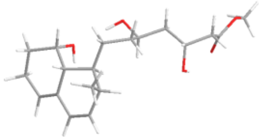	26.38	1.22

Table S2. Standard orientations at BP86/6-311G(d,p) level in MeOH..

Conformer 1*S*,2*S*,8*S*,8a*R*,11*R*,13*R*-**1**-1

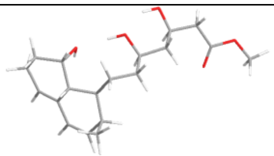

SCF Energy (BP86): −1118.500294 Hartree: −701869.5253 kcal/mol

Center Number	Atomic Number	Atomic Type	Coordinates (Angstroms) X	Y	Z
1	6	0	2.867822	3.147789	1.468235
2	6	0	2.154288	2.410052	0.312449
3	6	0	2.866963	2.688329	-0.988051
4	6	0	3.627330	1.781452	-1.621223
5	6	0	3.866983	0.435824	-1.102085
6	6	0	4.538898	-0.476190	-1.835442
7	6	0	4.835799	-1.883640	-1.395952
8	6	0	4.517470	-2.101492	0.090216
9	6	0	3.218984	-1.399546	0.500503
10	8	0	2.087476	-1.959657	-0.199993
11	6	0	3.319407	0.126992	0.293194
12	6	0	1.973675	0.877208	0.507142
13	6	0	1.242435	0.511979	1.818578
14	6	0	-0.289366	0.651626	1.756250
15	6	0	-0.970086	-0.337580	0.789780
16	6	0	-2.493979	-0.318807	0.935028
17	6	0	-3.215076	-1.244406	-0.058615
18	8	0	-2.964162	-2.614537	0.223438

19	6	0	-4.736768	-1.086073	-0.020417
20	6	0	-5.245648	0.213915	-0.601914
21	8	0	-4.570584	1.090367	-1.102697
22	8	0	-6.593754	0.288817	-0.503588
23	6	0	-7.185701	1.481575	-1.040589
24	8	0	-0.518550	-1.682859	1.023052
25	1	0	3.856446	2.720373	1.670365
26	1	0	2.285252	3.121739	2.394805
27	1	0	3.017402	4.201395	1.205739
28	1	0	1.147509	2.847274	0.235345
29	1	0	2.755253	3.685726	-1.411050
30	1	0	4.103981	2.035279	-2.567089
31	1	0	4.889192	-0.187847	-2.826857
32	1	0	4.260788	-2.592569	-2.013264
33	1	0	5.888437	-2.125783	-1.595819
34	1	0	4.441152	-3.170584	0.316177
35	1	0	5.330981	-1.695375	0.705607
36	1	0	3.018104	-1.621673	1.550683
37	1	0	2.139446	-1.658812	-1.122930
38	1	0	4.045536	0.495865	1.034615
39	1	0	1.332684	0.560686	-0.325590
40	1	0	1.627472	1.114780	2.648669
41	1	0	1.445042	-0.525530	2.095007
42	1	0	-0.582526	1.672375	1.473865
43	1	0	-0.691763	0.475800	2.762573
44	1	0	-0.730462	-0.056215	-0.248249
45	1	0	-2.761386	-0.623826	1.956497
46	1	0	-2.839091	0.710290	0.785806
47	1	0	-2.871775	-0.988277	-1.076301
48	1	0	-2.020015	-2.657169	0.476152
49	1	0	-5.186848	-1.914123	-0.582085
50	1	0	-5.109127	-1.189744	1.006527
51	1	0	-6.970952	1.572121	-2.108989
52	1	0	-8.258352	1.376731	-0.873914
53	1	0	-6.802461	2.367885	-0.527429
54	1	0	0.356085	-1.801874	0.596916

Conformer 1*S*,2*S*,8*S*,8a*R*,11*R*,13*R*-**1**-2

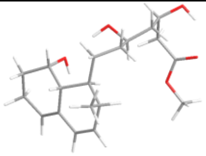

SCF Energy (BP86): −1118.496467 Hartree: −701867.1243 kcal/mol

Center Number	Atomic Number	Atomic Type	Coordinates (Angstroms) X	Y	Z
1	6	0	2.669851	1.432245	3.067910
2	6	0	1.822800	1.519218	1.777791
3	6	0	2.409206	2.554101	0.849211
4	6	0	3.068033	2.245260	-0.278862
5	6	0	3.311842	0.869909	-0.711483
6	6	0	3.864952	0.613157	-1.915196
7	6	0	4.154671	-0.762085	-2.451145
8	6	0	4.004478	-1.842918	-1.371362
9	6	0	2.785113	-1.587400	-0.479260

10	8	0	1.562491	-1.667090	-1.240491
11	6	0	2.909174	-0.238715	0.262806
12	6	0	1.616508	0.175593	1.021747
13	6	0	1.015388	-0.936870	1.908211
14	6	0	-0.503401	-0.827429	2.131971
15	6	0	-1.342711	-1.031201	0.851700
16	6	0	-2.836216	-1.124517	1.180975
17	6	0	-3.747601	-1.488228	-0.005631
18	8	0	-5.109599	-1.532637	0.401211
19	6	0	-3.576023	-0.575026	-1.239501
20	6	0	-3.898695	0.871330	-0.942833
21	8	0	-4.778809	1.247342	-0.186192
22	8	0	-3.106835	1.726199	-1.620175
23	6	0	-3.387661	3.123748	-1.422573
24	8	0	-0.976859	-2.221960	0.158758
25	1	0	3.659815	1.005065	2.872239
26	1	0	2.180961	0.829431	3.839811
27	1	0	2.825226	2.434239	3.484267
28	1	0	0.827144	1.876261	2.081404
29	1	0	2.292444	3.598961	1.134211
30	1	0	3.454868	3.037637	-0.918384
31	1	0	4.114883	1.455022	-2.561461
32	1	0	3.482996	-0.978387	-3.297571
33	1	0	5.165929	-0.793458	-2.878909
34	1	0	3.920805	-2.835236	-1.827607
35	1	0	4.897038	-1.851737	-0.731969
36	1	0	2.703646	-2.403018	0.242458
37	1	0	1.517757	-0.873196	-1.799412
38	1	0	3.721304	-0.359126	0.997026
39	1	0	0.883002	0.401182	0.237198
40	1	0	1.524827	-0.966812	2.878225
41	1	0	1.188338	-1.917497	1.458313
42	1	0	-0.768872	0.141635	2.578146
43	1	0	-0.798671	-1.597678	2.856313
44	1	0	-1.191478	-0.160487	0.190690
45	1	0	-2.993890	-1.890433	1.950048
46	1	0	-3.165246	-0.169775	1.613146
47	1	0	-3.496256	-2.502137	-0.326020
48	1	0	-5.374797	-0.604562	0.537728
49	1	0	-2.572751	-0.660197	-1.663509
50	1	0	-4.287042	-0.913848	-2.004460
51	1	0	-3.262454	3.395262	-0.371224
52	1	0	-2.668471	3.656026	-2.045502
53	1	0	-4.410540	3.356284	-1.729796
54	1	0	-0.131881	-2.065296	-0.307160

Conformer 1*S*,2*S*,8*S*,8a*R*,11*R*,13*R*-**1**-3

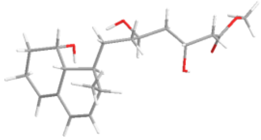

SCF Energy (BP86): −1118.496162 Hartree: −701866.9326 kcal/mol

Center	Atomic	Atomic	Coordinates (Angstroms)		
Number	Number	Type	X	Y	Z

1	6	0	-2.760521	3.187630	-0.895459
2	6	0	-2.135658	2.216564	0.131906
3	6	0	-2.919893	2.261119	1.420076
4	6	0	-3.730086	1.271817	1.827684
5	6	0	-3.959709	0.055129	1.050474
6	6	0	-4.684279	-0.962555	1.560725
7	6	0	-4.973335	-2.254900	0.847848
8	6	0	-4.572201	-2.197670	-0.633142
9	6	0	-3.244357	-1.459881	-0.834333
10	8	0	-2.158889	-2.162583	-0.196885
11	6	0	-3.340756	0.002786	-0.347926
12	6	0	-1.974974	0.747136	-0.351348
13	6	0	-1.197796	0.623840	-1.680020
14	6	0	0.325056	0.798587	-1.546013
15	6	0	1.003282	-0.321060	-0.726669
16	6	0	2.532452	-0.288964	-0.832793
17	6	0	3.183897	1.002108	-0.313639
18	8	0	2.778499	1.322748	1.012258
19	6	0	4.724264	0.965137	-0.422555
20	6	0	5.354853	-0.081062	0.472290
21	8	0	4.898025	-0.454369	1.540272
22	8	0	6.511612	-0.546575	-0.029460
23	6	0	7.200978	-1.514468	0.783933
24	8	0	0.607244	-1.615183	-1.177730
25	1	0	-3.741637	2.837701	-1.236268
26	1	0	-2.119808	3.323601	-1.772749
27	1	0	-2.907355	4.174249	-0.440826
28	1	0	-1.126907	2.596552	0.350695
29	1	0	-2.815892	3.156508	2.031517
30	1	0	-4.256534	1.354657	2.777725
31	1	0	-5.085218	-0.858710	2.569428
32	1	0	-4.443032	-3.079920	1.350272
33	1	0	-6.038773	-2.506064	0.941603
34	1	0	-4.495380	-3.207392	-1.051082
35	1	0	-5.345019	-1.666201	-1.204141
36	1	0	-2.990746	-1.483102	-1.896337
37	1	0	-2.254230	-2.031470	0.761593
38	1	0	-4.021283	0.521175	-1.041947
39	1	0	-1.376254	0.256117	0.426086
40	1	0	-1.586689	1.339747	-2.413712
41	1	0	-1.354161	-0.363357	-2.120786
42	1	0	0.564580	1.770447	-1.094966
43	1	0	0.759689	0.798776	-2.555626
44	1	0	0.745661	-0.193165	0.334625
45	1	0	2.917140	-1.150284	-0.272634
46	1	0	2.813136	-0.442776	-1.884555
47	1	0	2.845557	1.844297	-0.927668
48	1	0	3.215417	0.664511	1.585351
49	1	0	5.051696	0.806247	-1.454630
50	1	0	5.118447	1.938780	-0.101750
51	1	0	6.579871	-2.401827	0.929130
52	1	0	8.106705	-1.764781	0.231545
53	1	0	7.448335	-1.088124	1.759439
54	1	0	-0.286544	-1.806261	-0.833217

Figure S1. 1H NMR spectrum of 1 in MeOH-d4.

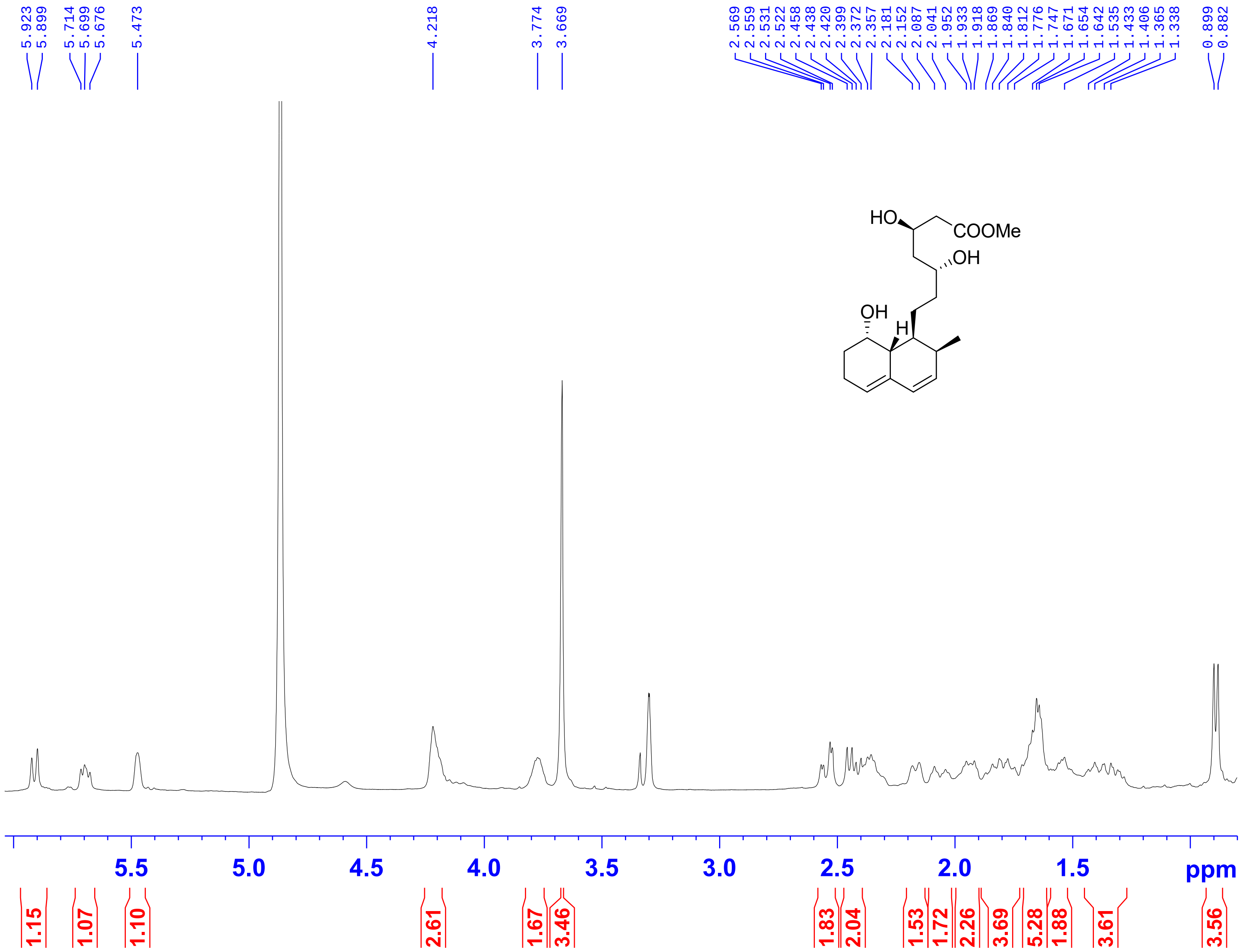

BRUKER

Current Data Parameters
NAME HB2-10 M 10mg
EXPNO 1
PROCNO 1

F2 - Acquisition Parameters
Date_ 20200801
Time 13.35
INSTRUM spect
PROBHD 5 mm PABBO BB-
PULPROG zg30
TD 65536
SOLVENT MeOD
NS 19
DS 2
SWH 8012.820 Hz
FIDRES 0.122266 Hz
AQ 4.0894465 sec
RG 128
DW 62.400 usec
DE 6.50 usec
TE 296.5 K
D1 1.00000000 sec
TD0 1

======== CHANNEL f1 ========
SFO1 400.1324710 MHz
NUC1 1H
P1 13.90 usec
PLW1 12.14299965 W

F2 - Processing parameters
SI 32768
SF 400.1300116 MHz
WDW EM
SSB 0
LB 0.30 Hz
GB 0
PC 1.00

Figure S2. 13C NMR spectrum of 1 in MeOH-d4.

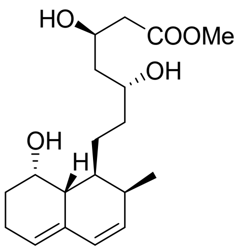

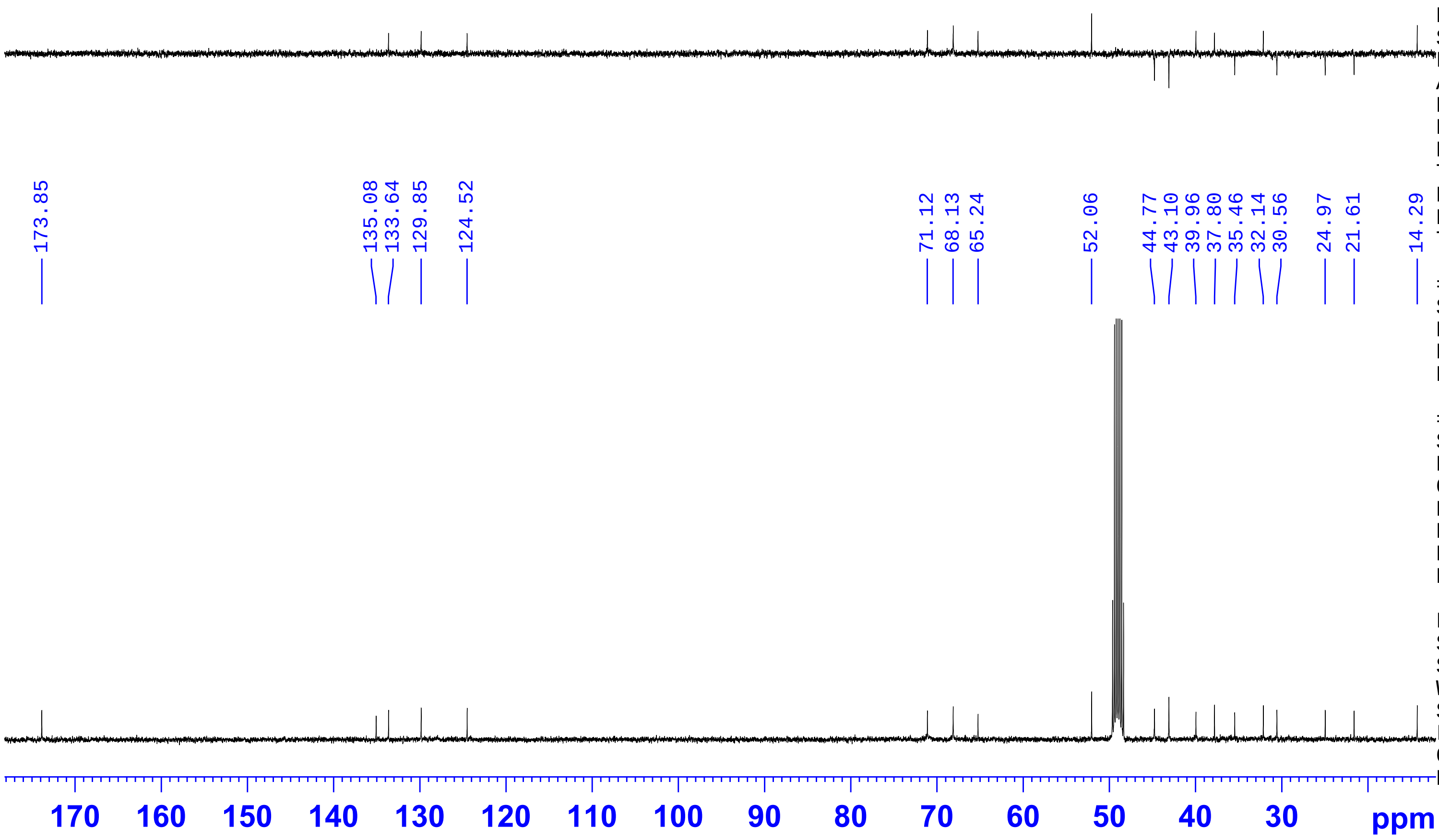

Current Data Parameters
NAME HB2-10 M 10mg
EXPNO 2
PROCNO 1

F2 - Acquisition Parameters
Date_ 20200801
Time 13.38
INSTRUM spect
PROBHD 5 mm PABBO BB-
PULPROG zgpg30
TD 65536
SOLVENT MeOD
NS 482
DS 4
SWH 24038.461 Hz
FIDRES 0.366798 Hz
AQ 1.3631488 sec
RG 203
DW 20.800 usec
DE 6.50 usec
TE 296.6 K
D1 2.00000000 sec
D11 0.03000000 sec
TD0 1

======== CHANNEL f1 ========
SFO1 100.6228293 MHz
NUC1 13C
P1 12.37 usec
PLW1 28.13500023 W

======== CHANNEL f2 ========
SFO2 400.1316005 MHz
NUC2 1H
CPDPRG[2 waltz16
PCPD2 90.00 usec
PLW2 12.14299965 W
PLW12 0.28964999 W
PLW13 0.23461001 W

F2 - Processing parameters
SI 32768
SF 100.6126294 MHz
WDW EM
SSB 0
LB 1.00 Hz
GB 0
PC 1.40

Figure S3. HSQC NMR spectrum of 1 in MeOH-d4.

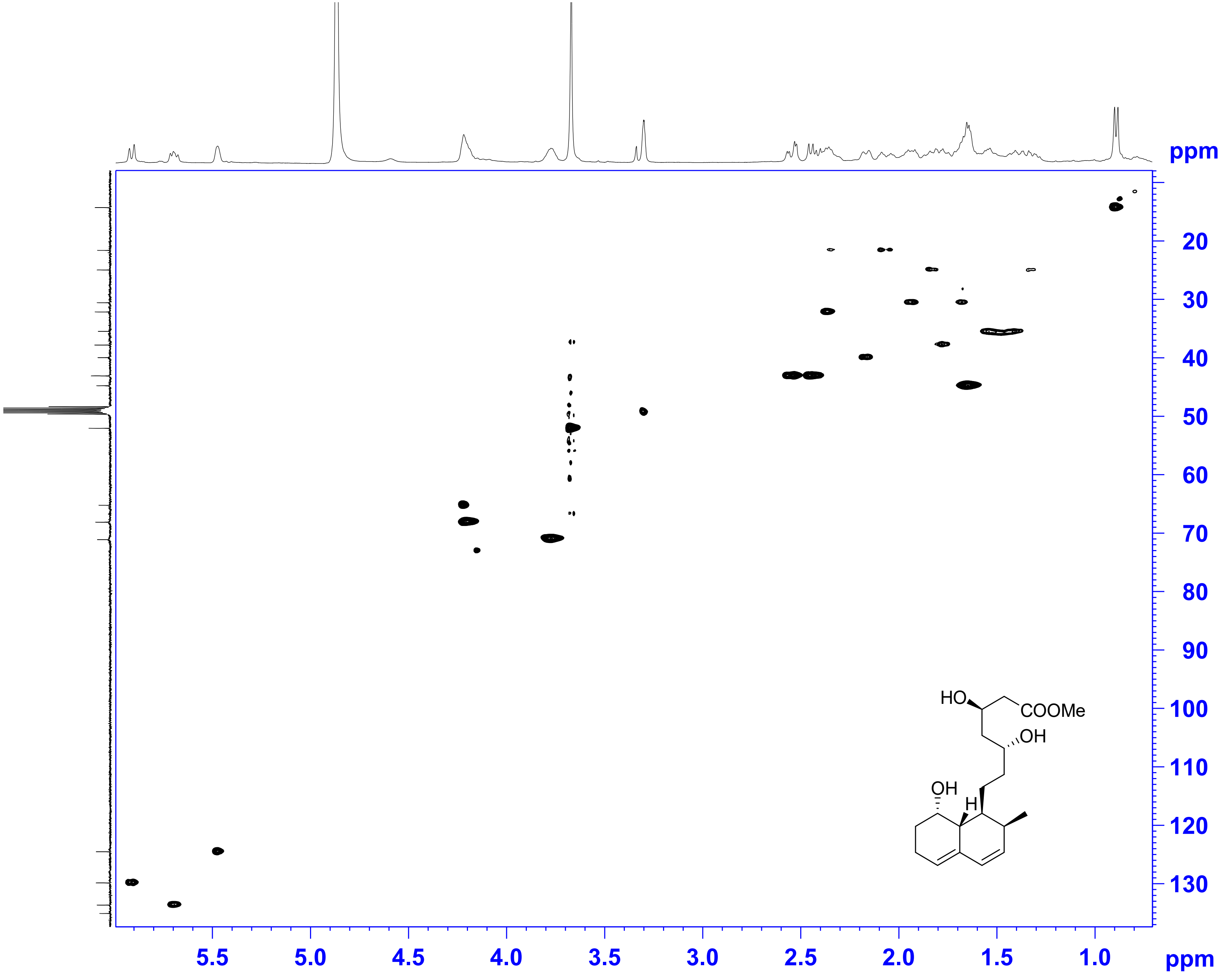


```
Current Data Parameters
NAME       HB2-10 M 10mg
EXPNO                  4
PROCNO                 1

F2 - Acquisition Parameters
Date_           20200813
Time               21.17
INSTRUM            spect
PROBHD   5 mm PABBO BB-
PULPROG  hsqcetgpsisp2.2
TD                  1024
SOLVENT             MeOD
NS                    16
DS                    32
SWH             3201.024 Hz
FIDRES          3.126000 Hz
AQ             0.1599488 sec
RG                   203
DW               156.200 usec
DE                  6.50 usec
TE                 296.1 K
CNST2        145.0000000
CNST17        -0.5000000
D0            0.00000300 sec
D1            2.00000000 sec
D4            0.00172414 sec
D11           0.03000000 sec
D16           0.00020000 sec
D24           0.00089000 sec
IN0           0.00002480 sec

======== CHANNEL f1 ========
SFO1         400.1316005 MHz
NUC1                  1H
P1                 13.90 usec
P2                 27.80 usec
P28              1000.00 usec
PLW1         12.14299965 W

======== CHANNEL f2 ========
SFO2         100.6228298 MHz
NUC2                 13C
CPDPRG[2            garp
P3                 12.37 usec
P14               500.00 usec
P24              2000.00 usec
PCPD2              70.00 usec
PLW0     0 W
PLW2         28.13500023 W
PLW12         0.87860000 W
SPNAM[3] Crp60,0.5,20.1
SPOAL3             0.500
SPOFFS3  0 Hz
SPW3          6.57779980 W
SPNAM[7]     Crp60comp.4
SPOAL7             0.500
SPOFFS7  0 Hz
SPW7          6.57779980 W

====== GRADIENT CHANNEL =====
GPNAM[1]      SMSQ10.100
GPNAM[2]      SMSQ10.100
GPNAM[3]      SMSQ10.100
GPNAM[4]      SMSQ10.100
GPZ1               80.00 %
GPZ2               20.10 %
GPZ3               11.00 %
GPZ4               -5.00 %
P16              1000.00 usec
P19               600.00 usec

F1 - Acquisition parameters
TD                   256
SFO1            100.6228 MHz
FIDRES         78.755043 Hz
SW               200.365 ppm
FnMODE      Echo-Antiecho

F2 - Processing parameters
SI                  1024
SF           400.1300075 MHz
WDW                QSINE
SSB                    2
LB       0 Hz
GB       0
PC                  1.40

F1 - Processing parameters
SI                  1024
MC2         echo-antiecho
SF           100.6126294 MHz
WDW                QSINE
SSB                    2
LB       0 Hz
GB       0
```

Figure S4. COSY NMR spectrum of 1 in MeOH-d4.

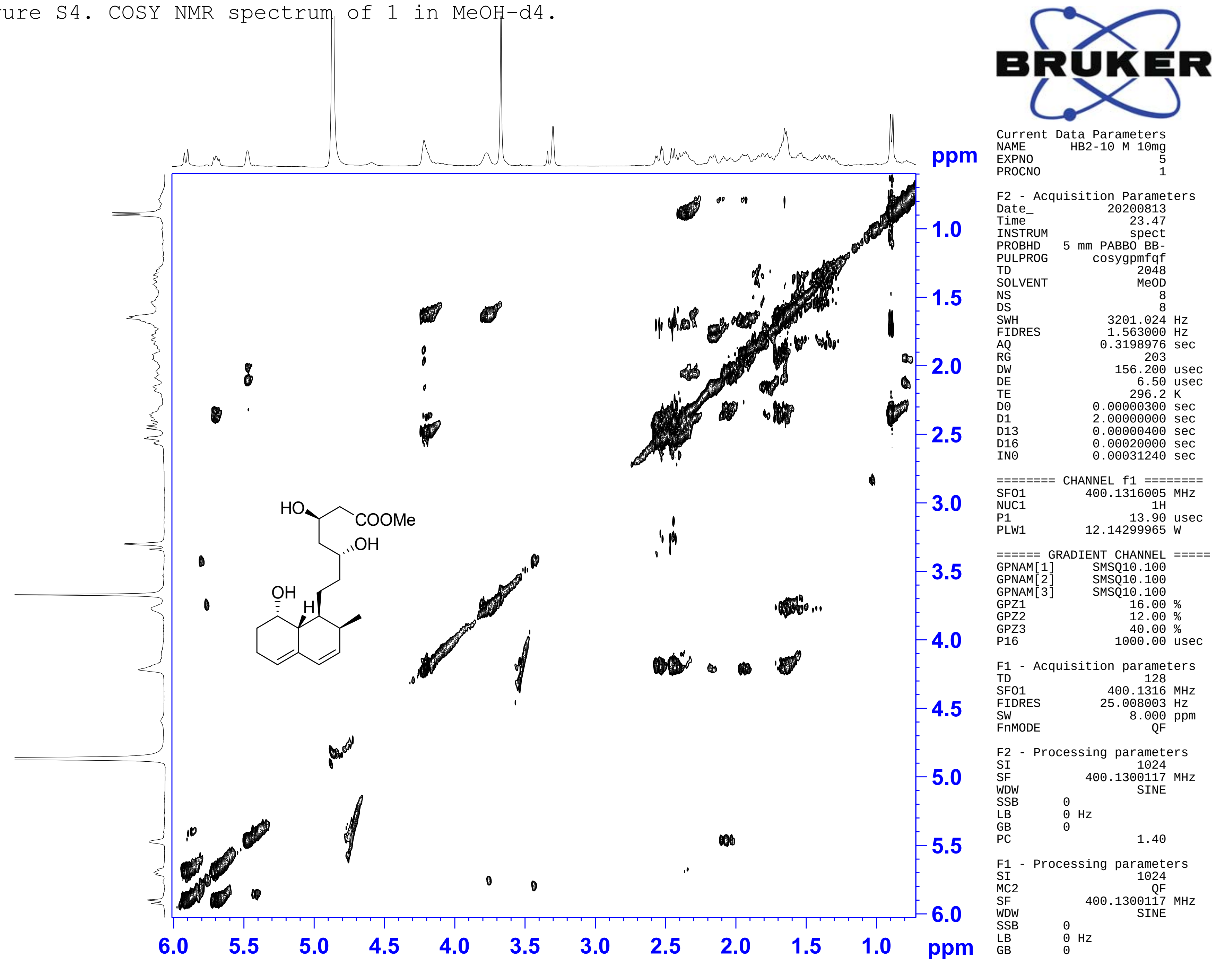

Figure S5. HMBC NMR spectrum of 1 in MeOH-d4

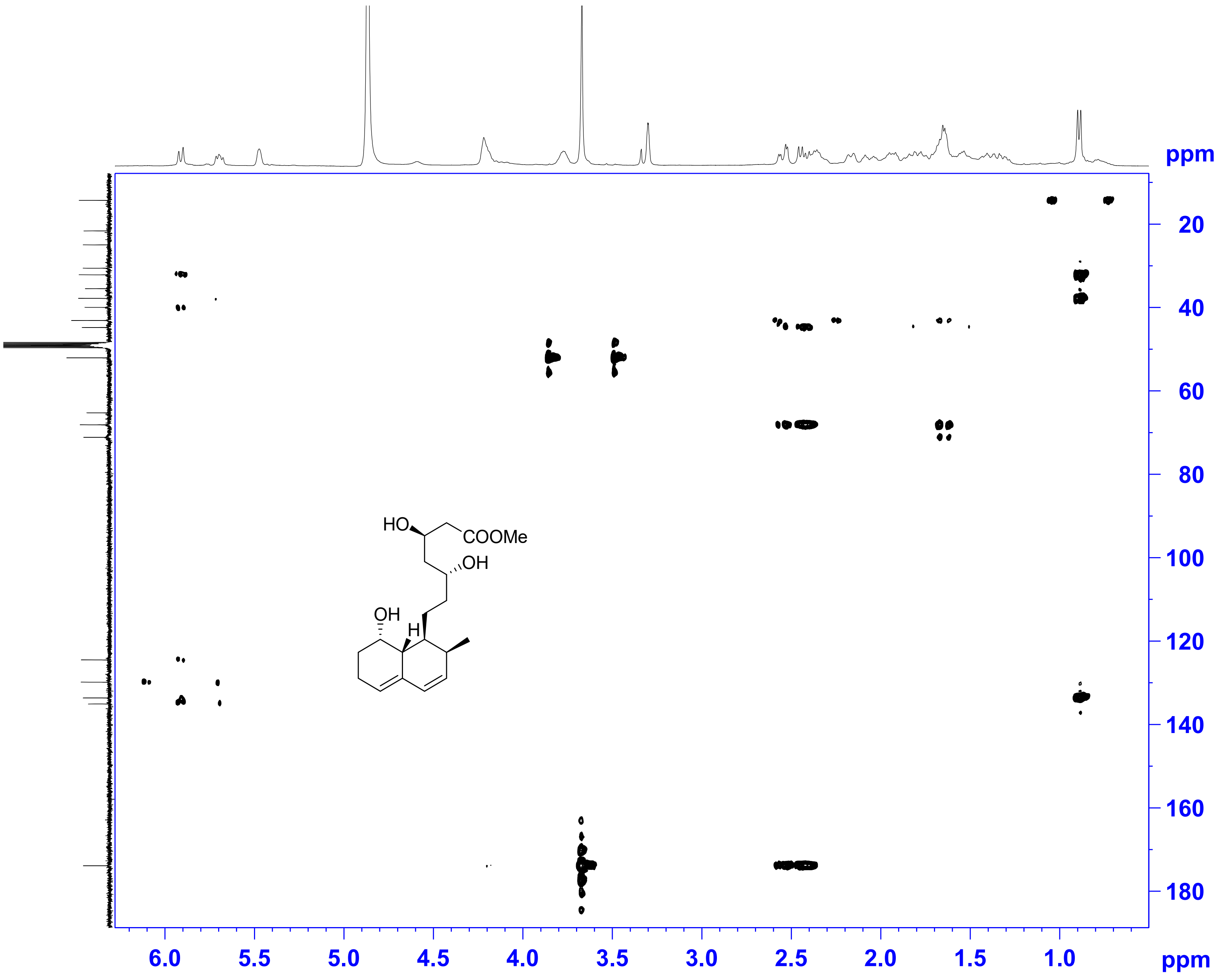

Current Data Parameters
NAME HB2-10 M 10mg
EXPNO 6
PROCNO 1

F2 - Acquisition Parameters
Date_ 20200814
Time 0.29
INSTRUM spect
PROBHD 5 mm PABBO BB-
PULPROG hmbcgpndqf
TD 4096
SOLVENT MeOD
NS 48
DS 16
SWH 3201.024 Hz
FIDRES 0.781500 Hz
AQ 0.6397952 sec
RG 203
DW 156.200 usec
DE 6.50 usec
TE 295.9 K
CNST13 8.0000000
D0 0.00000300 sec
D1 1.50000000 sec
D6 0.06250000 sec
D16 0.00020000 sec
IN0 0.00002480 sec

======== CHANNEL f1 ========
SFO1 400.1316005 MHz
NUC1 1H
P1 13.90 usec
P2 27.80 usec
PLW1 12.14299965 W

======== CHANNEL f2 ========
SFO2 100.6228298 MHz
NUC2 13C
P3 12.37 usec
PLW2 28.13500023 W

====== GRADIENT CHANNEL =====
GPNAM[1] SMSQ10.100
GPNAM[2] SMSQ10.100
GPNAM[3] SMSQ10.100
GPZ1 50.00 %
GPZ2 30.00 %
GPZ3 40.10 %
P16 1000.00 usec

F1 - Acquisition parameters
TD 128
SFO1 100.6228 MHz
FIDRES 157.510086 Hz
SW 200.365 ppm
FnMODE QF

F2 - Processing parameters
SI 1024
SF 400.1300117 MHz
WDW SINE
SSB 0
LB 0 Hz
GB 0
PC 1.40

F1 - Processing parameters
SI 1024
MC2 QF
SF 100.6126294 MHz
WDW SINE
SSB 0
LB 0 Hz
GB 0

Figure S6. NOESY NMR spectrum of 1 in MeOH-d4

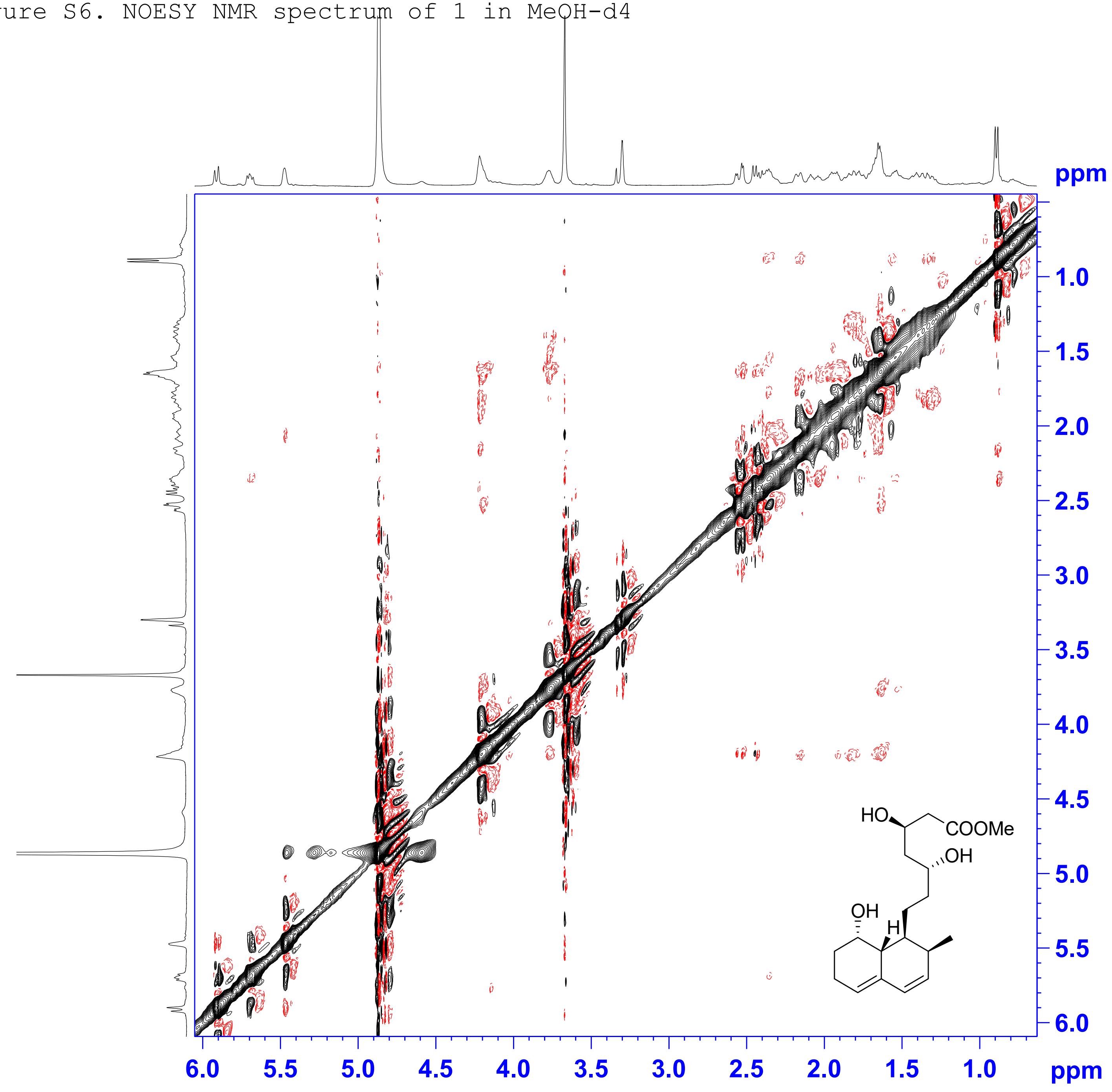


```
Current Data Parameters
NAME       HB2-10 M 10mg
EXPNO                  7
PROCNO                 1

F2 - Acquisition Parameters
Date_           20200814
Time                4.16
INSTRUM            spect
PROBHD    5 mm PABBO BB-
PULPROG      noesygpphpp
TD                  2048
SOLVENT             MeOD
NS                    12
DS                    32
SWH             3201.024 Hz
FIDRES          1.563000 Hz
AQ             0.3198976 sec
RG                   203
DW               156.200 usec
DE                  6.50 usec
TE                 295.9 K
D0            0.00013850 sec
D1            2.00000000 sec
D8            0.30000001 sec
D11           0.03000000 sec
D12           0.00002000 sec
D16           0.00020000 sec
IN0           0.00031240 sec

======== CHANNEL f1 ========
SFO1         400.1316005 MHz
NUC1                  1H
P1                 13.90 usec
P2                 27.80 usec
P17              2500.00 usec
PLW1         12.14299965 W
PLW10         3.47059989 W

====== GRADIENT CHANNEL =====
GPNAM[1]      SMSQ10.100
GPZ1               40.00 %
P16              1000.00 usec

F1 - Acquisition parameters
TD                   256
SFO1            400.1316 MHz
FIDRES         12.504002 Hz
SW                 8.000 ppm
FnMODE       States-TPPI

F2 - Processing parameters
SI                  1024
SF           400.1300117 MHz
WDW                QSINE
SSB                    2
LB             0 Hz
GB             0
PC                  1.00

F1 - Processing parameters
SI                  1024
MC2          States-TPPI
SF           400.1300117 MHz
WDW                QSINE
SSB                    2
LB             0 Hz
GB             0
```

Figure S7. The HR-ESI-MS spectrum of 1.

Single Mass Analysis

Tolerance = 20.0 mDa / DBE: min = -1.5, max = 50.0
Element prediction: Off
Number of isotope peaks used for i-FIT = 3

Monoisotopic Mass, Even Electron Ions
1726 formula(e) evaluated with 73 results within limits (up to 50 closest results for each mass)
Elements Used:
C: 0-50 H: 0-50 N: 0-50 O: 0-50 23Na: 0-1

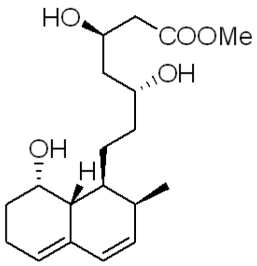

HB 2-10-Aug 148 (0.580) Cm (126:167)
1: TOF MS ES+

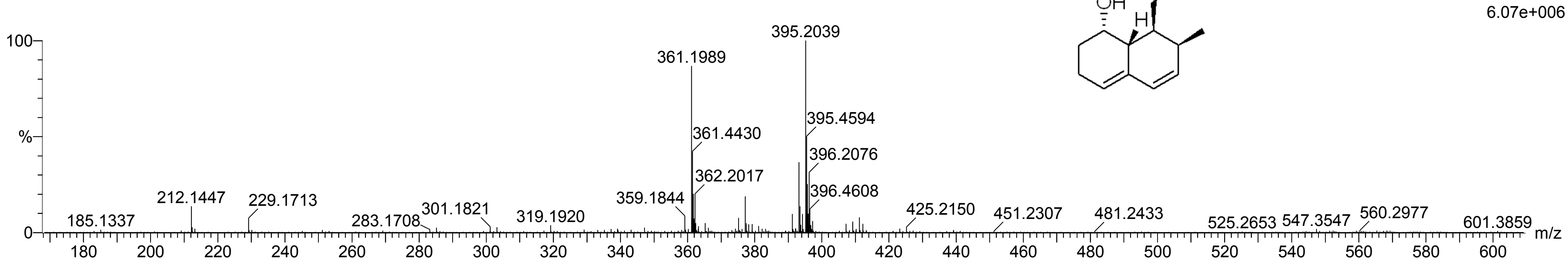

Minimum:				-1.5				
Maximum:		20.0	10.0	50.0				

Mass	Calc. Mass	mDa	PPM	DBE	i-FIT	Norm	Conf(%)	Formula
361.1989	361.1988	0.1	0.3	8.5	1820.9	11.424	0.00	C17 H25 N6 O3
	361.1991	-0.2	-0.6	4.5	1819.2	9.639	0.01	C19 H30 O5 23Na
	361.1993	-0.4	-1.1	1.5	1829.6	20.052	0.00	C2 H21 N18 O4
	361.1982	0.7	1.9	3.5	1832.5	22.942	0.00	C H18 N22 23Na
	361.1975	1.4	3.9	3.5	1822.6	13.089	0.00	C16 H29 N2 O7
	361.2004	-1.5	-4.2	9.5	1818.9	9.344	0.01	C20 H26 N4 O 23Na
	361.2007	-1.8	-5.0	6.5	1831.2	21.703	0.00	C3 H17 N22
	361.1969	2.0	5.5	-1.5	1830.3	20.761	0.00	H22 N18 O4 23Na
	361.2009	-2.0	-5.5	2.5	1829.1	19.615	0.00	C5 H22 N16 O2 23Na
	361.1964	2.5	6.9	5.5	1823.3	13.822	0.00	C15 H26 N6 O3 23Na
	361.2015	-2.6	-7.2	7.5	1809.5	0.001	99.93	C21 H29 O5
	361.1961	2.8	7.8	9.5	1824.8	15.318	0.00	C13 H21 N12 O
	361.2020	-3.1	-8.6	0.5	1827.9	18.329	0.00	C6 H25 N12 O6
	361.1956	3.3	9.1	16.5	1822.6	13.047	0.00	C28 H25
	361.1951	3.8	10.5	0.5	1824.4	14.914	0.00	C14 H30 N2 O7 23Na
	361.2028	-3.9	-10.8	12.5	1819.6	10.036	0.00	C22 H25 N4 O
	361.1948	4.1	11.4	4.5	1825.2	15.633	0.00	C12 H25 N8 O5
	361.2033	-4.4	-12.2	5.5	1828.2	18.671	0.00	C7 H21 N16 O2
	361.2036	-4.7	-13.0	1.5	1827.0	17.427	0.00	C9 H26 N10 O4 23Na
	361.1937	5.2	14.4	6.5	1826.3	16.798	0.00	C11 H22 N12 O 23Na
	361.1935	5.4	15.0	-0.5	1826.0	16.509	0.00	C11 H29 N4 O9
	361.1932	5.7	15.8	13.5	1821.2	11.701	0.00	C26 H26 23Na
	361.2047	-5.8	-16.1	-0.5	1826.4	16.867	0.00	C10 H29 N6 O8
	361.2050	-6.1	-16.9	6.5	1827.2	17.710	0.00	C10 H22 N14 23Na
	361.1924	6.5	18.0	1.5	1826.4	16.889	0.00	C10 H26 N8 O5 23Na
	361.1921	6.8	18.8	5.5	1827.5	17.930	0.00	C8 H21 N14 O3
	361.2060	-7.1	-19.7	4.5	1825.8	16.301	0.00	C11 H25 N10 O4

Figure S8. 1H NMR spectrum of 2 in DMSO-d6.

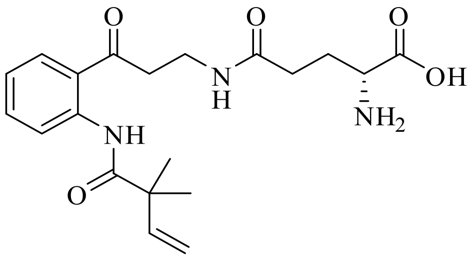

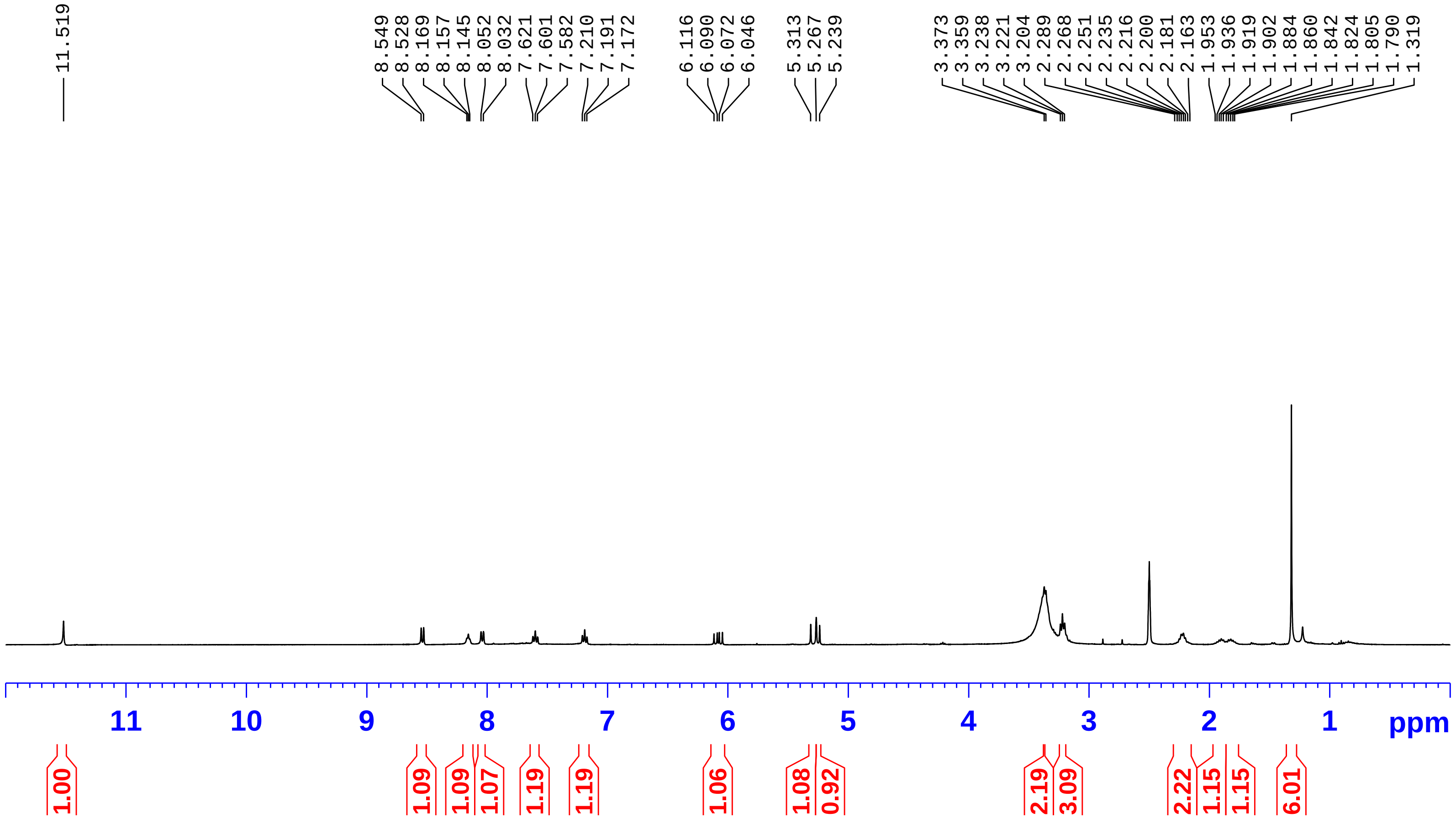

NAME	HB4-6 D 5mg
EXPNO	1
PROCNO	1
Date_	20200108
Time	9.18
INSTRUM	spect
PROBHD	5 mm PABBO BB-
PULPROG	zg30
TD	65536
SOLVENT	DMSO
NS	28
DS	2
SWH	8012.820 Hz
FIDRES	0.122266 Hz
AQ	4.0894966 sec
RG	203
DW	62.400 usec
DE	6.50 usec
TE	292.5 K
D1	1.00000000 sec
TD0	1

======== CHANNEL f1 ========

SFO1	400.1324710 MHz
NUC1	1H
P1	13.90 usec
SI	32768
SF	400.1300029 MHz
WDW	EM
SSB	0
LB	0.30 Hz
GB	0
PC	1.00

Figure S9. 13C NMR spectrum of 2 in DMSO-d6.

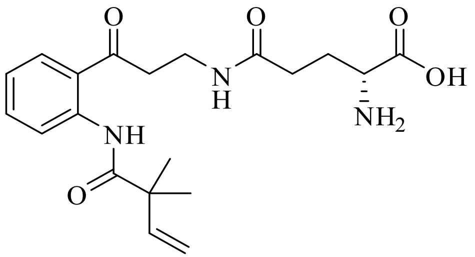

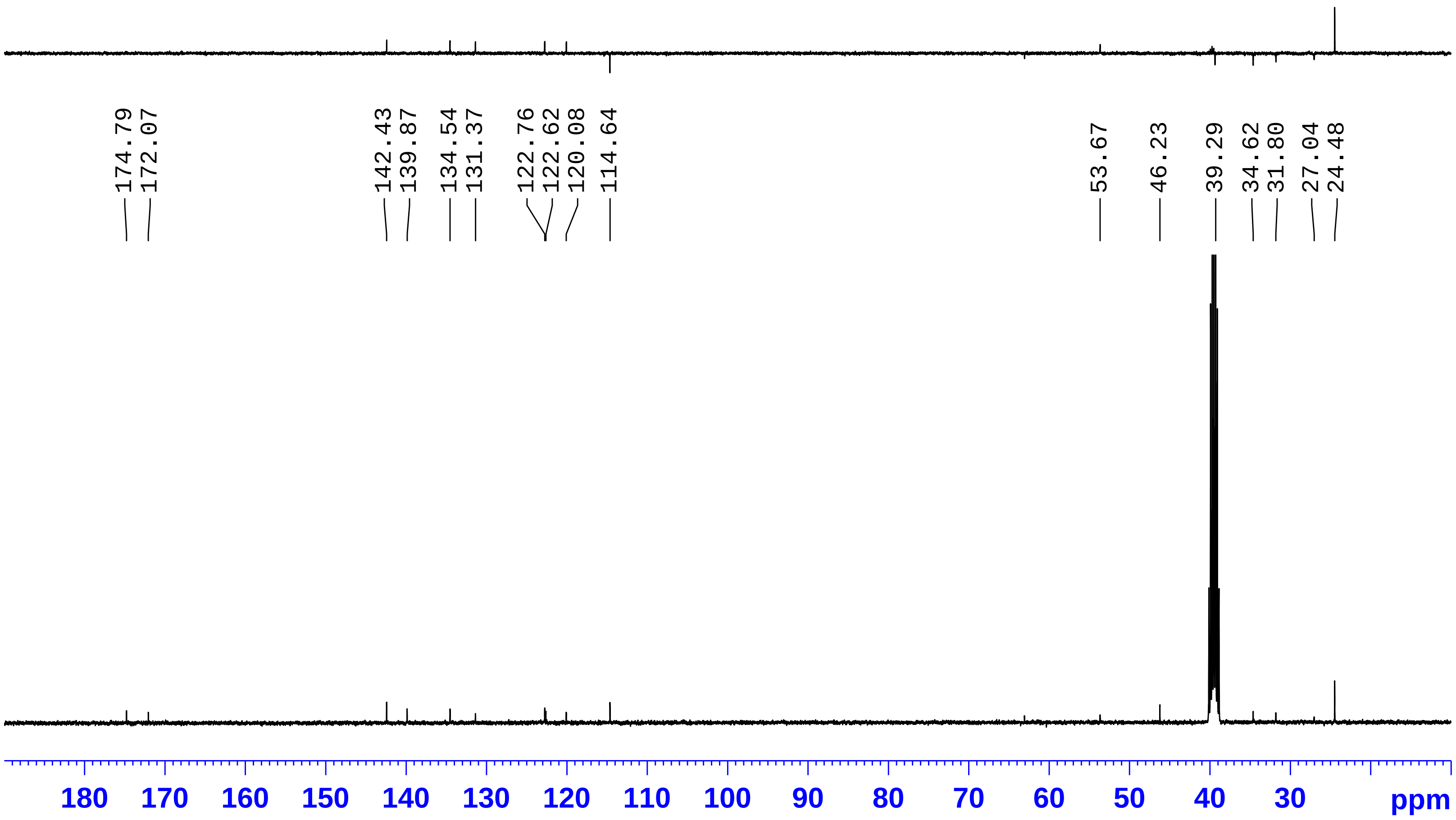

BRUKER

NAME	HB4-6 D 5mg	
EXPNO	2	
PROCNO	1	
Date_	20200108	
Time	9.23	
INSTRUM	spect	
PROBHD	5 mm PABBO BB-	
PULPROG	zgpg30	
TD	65536	
SOLVENT	DMSO	
NS	1229	
DS	4	
SWH	24038.461	Hz
FIDRES	0.366798	Hz
AQ	1.3631988	sec
RG	203	
DW	20.800	usec
DE	6.50	usec
TE	292.6	K
D1	2.00000000	sec
D11	0.03000000	sec
TD0	1	

======== CHANNEL f1 ========

SFO1	100.6228293	MHz
NUC1	13C	
P1	12.37	usec
SI	32768	
SF	100.6128134	MHz
WDW	EM	
SSB	0	
LB	1.00	Hz
GB	0	
PC	1.40	

Figure S10. HSQC NMR spectrum of 2 in DMSO-d6.

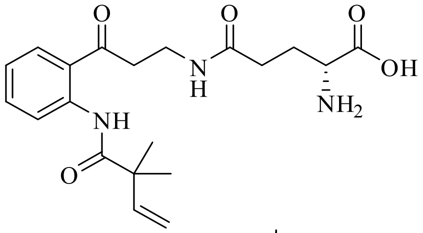

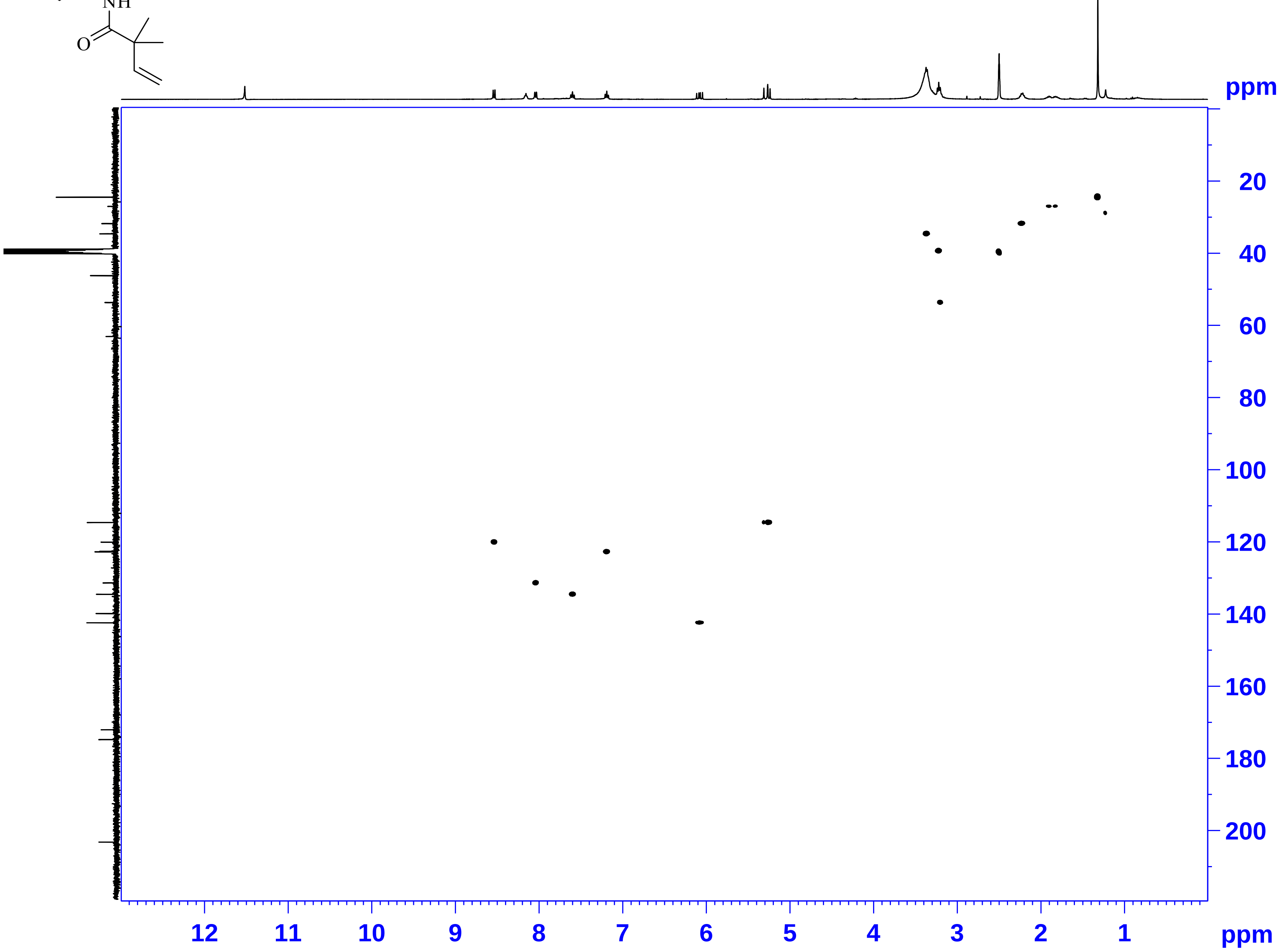

NAME	HB4-6 D 5mg	
EXPNO	4	
PROCNO	1	
Date_	20200111	
Time	18.48	
INSTRUM	spect	
PROBHD	5 mm PABBO BB-	
PULPROG	hsqcetgpsisp2.2	
TD	1024	
SOLVENT	DMSO	
NS	8	
DS	32	
SWH	5197.505	Hz
FIDRES	5.075689	Hz
AQ	0.0985588	sec
RG	203	
DW	96.200	usec
DE	6.50	usec
TE	294.1	K
CNST2	145.0000000	
CNST17	-0.5000000	
D0	0.00000300	sec
D1	2.00000000	sec
D4	0.00172414	sec
D11	0.03000000	sec
D16	0.00020000	sec
D24	0.00089000	sec
IN0	0.00002260	sec
======== CHANNEL f1 ========		
SFO1	400.1326008	MHz
NUC1	1H	
P1	13.90	usec
P2	27.80	usec
P28	1000.00	usec
ND0	2	
TD	256	
SFO1	100.6238	MHz
FIDRES	86.421463	Hz
SW	219.867	ppm
FnMODE	Echo-Antiecho	
SI	1024	
SF	400.1299999	MHz
WDW	QSINE	
SSB	2	
LB	0.00	Hz
GB	0	
PC	1.40	
SI	1024	
MC2	echo-antiecho	
SF	100.6128137	MHz
WDW	QSINE	
SSB	2	
LB	0.00	Hz
GB	0	

Figure S11. COSY NMR spectrum of 2 in DMSO-d6.

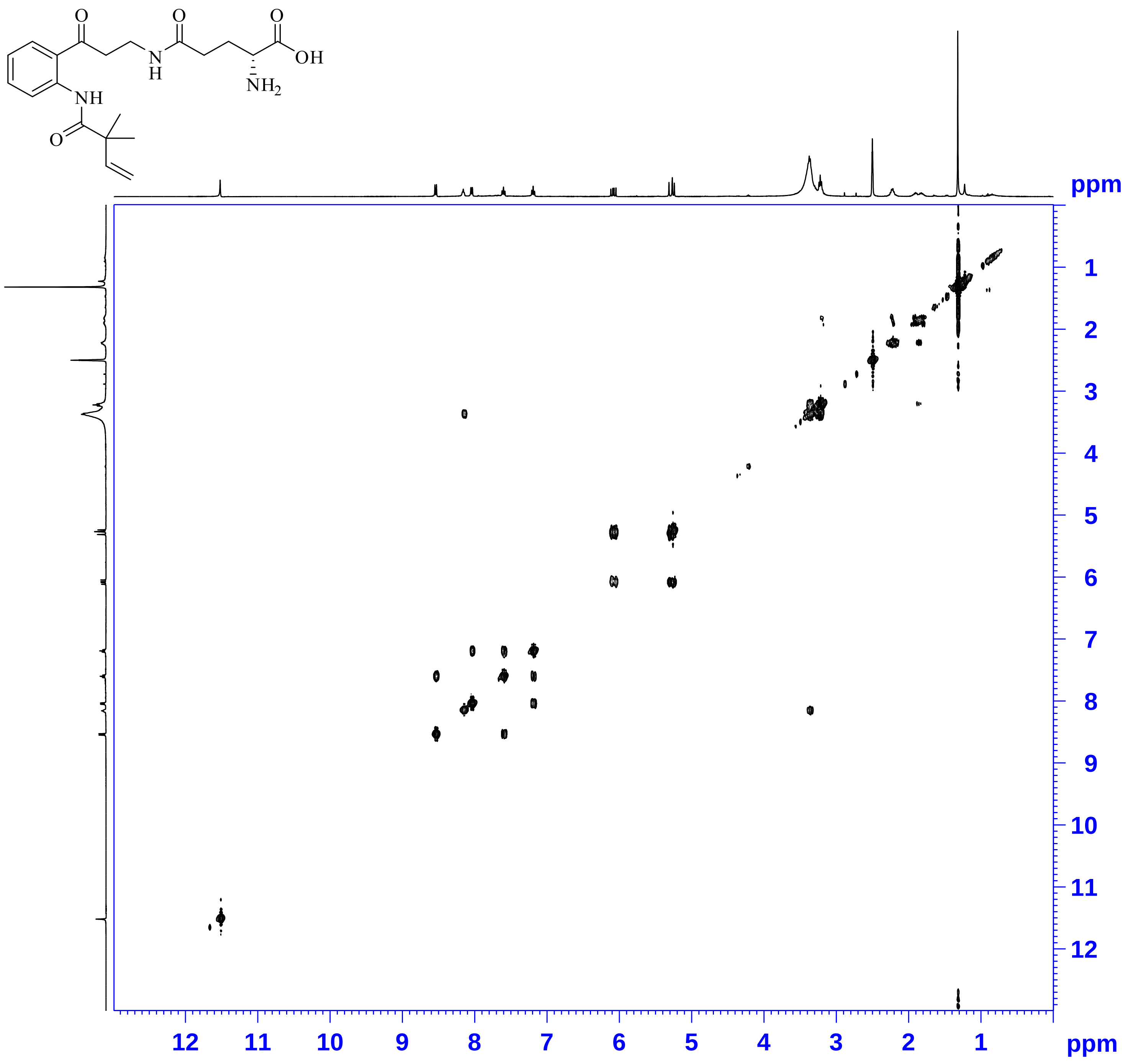

NAME	HB4-6 D 5mg
EXPNO	5
PROCNO	1
Date_	20200111
Time	10.19
INSTRUM	spect
PROBHD	5 mm PABBO BB-
PULPROG	cosygpqf
TD	2048
SOLVENT	DMSO
NS	16
DS	8
SWH	5197.505 Hz
FIDRES	2.537844 Hz
AQ	0.1970676 sec
RG	203
DW	96.200 usec
DE	6.50 usec
TE	293.8 K
D0	0.00000300 sec
D1	1.50000000 sec
D13	0.00000400 sec
D16	0.00020000 sec
IN0	0.00019220 sec
======== CHANNEL f1 ========	
SFO1	400.1326008 MHz
NUC1	1H
P0	13.90 usec
P1	13.90 usec
ND0	1
TD	128
SFO1	400.1326 MHz
FIDRES	40.647762 Hz
SW	13.003 ppm
FnMODE	QF
SI	1024
SF	400.1300030 MHz
WDW	SINE
SSB	0
LB	0.00 Hz
GB	0
PC	1.40
SI	1024
MC2	QF
SF	400.1300030 MHz
WDW	SINE
SSB	0
LB	0.00 Hz
GB	0

Figure S12. HMBC NMR spectrum of 2 in DMSO-d6.

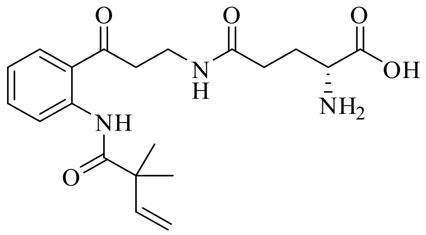

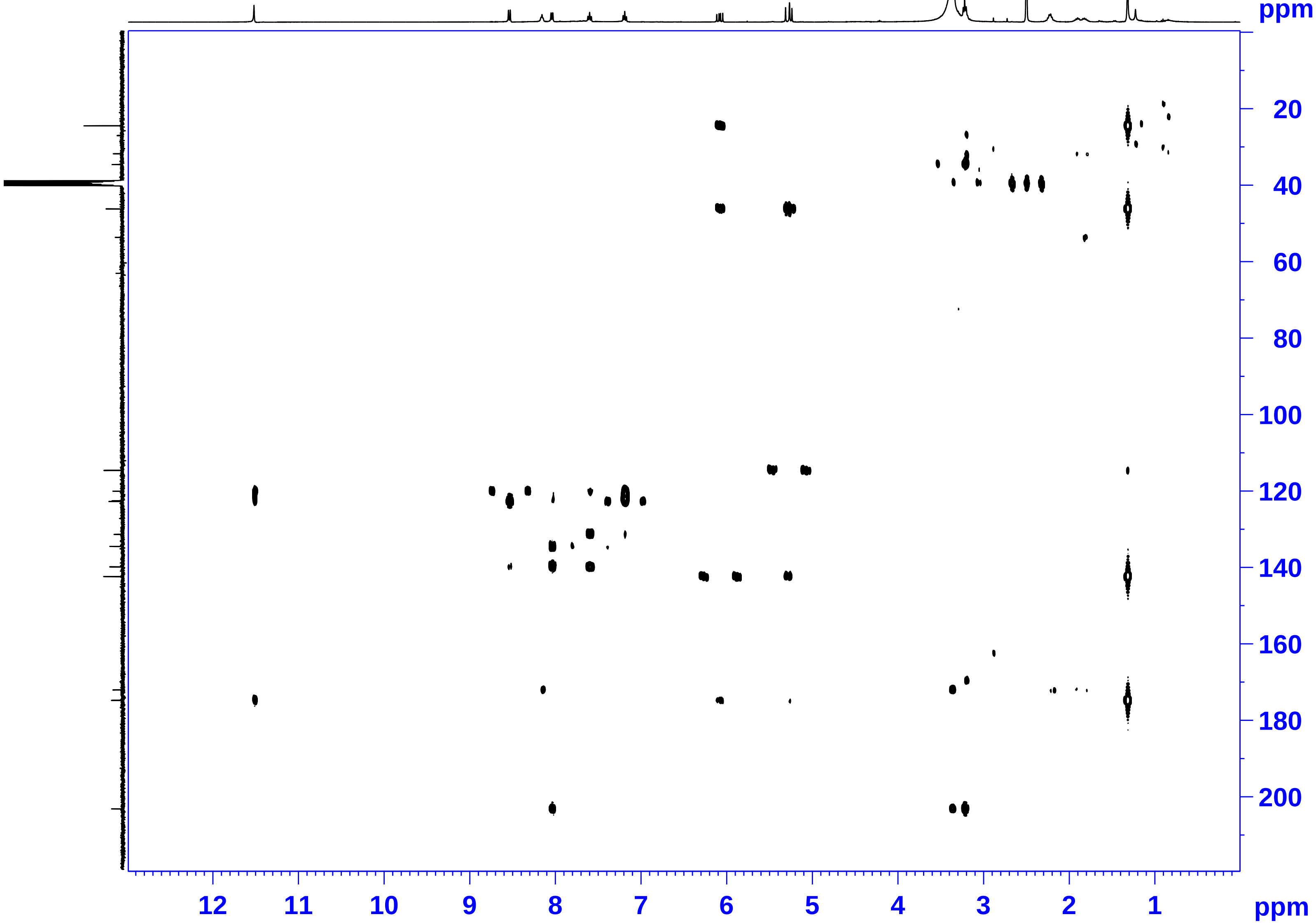

NAME	HB4-6 D 5mg	
EXPNO	6	
PROCNO	1	
Date_	20200111	
Time	11.20	
INSTRUM	spect	
PROBHD	5 mm PABBO BB-	
PULPROG	hmbcgpndqf	
TD	4096	
SOLVENT	DMSO	
NS	64	
DS	16	
SWH	5197.505	Hz
FIDRES	1.268922	Hz
AQ	0.3940852	sec
RG	203	
DW	96.200	usec
DE	6.50	usec
TE	293.8	K
CNST13	8.0000000	
D0	0.00000300	sec
D1	1.50000000	sec
D6	0.06250000	sec
D16	0.00020000	sec
IN0	0.00002260	sec

======== CHANNEL f1 ========

SFO1	400.1326008	MHz
NUC1	1H	
P1	13.90	usec
P2	27.80	usec
ND0	2	
TD	128	
SFO1	100.6238	MHz
FIDRES	172.842926	Hz
SW	219.867	ppm
FnMODE	QF	
SI	1024	
SF	400.1300030	MHz
WDW	SINE	
SSB	0	
LB	0.00	Hz
GB	0	
PC	1.40	
SI	1024	
MC2	QF	
SF	100.6128137	MHz
WDW	SINE	
SSB	0	
LB	0.00	Hz
GB	0	

Figure S13. NOESY NMR spectrum of 2 in DMSO-d6.

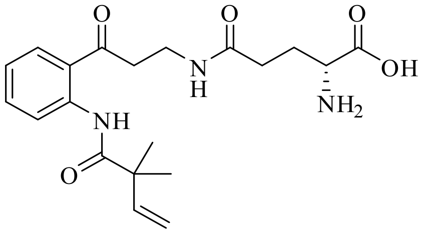

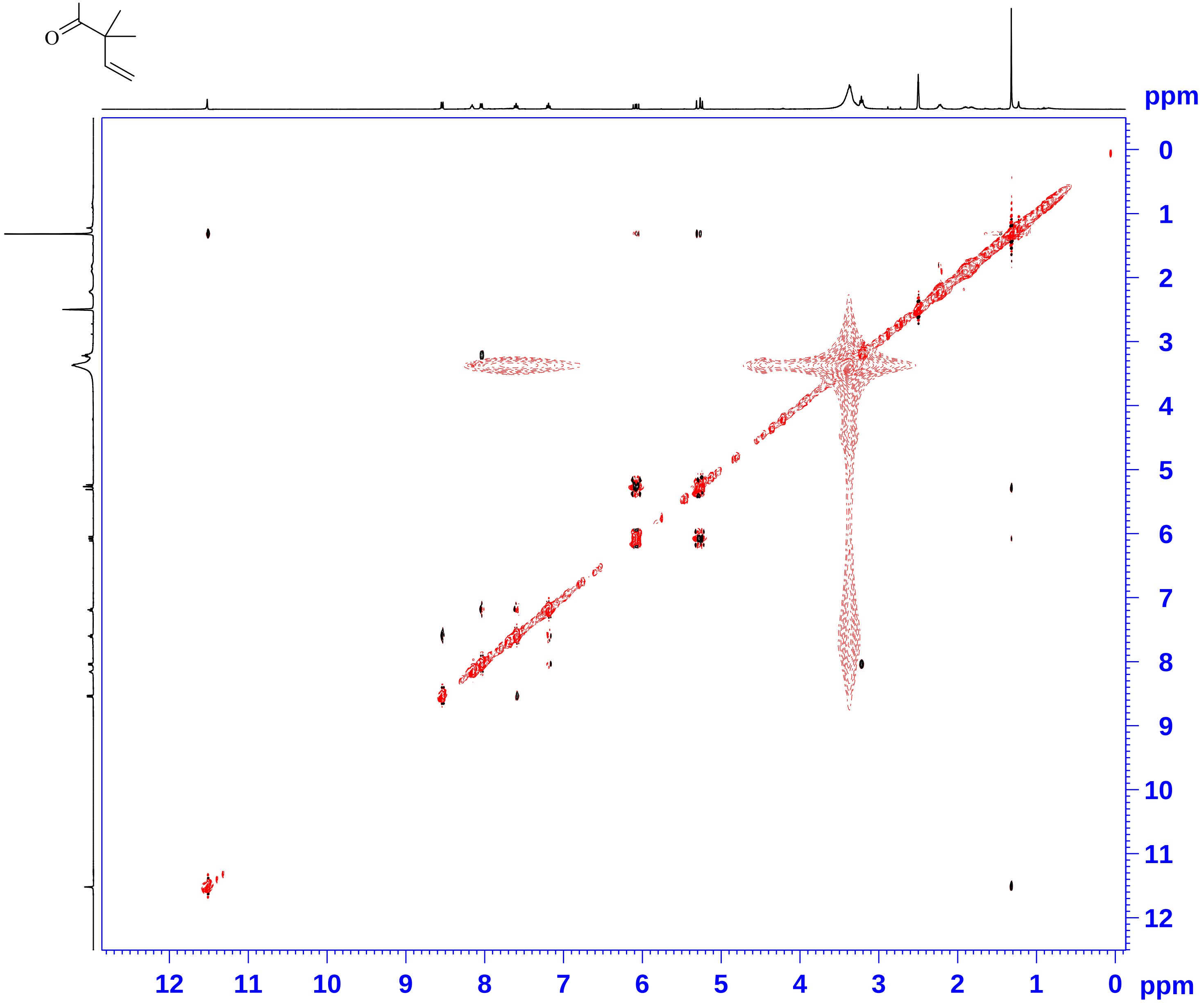

NAME	HB4-6 D 5mg	
EXPNO	7	
PROCNO	1	
Date_	20200111	
Time	15.49	
INSTRUM	spect	
PROBHD	5 mm PABBO BB-	
PULPROG	noesygpphpp	
TD	2048	
SOLVENT	DMSO	
NS	16	
DS	32	
SWH	5197.505	Hz
FIDRES	2.537844	Hz
AQ	0.1970676	sec
RG	203	
DW	96.200	usec
DE	6.50	usec
TE	294.0	K
D0	0.00007840	sec
D1	2.00000000	sec
D8	0.30000001	sec
D11	0.03000000	sec
D12	0.00002000	sec
D16	0.00020000	sec
IN0	0.00019220	sec
======== CHANNEL f1 ========		
SFO1	400.1326008	MHz
NUC1	1H	
P1	13.90	usec
P2	27.80	usec
P17	2500.00	usec
ND0	1	
TD	256	
SFO1	400.1326	MHz
FIDRES	20.323881	Hz
SW	13.003	ppm
FnMODE	States-TPPI	
SI	1024	
SF	400.1300030	MHz
WDW	QSINE	
SSB	2	
LB	0.00	Hz
GB	0	
PC	1.00	
SI	1024	
MC2	States-TPPI	
SF	400.1300030	MHz
WDW	QSINE	
SSB	2	
LB	0.00	Hz
GB	0	

Figure S14. The HR-ESI-MS spectrum of 2.

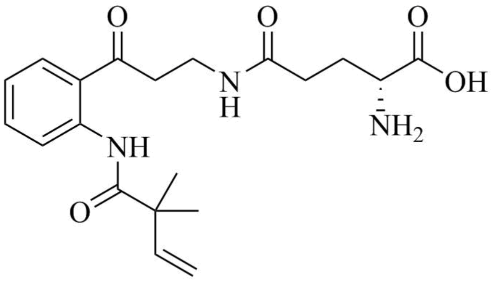

Chemical Formula: $C_{20}H_{27}N_3O_5$
Molecular Weight: 389.4520

Single Mass Analysis

Tolerance = 10.0 PPM / DBE: min = -1.5, max = 50.0
Element prediction: Off
Number of isotope peaks used for i-FIT = 3

Monoisotopic Mass, Even Electron Ions
626 formula(e) evaluated with 2 results within limits (up to 50 closest results for each mass)
Elements Used:
C: 10-20 H: 1-100 N: 0-20 O: 0-30
HB 4-7-N 105 (0.415)
1: TOF MS ES-

7.62e+004

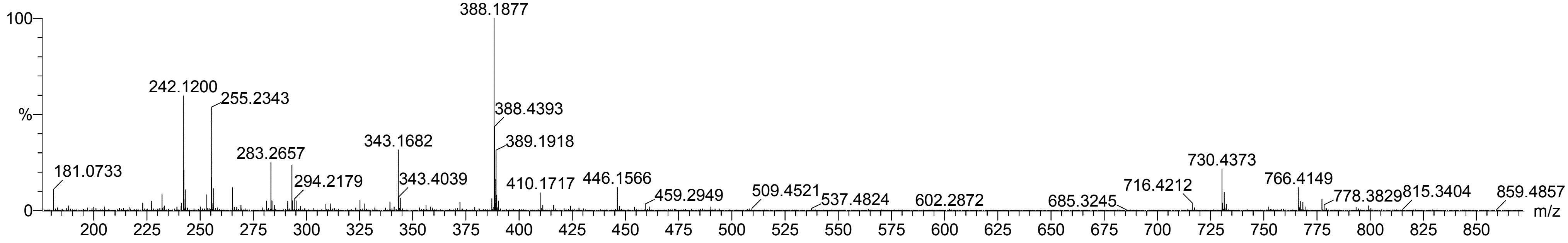

Minimum:				-1.5				
Maximum:		50.0	10.0	50.0				

Mass	Calc. Mass	mDa	PPM	DBE	i-FIT	Norm	Conf(%)	Formula
388.1877	388.1872	0.5	1.3	9.5	565.4	0.012	98.81	C20 H26 N3 O5
	388.1846	3.1	8.0	10.5	569.8	4.427	1.19	C16 H22 N9 O3

Figure S15. 1H NMR spectrum of 3 in MeOH-d4

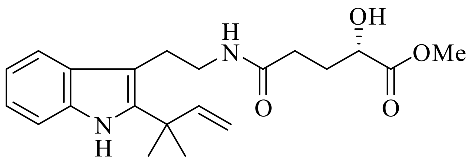

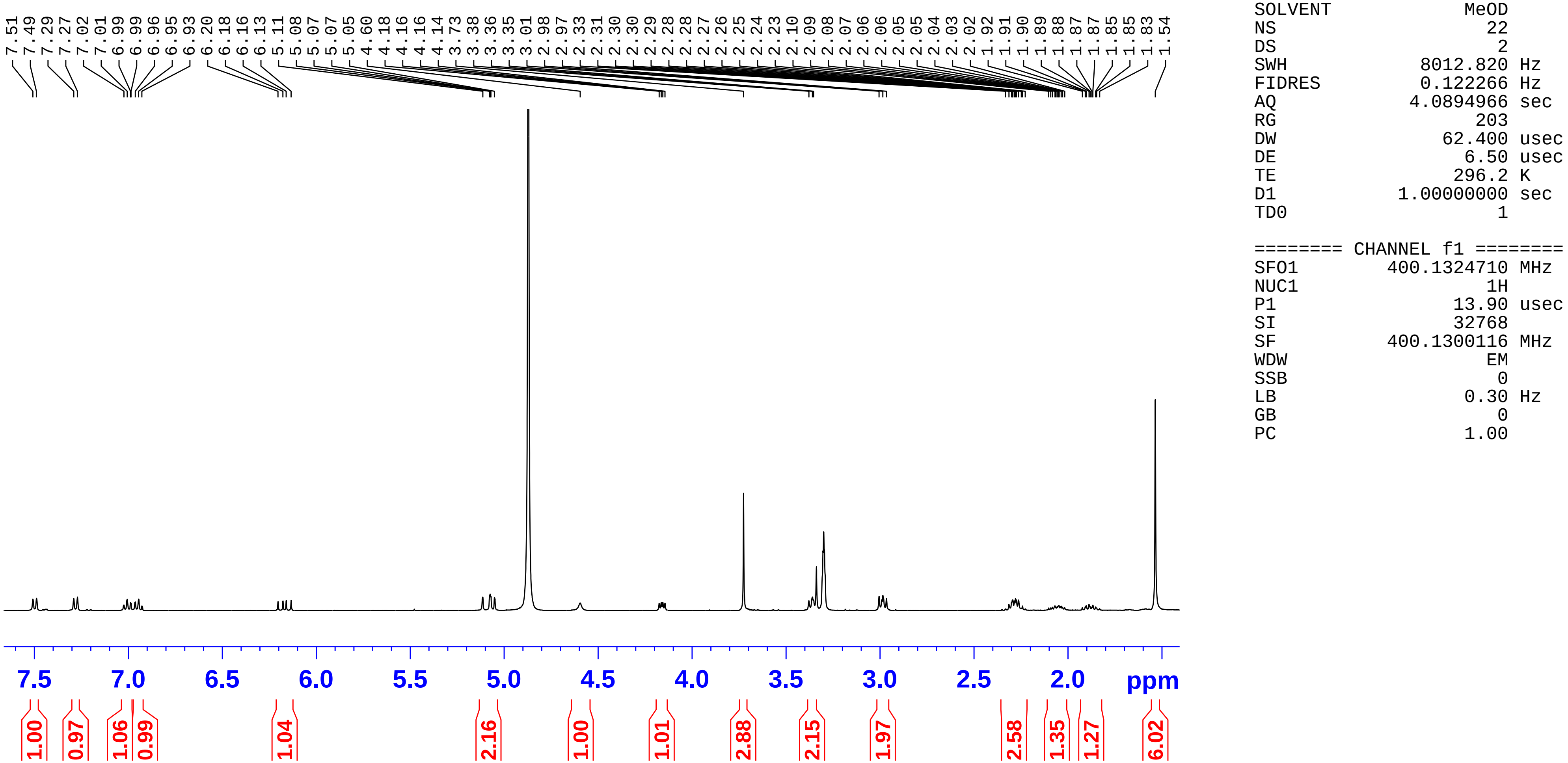

Figure S16. 13C NMR spectrum of 3 in MeOH-d4

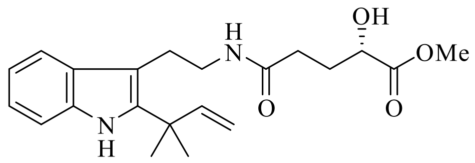

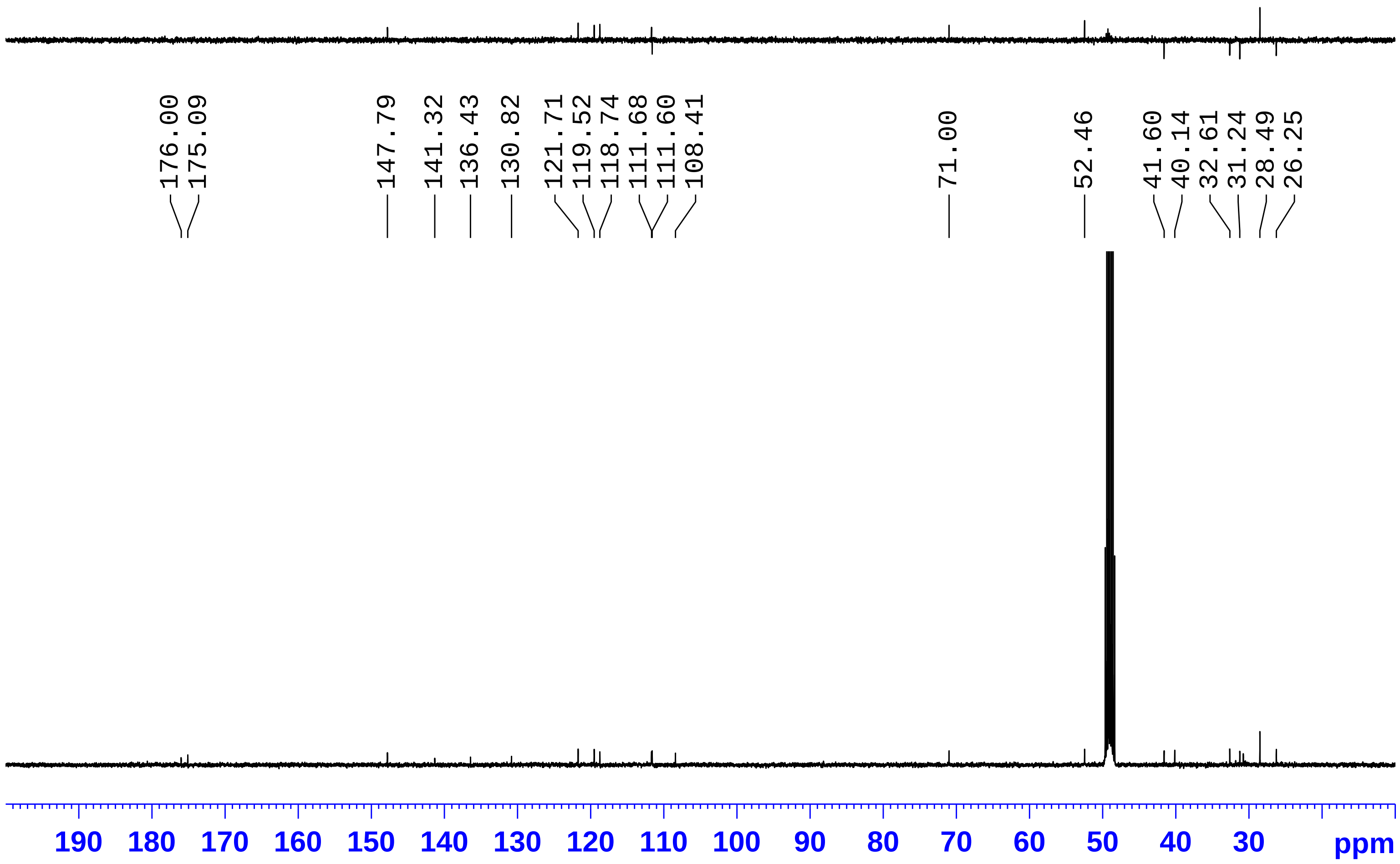

BRUKER

NAME	HB2-12 M 3mg
EXPNO	2
PROCNO	1
Date_	20200805
Time	16.33
INSTRUM	spect
PROBHD	5 mm PABBO BB-
PULPROG	zgpg30
TD	65536
SOLVENT	MeOD
NS	1045
DS	4
SWH	24038.461 Hz
FIDRES	0.366798 Hz
AQ	1.3631988 sec
RG	203
DW	20.800 usec
DE	6.50 usec
TE	296.3 K
D1	2.00000000 sec
D11	0.03000000 sec
TD0	1

======== CHANNEL f1 ========

SFO1	100.6228293 MHz
NUC1	13C
P1	12.37 usec
SI	32768
SF	100.6126277 MHz
WDW	EM
SSB	0
LB	1.00 Hz
GB	0
PC	1.40

Figure S17. HSQC NMR spectrum of 3 in MeOH-d4

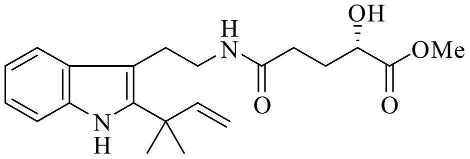

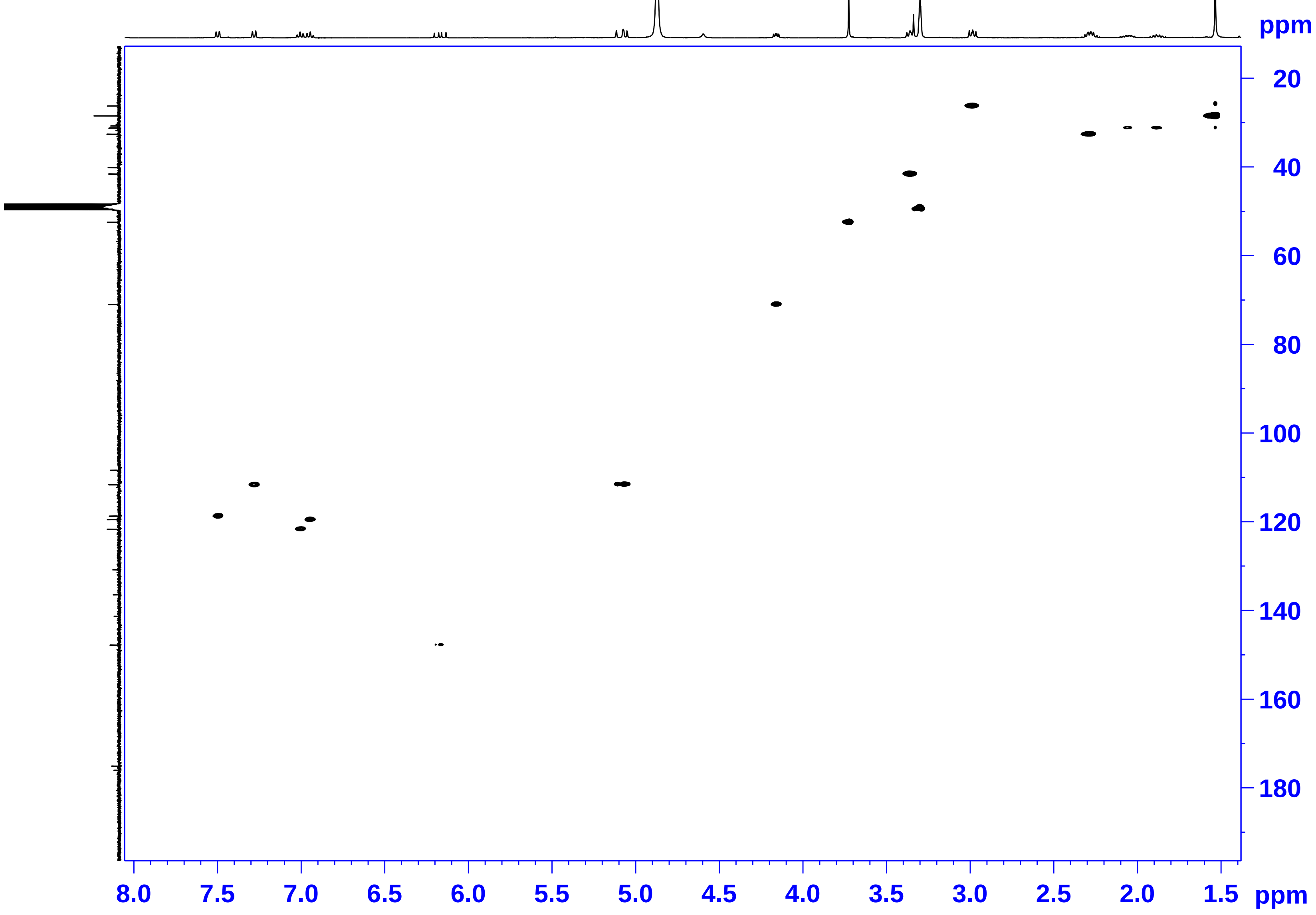


```
NAME           HB2-12 M 3mg
EXPNO                     4
PROCNO                    1
Date_              20200809
Time                  13.43
INSTRUM               spect
PROBHD      5 mm PABBO BB-
PULPROG    hsqcetgpsisp2.2
TD                     1024
SOLVENT                MeOD
NS                       32
DS                       32
SWH                3597.122 Hz
FIDRES             3.512815 Hz
AQ                0.1423860 sec
RG                      203
DW                  139.000 usec
DE                     6.50 usec
TE                    296.6 K
CNST2           145.0000000
CNST17           -0.5000000
D0               0.00000300 sec
D1               2.00000000 sec
D4               0.00172414 sec
D11              0.03000000 sec
D16              0.00020000 sec
D24              0.00089000 sec
IN0              0.00002480 sec

======== CHANNEL f1 ========
SFO1            400.1318006 MHz
NUC1                     1H
P1                    13.90 usec
P2                    27.80 usec
P28                 1000.00 usec
ND0                       2
TD                      256
SFO1               100.6228 MHz
FIDRES            78.755043 Hz
SW                  200.365 ppm
FnMODE         Echo-Antiecho
SI                     1024
SF              400.1300116 MHz
WDW                   QSINE
SSB                       2
LB                     0.00 Hz
GB                        0
PC                     1.40
SI                     1024
MC2            echo-antiecho
SF              100.6126277 MHz
WDW                   QSINE
SSB                       2
LB                     0.00 Hz
GB                        0
```

Figure S18. HSQC NMR spectrum of 3 in MeOH-d4

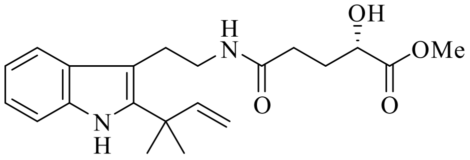

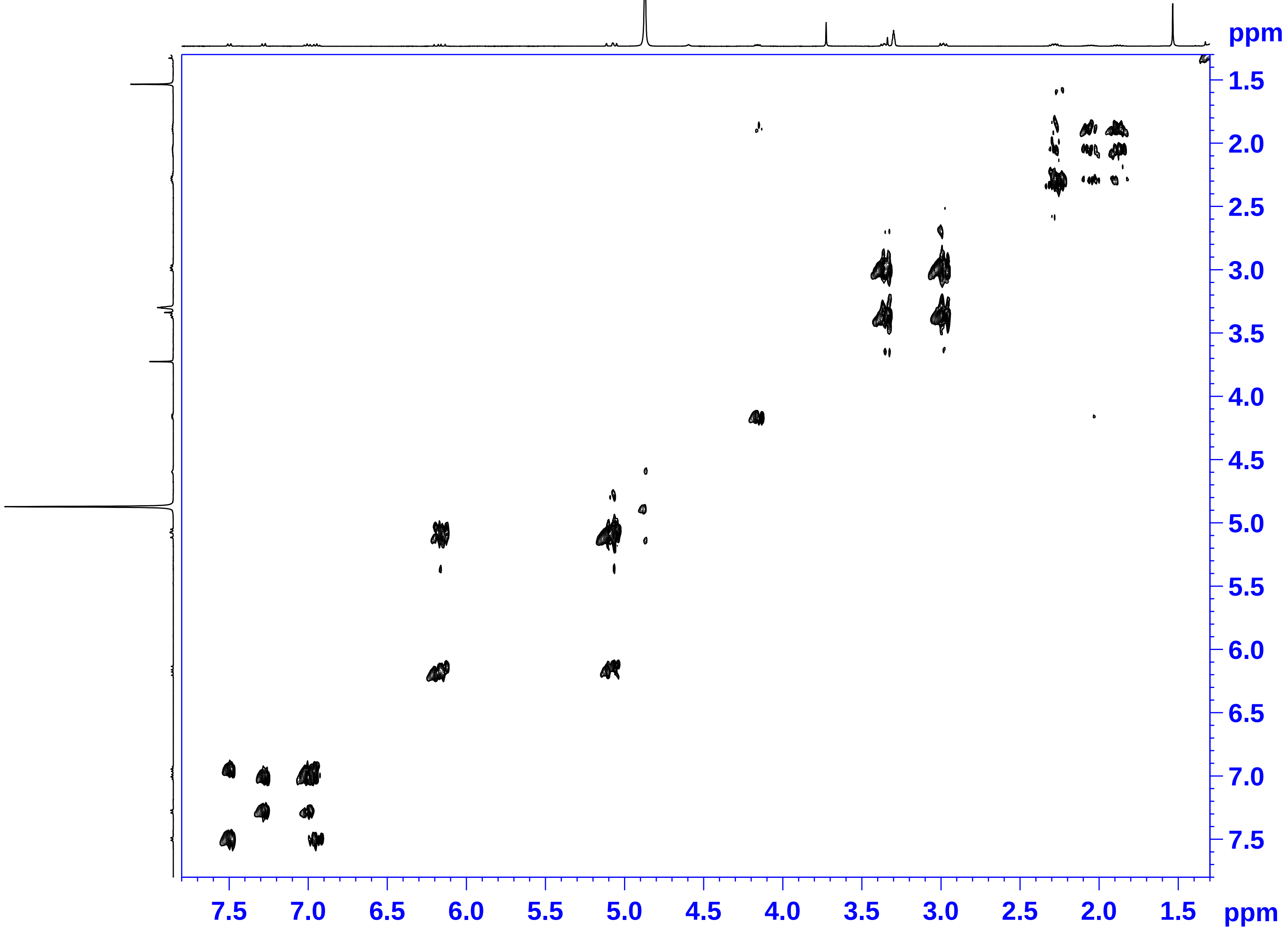

BRUKER

NAME	HB2-12 M 3mg
EXPNO	5
PROCNO	1
Date_	20200809
Time	18.40
INSTRUM	spect
PROBHD	5 mm PABBO BB-
PULPROG	cosygpmfqf
TD	2048
SOLVENT	MeOD
NS	32
DS	8
SWH	3597.122 Hz
FIDRES	1.756407 Hz
AQ	0.2847220 sec
RG	203
DW	139.000 usec
DE	6.50 usec
TE	296.3 K
D0	0.00000300 sec
D1	2.00000000 sec
D13	0.00000400 sec
D16	0.00020000 sec
IN0	0.00027760 sec
======== CHANNEL f1 ========	
SFO1	400.1318006 MHz
NUC1	1H
P1	13.90 usec
ND0	1
TD	128
SFO1	400.1318 MHz
FIDRES	28.143011 Hz
SW	9.003 ppm
FnMODE	QF
SI	1024
SF	400.1300116 MHz
WDW	SINE
SSB	0
LB	0.00 Hz
GB	0
PC	1.40
SI	1024
MC2	QF
SF	400.1300116 MHz
WDW	SINE
SSB	0
LB	0.00 Hz
GB	0

Figure S19. HMBC NMR spectrum of 3 in MeOH-d4

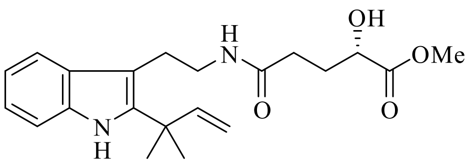

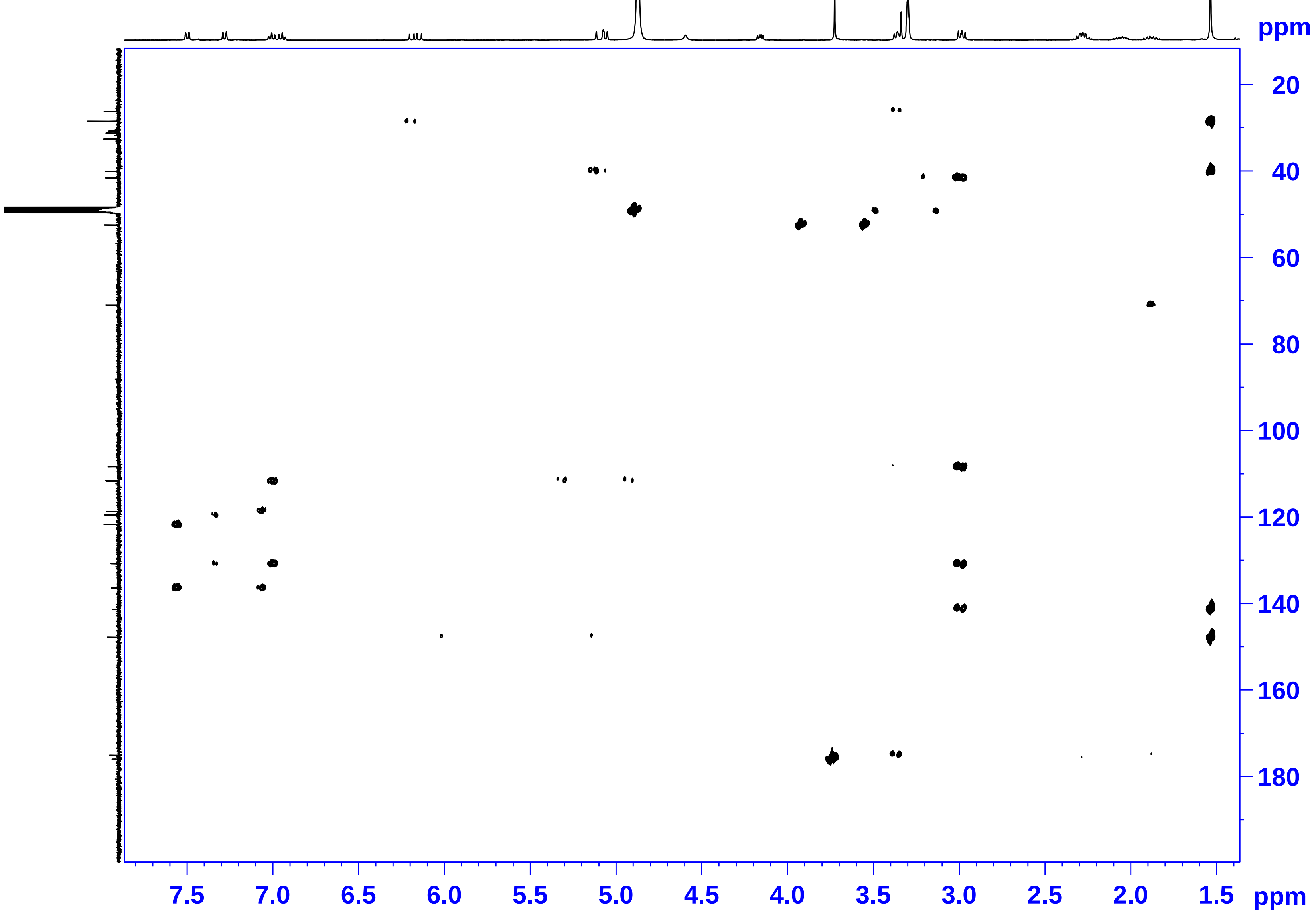

NAME	HB2-12 M 3mg	
EXPNO	6	
PROCNO	1	
Date_	20200809	
Time	21.19	
INSTRUM	spect	
PROBHD	5 mm PABBO BB-	
PULPROG	hmbcgpndqf	
TD	4096	
SOLVENT	MeOD	
NS	64	
DS	16	
SWH	3597.122	Hz
FIDRES	0.878204	Hz
AQ	0.5693940	sec
RG	203	
DW	139.000	usec
DE	6.50	usec
TE	295.9	K
CNST13	8.0000000	
D0	0.00000300	sec
D1	1.50000000	sec
D6	0.06250000	sec
D16	0.00020000	sec
IN0	0.00002480	sec
======== CHANNEL f1 ========		
SFO1	400.1318006	MHz
NUC1	1H	
P1	13.90	usec
P2	27.80	usec
ND0	2	
TD	128	
SFO1	100.6228	MHz
FIDRES	157.510086	Hz
SW	200.365	ppm
FnMODE	QF	
SI	1024	
SF	400.1300116	MHz
WDW	SINE	
SSB	0	
LB	0.00	Hz
GB	0	
PC	1.40	
SI	1024	
MC2	QF	
SF	100.6126277	MHz
WDW	SINE	
SSB	0	
LB	0.00	Hz
GB	0	

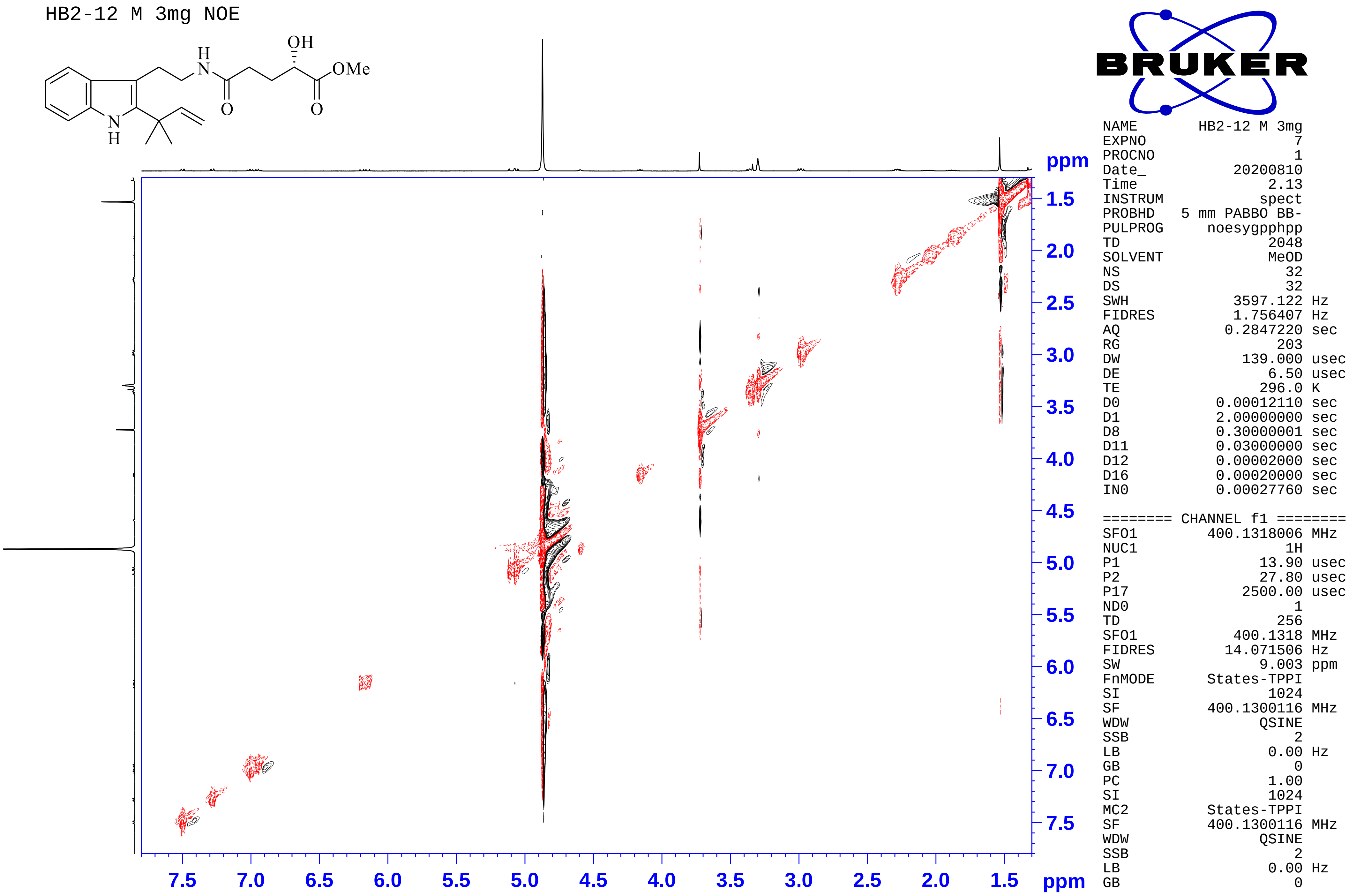

Figure S20. NOESY NMR spectrum of **3** in MeOH-d_4.

Single Mass Analysis

Tolerance = 10.0 PPM / DBE: min = -1.5, max = 50.0
Element prediction: Off
Number of isotope peaks used for i-FIT = 3

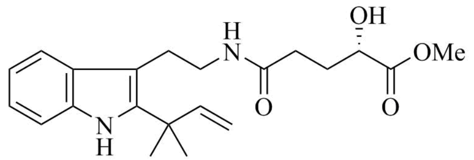

Chemical Formula: $C_{21}H_{28}N_2O_4$
Molecular Weight: 372.4650

Monoisotopic Mass, Even Electron Ions
700 formula(e) evaluated with 1 results within limits (up to 50 closest results for each mass)
Elements Used:
C: 19-20 H: 1-100 N: 0-20 O: 0-30 23Na: 0-1

HB 2-12-Oct 92 (0.368)
1: TOF MS ES+

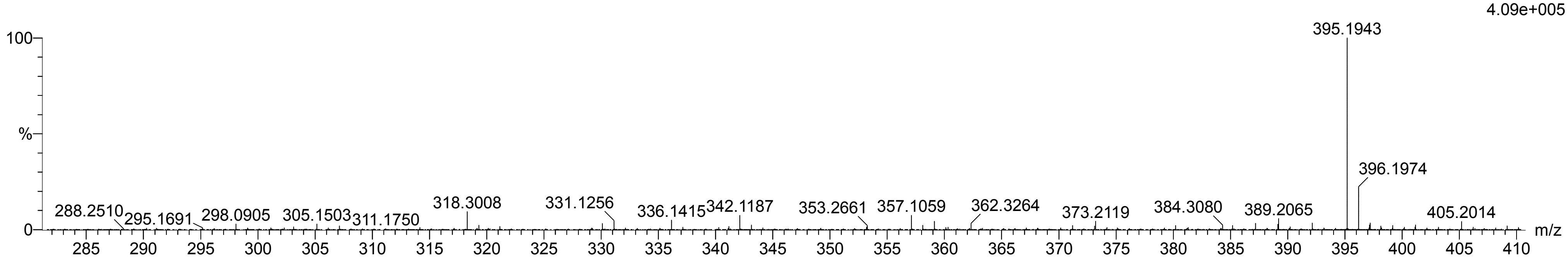

Minimum:				-1.5				
Maximum:		50.0	10.0	50.0				
Mass	Calc. Mass	mDa	PPM	DBE	i-FIT	Norm	Conf(%)	Formula
395.1943	395.1944	-0.1	-0.3	12.5	775.7	n/a	n/a	C19 H23 N8 O2

Figure S21. The HR-ESI-MS spectrum of **3**.

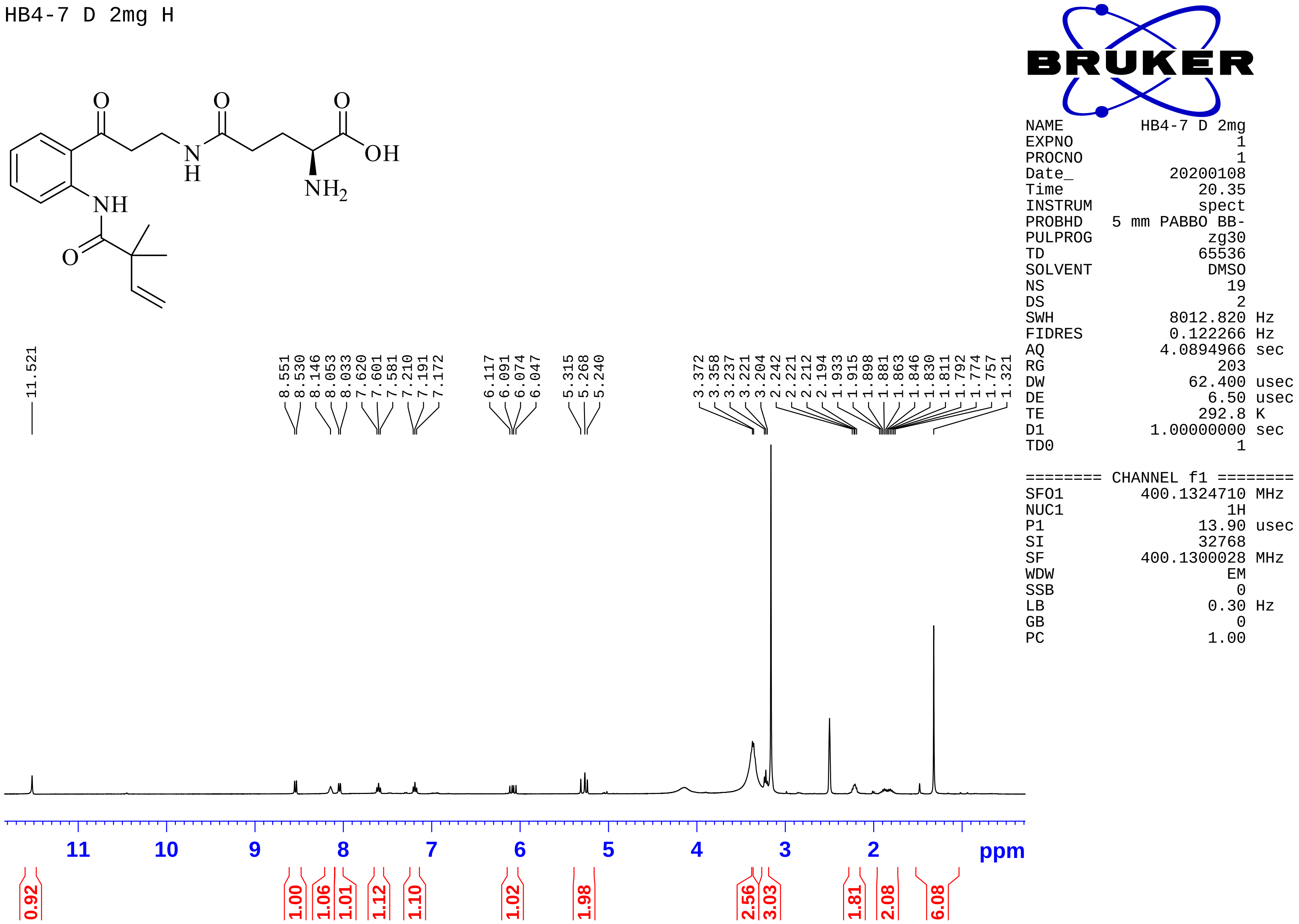

Figure S22. ^{1}H NMR spectrum of **4**.

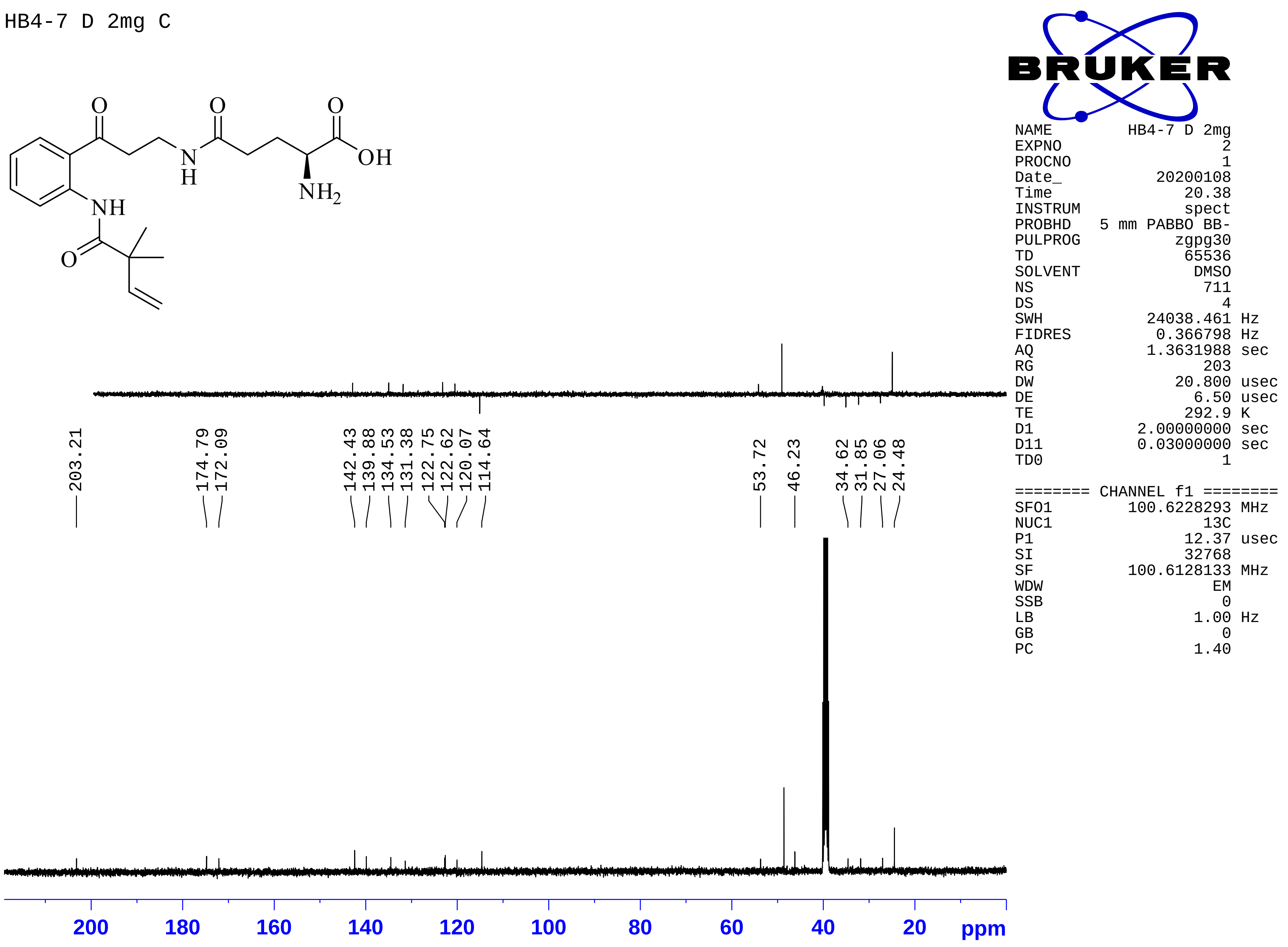

Figure S23. ^{13}C NMR spectrum of **4**.

HB2-13 C 5mg H

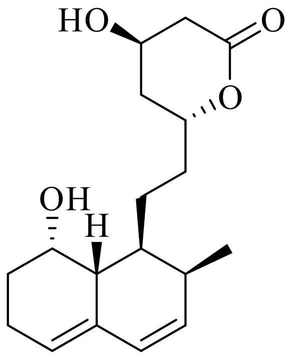

NAME	HB2-13 C 5mg
EXPNO	1
PROCNO	1
Date_	20200805
Time	18.50
INSTRUM	spect
PROBHD	5 mm PABBO BB-
PULPROG	zg30
TD	65536
SOLVENT	CDCl3
NS	21
DS	2
SWH	8012.820 Hz
FIDRES	0.122266 Hz
AQ	4.0894966 sec
RG	203
DW	62.400 usec
DE	6.50 usec
TE	296.3 K
D1	1.00000000 sec
TD0	1

======== CHANNEL f1 ========

SFO1	400.1324710 MHz
NUC1	1H
P1	13.90 usec
SI	32768
SF	400.1300000 MHz
WDW	EM
SSB	0
LB	0.30 Hz
GB	0
PC	1.00

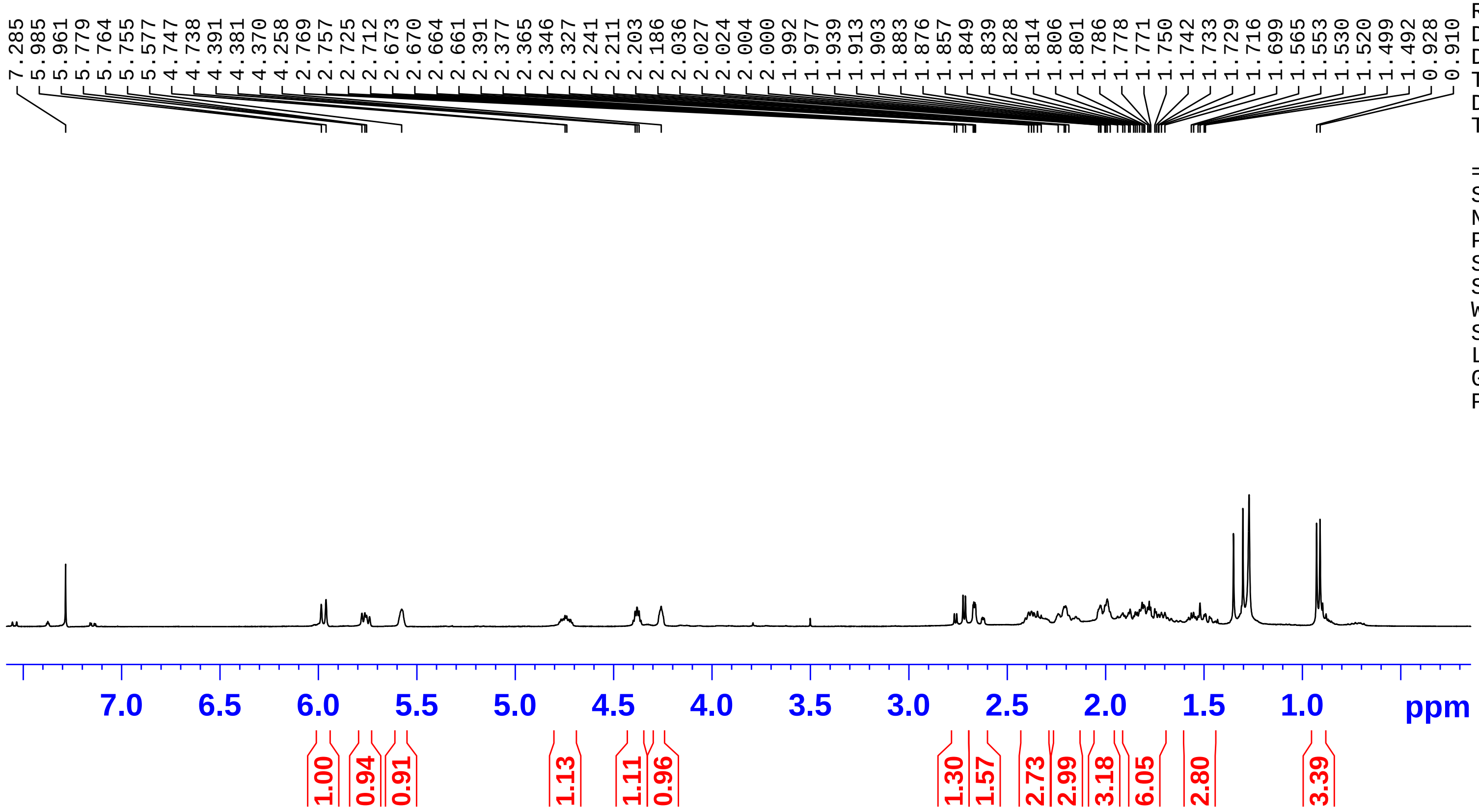

Figure S24. ^{1}H NMR spectrum of **5**.

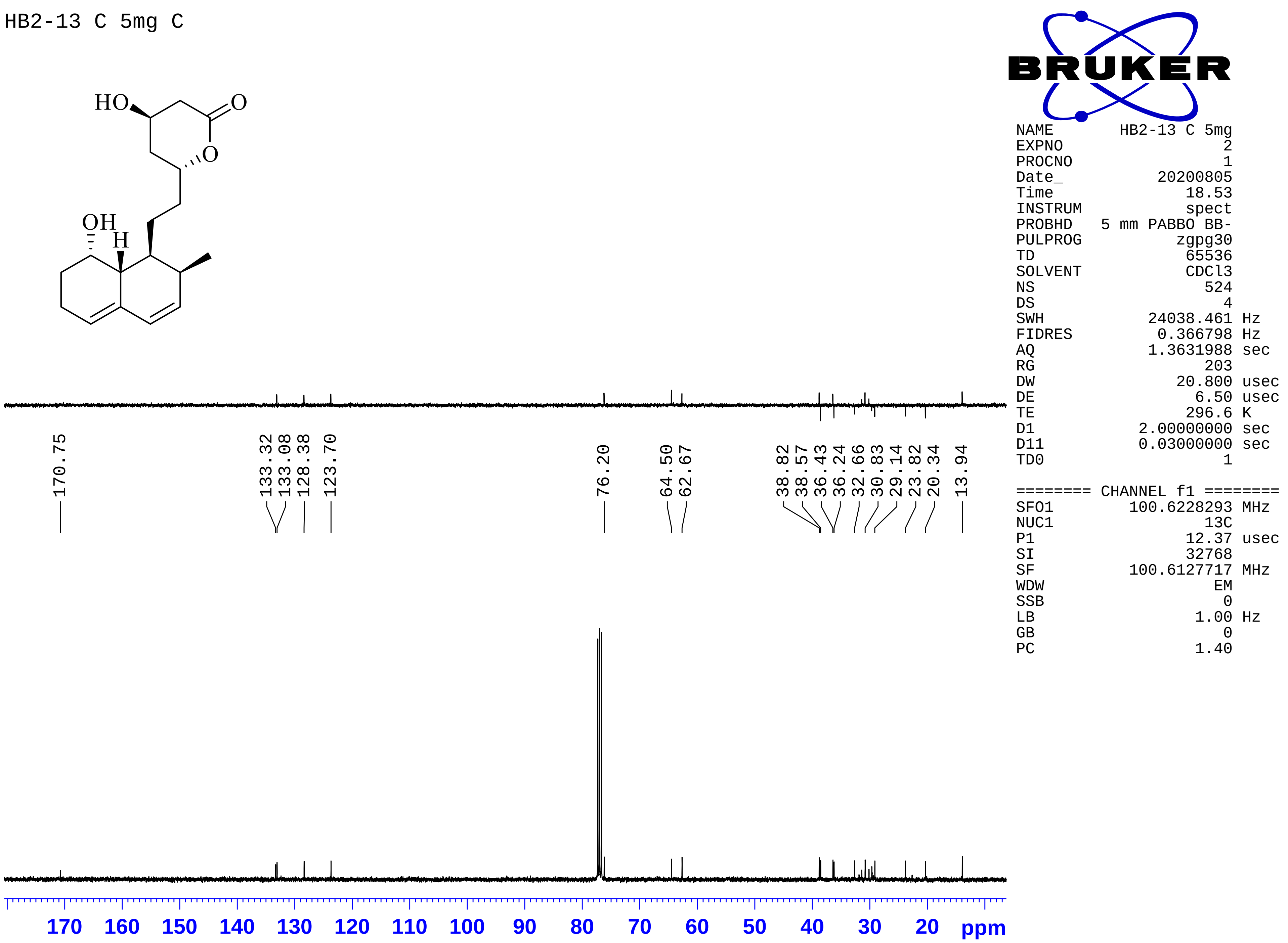

Figure S25. ^{13}C NMR spectrum of **5**.

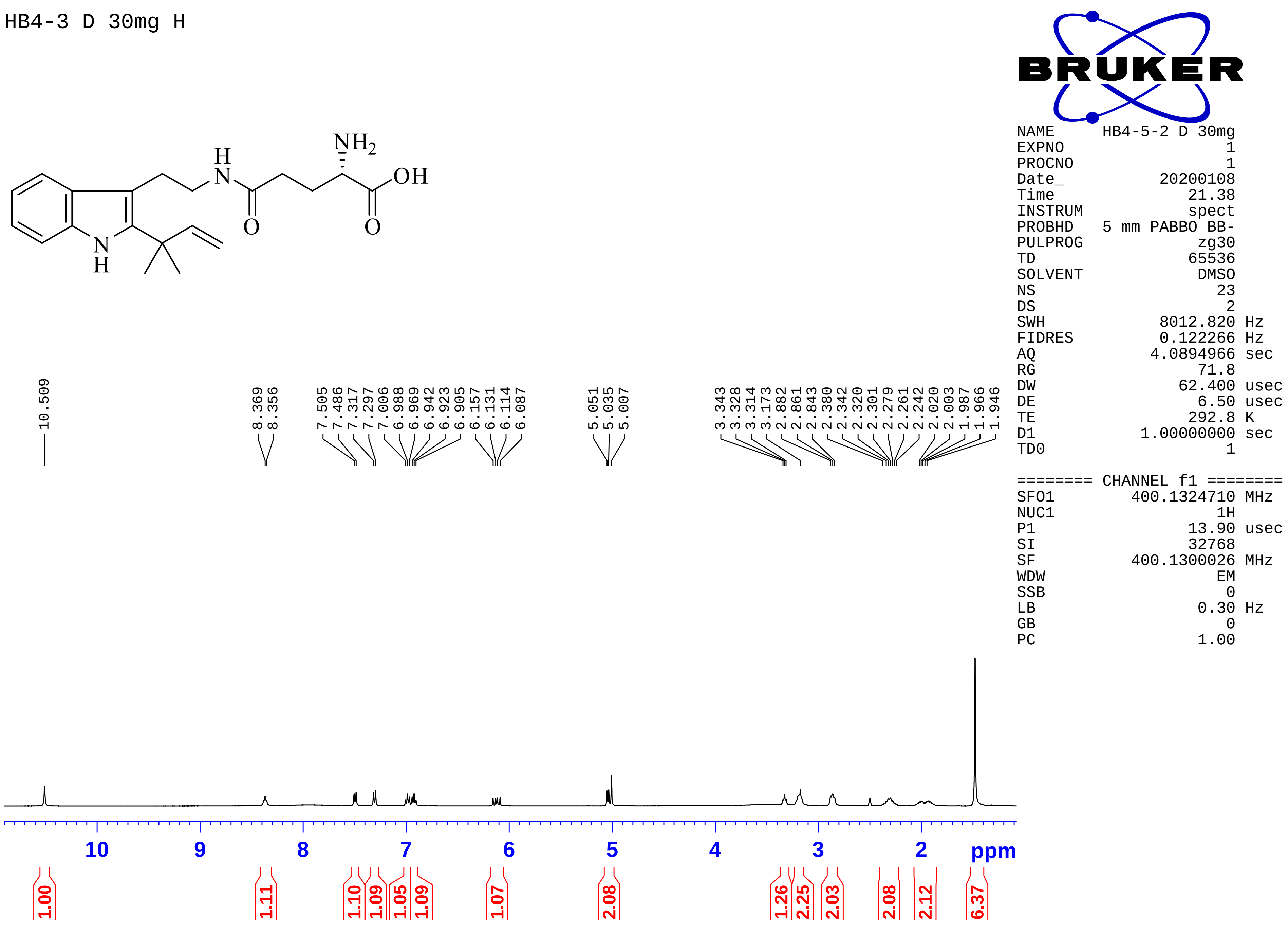

Figure S26. ^{1}H NMR spectrum of **6**.

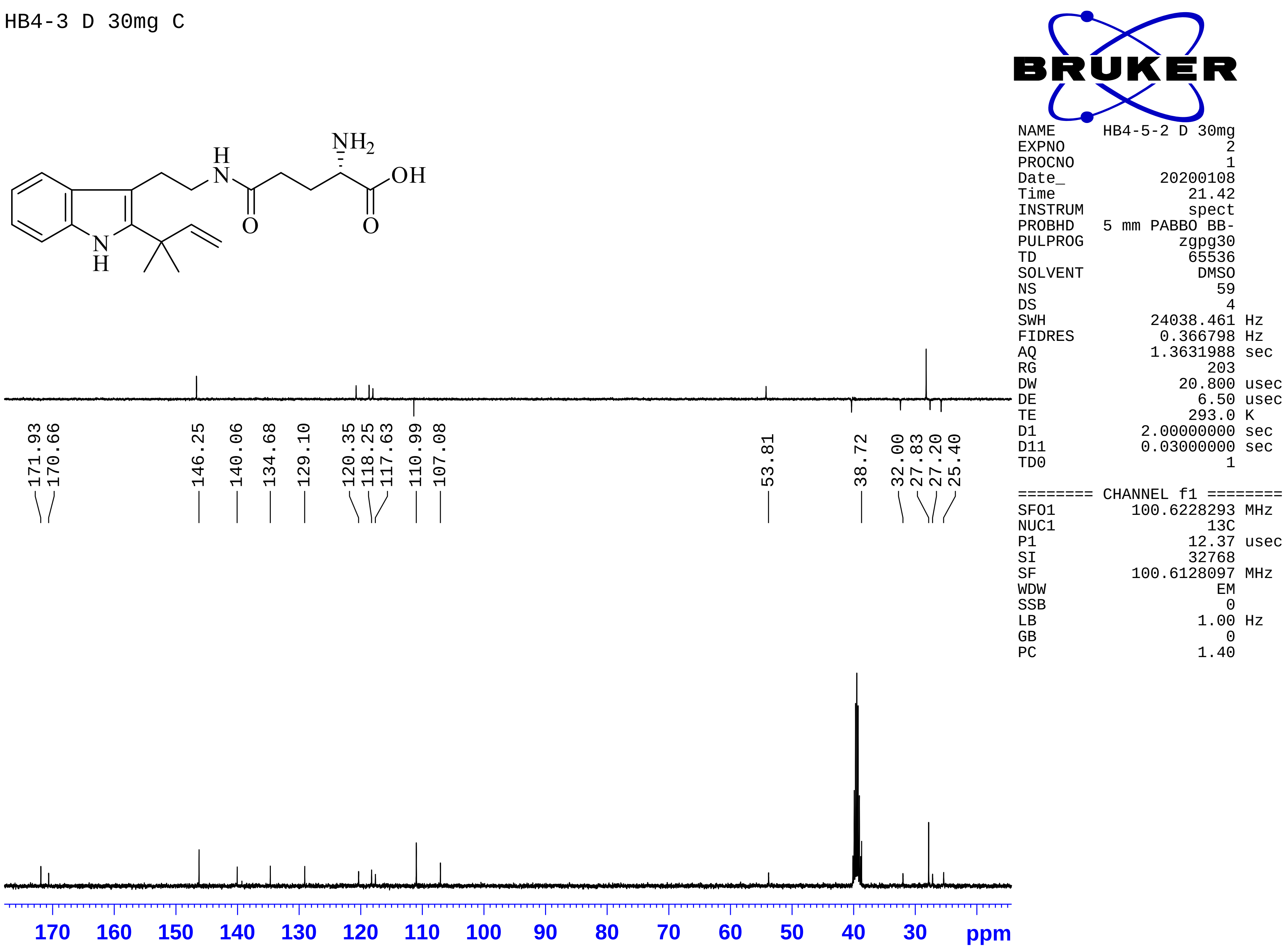

Figure S27. ^{13}C NMR spectrum of **6**.

HB2-7 C 12mg H

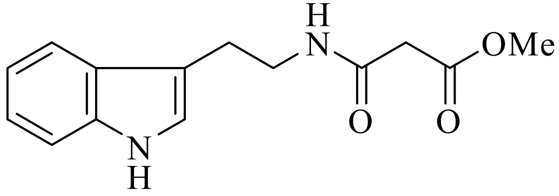

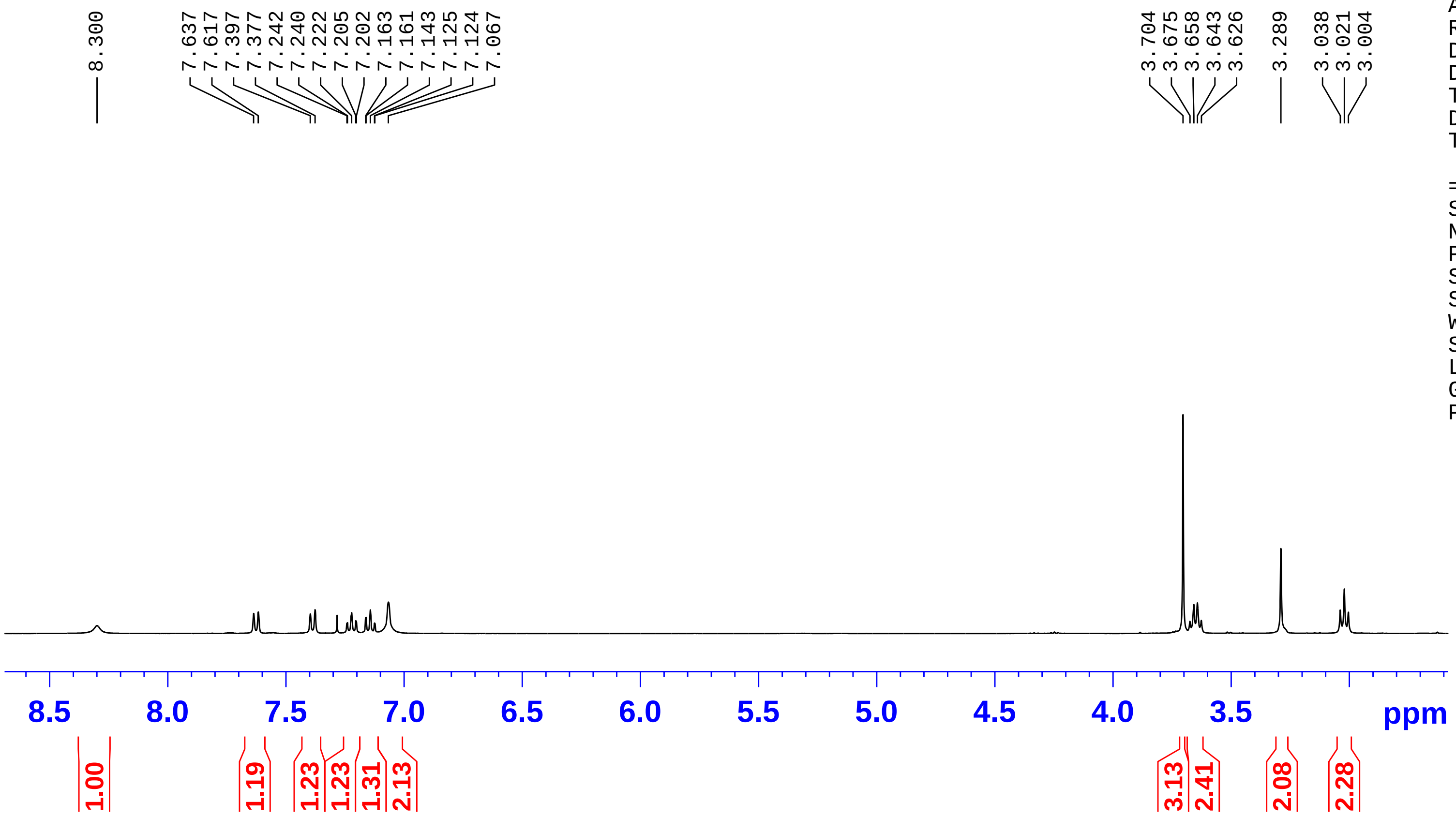

NAME	HB2-7 C 12mg	
EXPNO	1	
PROCNO	1	
Date_	20200801	
Time	9.01	
INSTRUM	spect	
PROBHD	5 mm PABBO BB-	
PULPROG	zg30	
TD	65536	
SOLVENT	CDCl3	
NS	18	
DS	2	
SWH	8012.820	Hz
FIDRES	0.122266	Hz
AQ	4.0894966	sec
RG	161	
DW	62.400	usec
DE	6.50	usec
TE	296.2	K
D1	1.00000000	sec
TD0	1	

======== CHANNEL f1 ========

SFO1	400.1324710	MHz
NUC1	1H	
P1	13.90	usec
SI	32768	
SF	400.1300000	MHz
WDW	EM	
SSB	0	
LB	0.30	Hz
GB	0	
PC	1.00	

Figure S28. ^{1}H NMR spectrum of **7**.

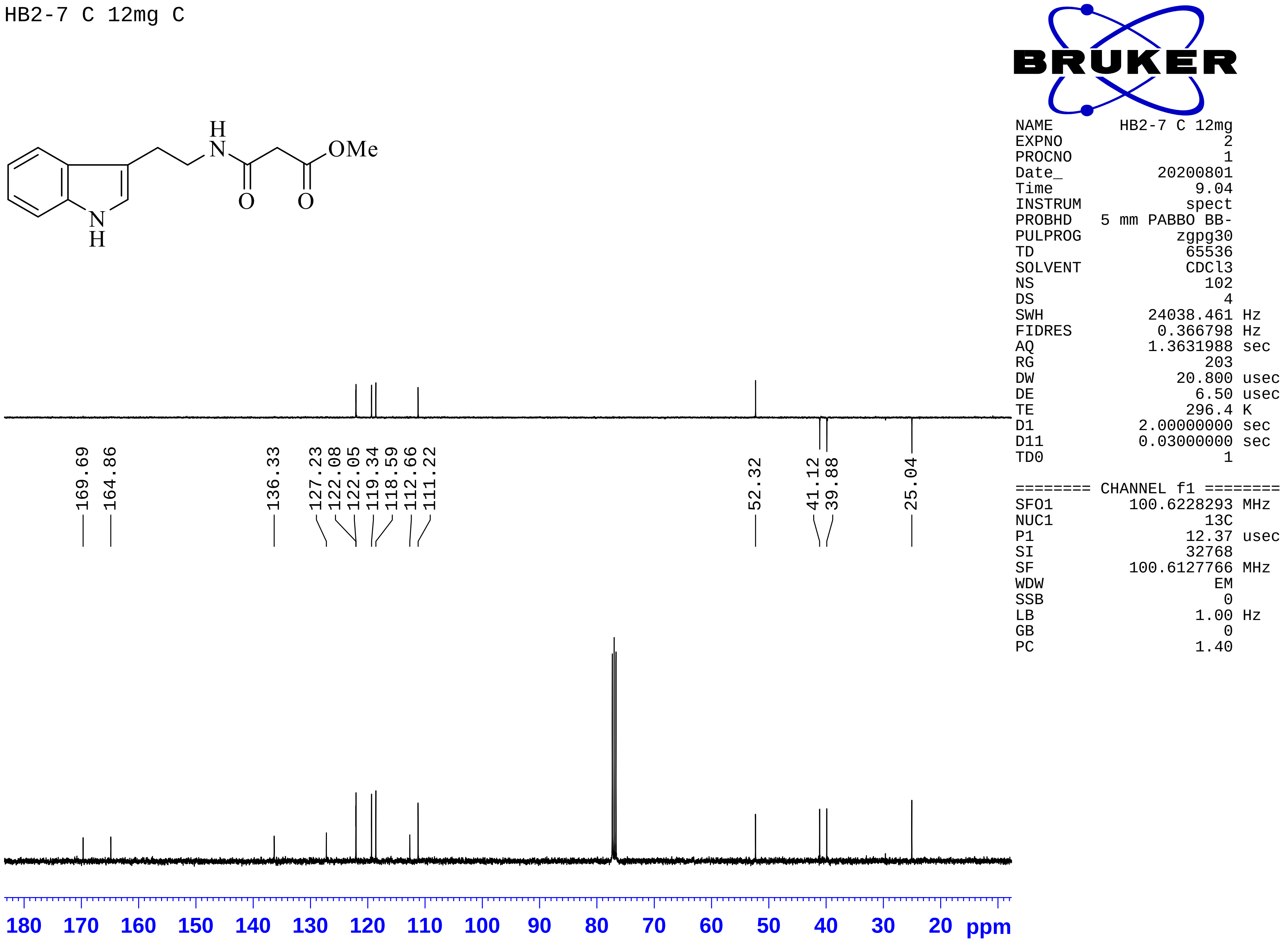

Figure S29. ^{13}C NMR spectrum of **7**.

HB5-6 M 10mg H

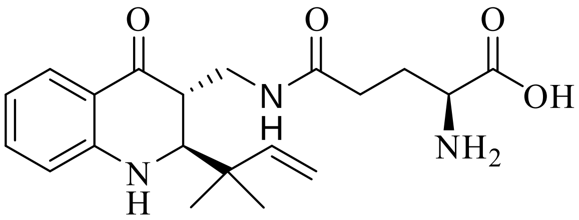

NAME	HB5-6 M 10mg
EXPNO	1
PROCNO	1
Date_	20200712
Time	13.46
INSTRUM	spect
PROBHD	5 mm PABBO BB-
PULPROG	zg30
TD	65536
SOLVENT	MeOD
NS	20
DS	2
SWH	8012.820 Hz
FIDRES	0.122266 Hz
AQ	4.0894966 sec
RG	144
DW	62.400 usec
DE	6.50 usec
TE	299.6 K
D1	1.00000000 sec
TD0	1

======== CHANNEL f1 ========

SFO1	400.1324710 MHz
NUC1	1H
P1	13.90 usec
SI	32768
SF	400.1300117 MHz
WDW	EM
SSB	0
LB	0.30 Hz
GB	0
PC	1.00

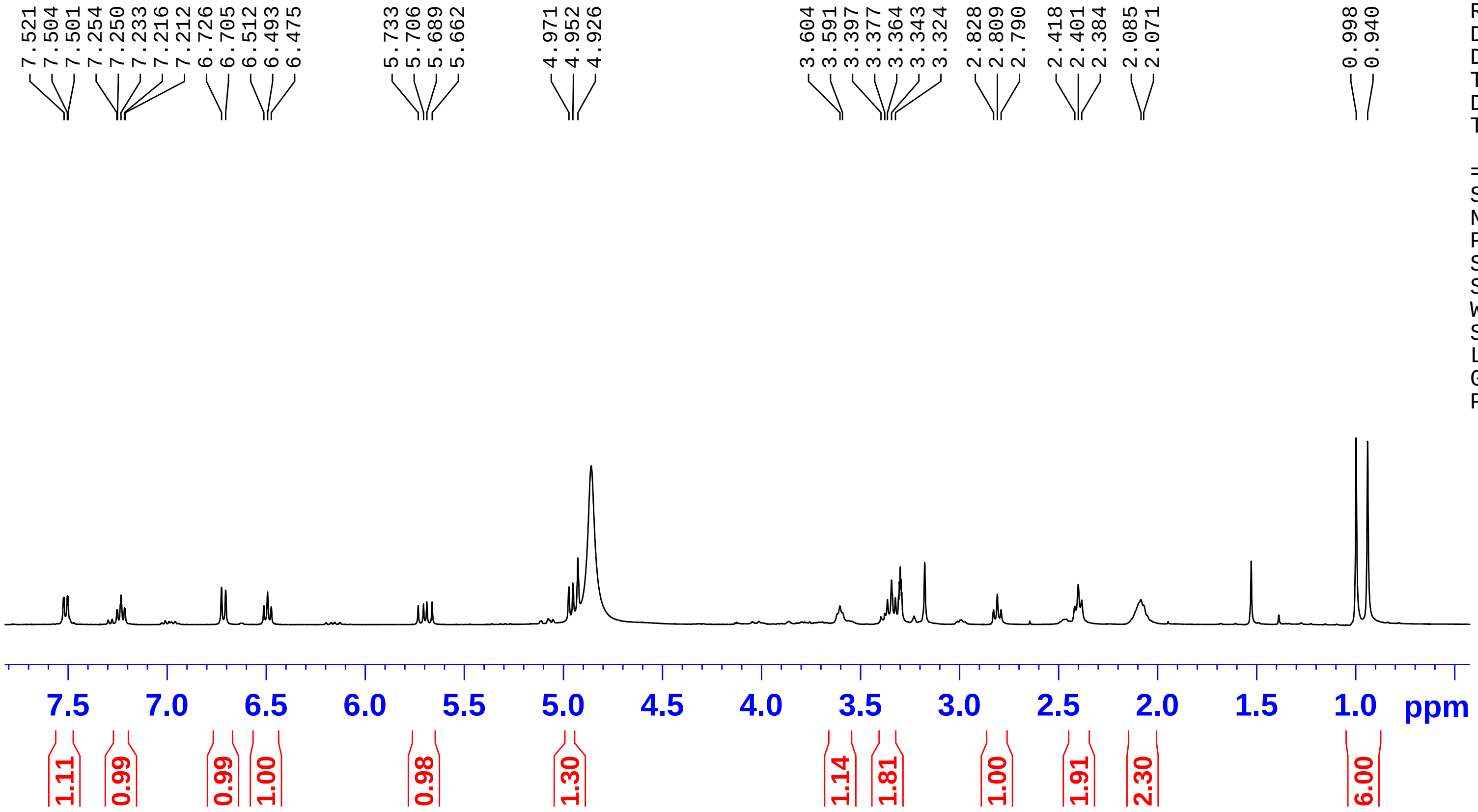

Figure S30. ^{1}H NMR spectrum of **8**.

HB5-6 M 10mg C

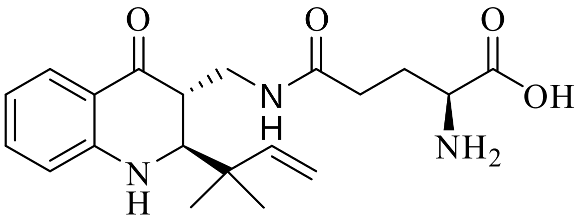

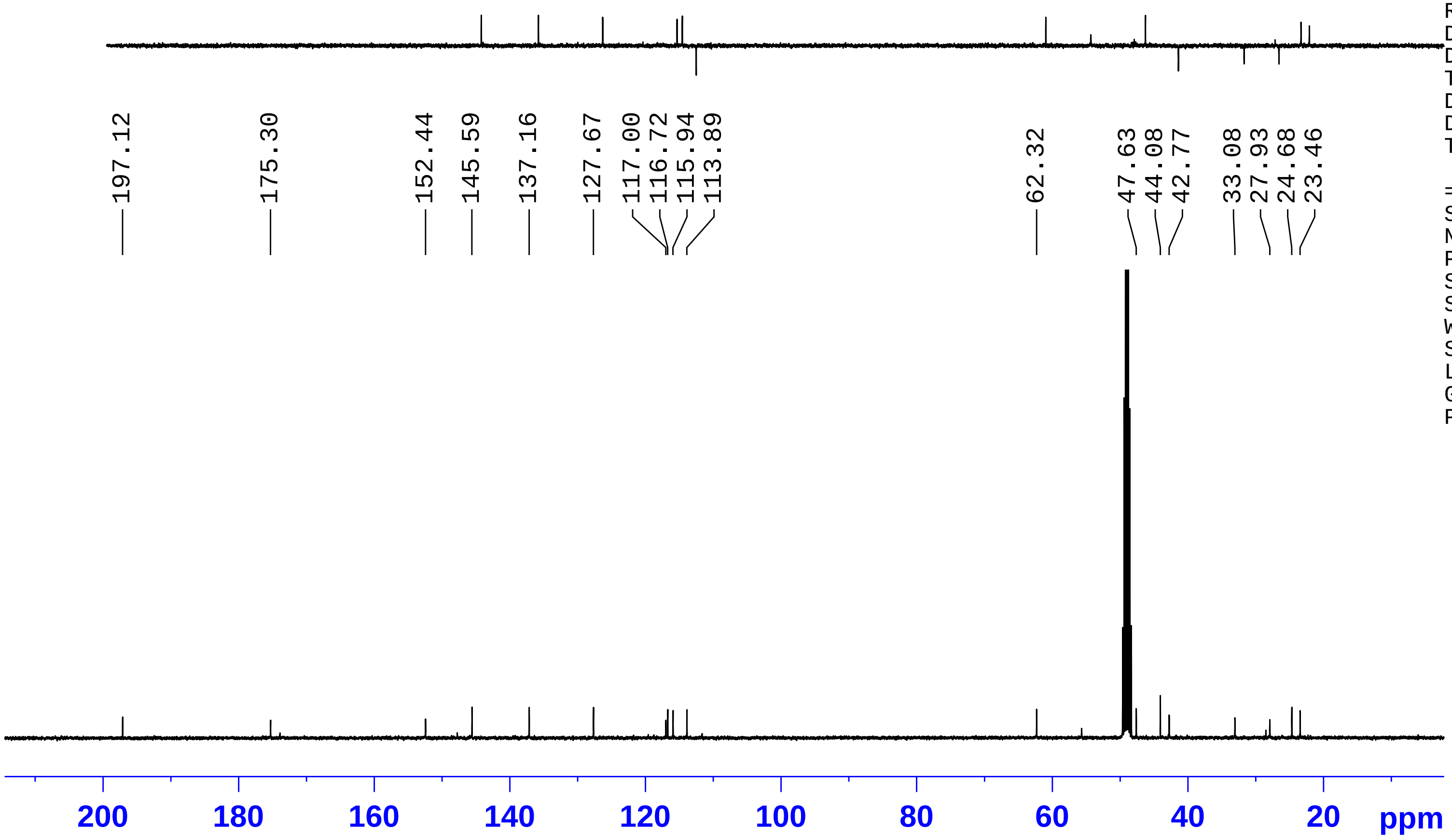

NAME	HB5-6 M 10mg	
EXPNO	2	
PROCNO	1	
Date_	20200712	
Time	13.48	
INSTRUM	spect	
PROBHD	5 mm PABBO BB-	
PULPROG	zgpg30	
TD	65536	
SOLVENT	MeOD	
NS	424	
DS	4	
SWH	24038.461	Hz
FIDRES	0.366798	Hz
AQ	1.3631988	sec
RG	203	
DW	20.800	usec
DE	6.50	usec
TE	299.7	K
D1	2.00000000	sec
D11	0.03000000	sec
TD0	1	

======== CHANNEL f1 ========

SFO1	100.6228293	MHz
NUC1	13C	
P1	12.37	usec
SI	32768	
SF	100.6126303	MHz
WDW	EM	
SSB	0	
LB	1.00	Hz
GB	0	
PC	1.40	

Figure S31. ^{13}C NMR spectrum of **8**.

HB2-8 C 2mg H

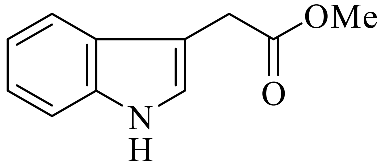

NAME	HB2-8 C 2mg
EXPNO	1
PROCNO	1
Date_	20200801
Time	10.41
INSTRUM	spect
PROBHD	5 mm PABBO BB-
PULPROG	zg30
TD	65536
SOLVENT	CDCl3
NS	15
DS	2
SWH	8012.820 Hz
FIDRES	0.122266 Hz
AQ	4.0894966 sec
RG	203
DW	62.400 usec
DE	6.50 usec
TE	296.2 K
D1	1.00000000 sec
TD0	1

======== CHANNEL f1 ========

SFO1	400.1324710 MHz
NUC1	1H
P1	13.90 usec
SI	32768
SF	400.1300000 MHz
WDW	EM
SSB	0
LB	0.30 Hz
GB	0
PC	1.00

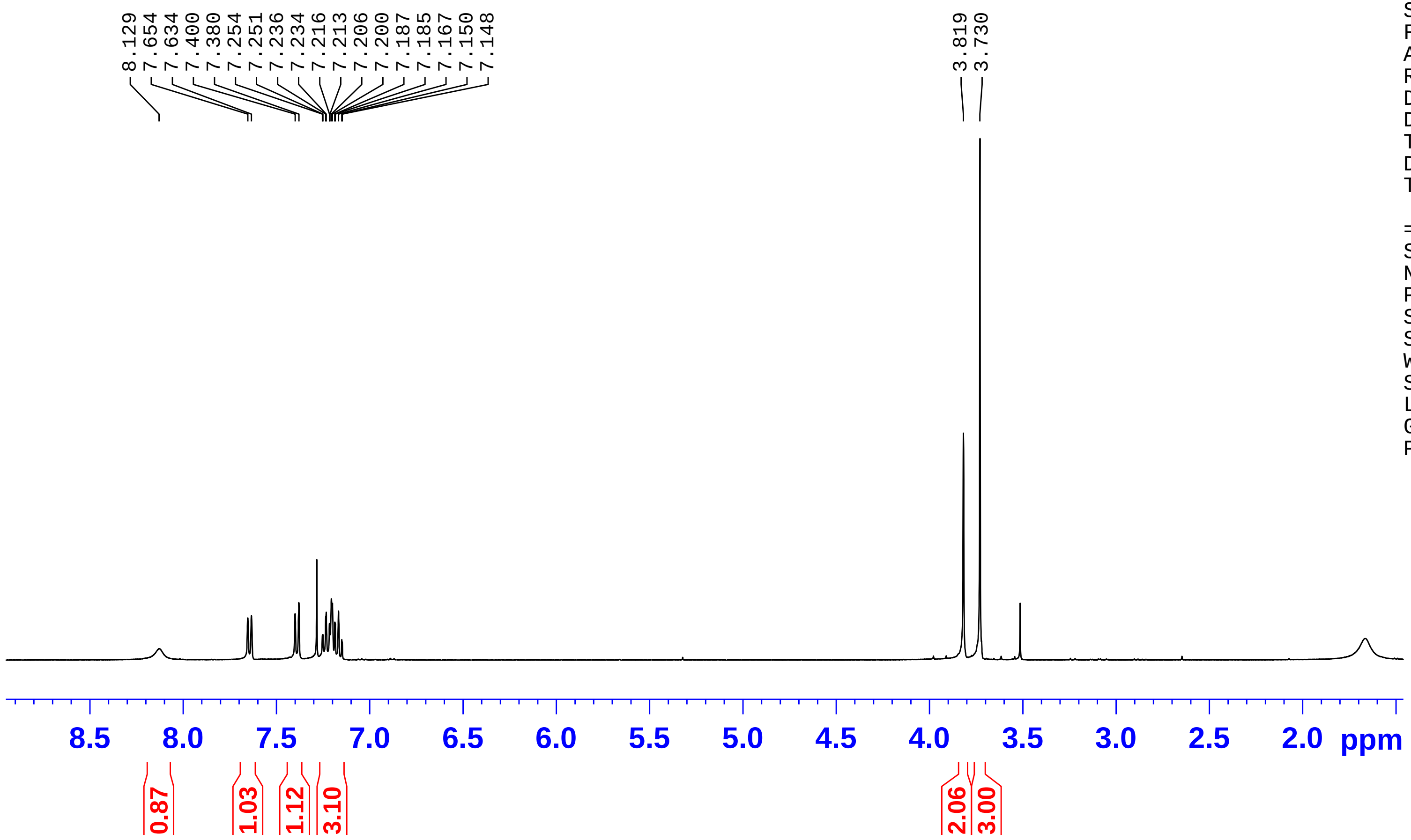

Figure S32. ^{1}H NMR spectrum of **9**.

HB2-8 C 2mg C

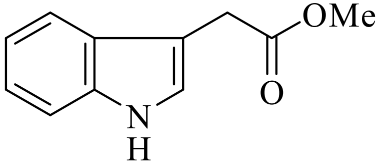

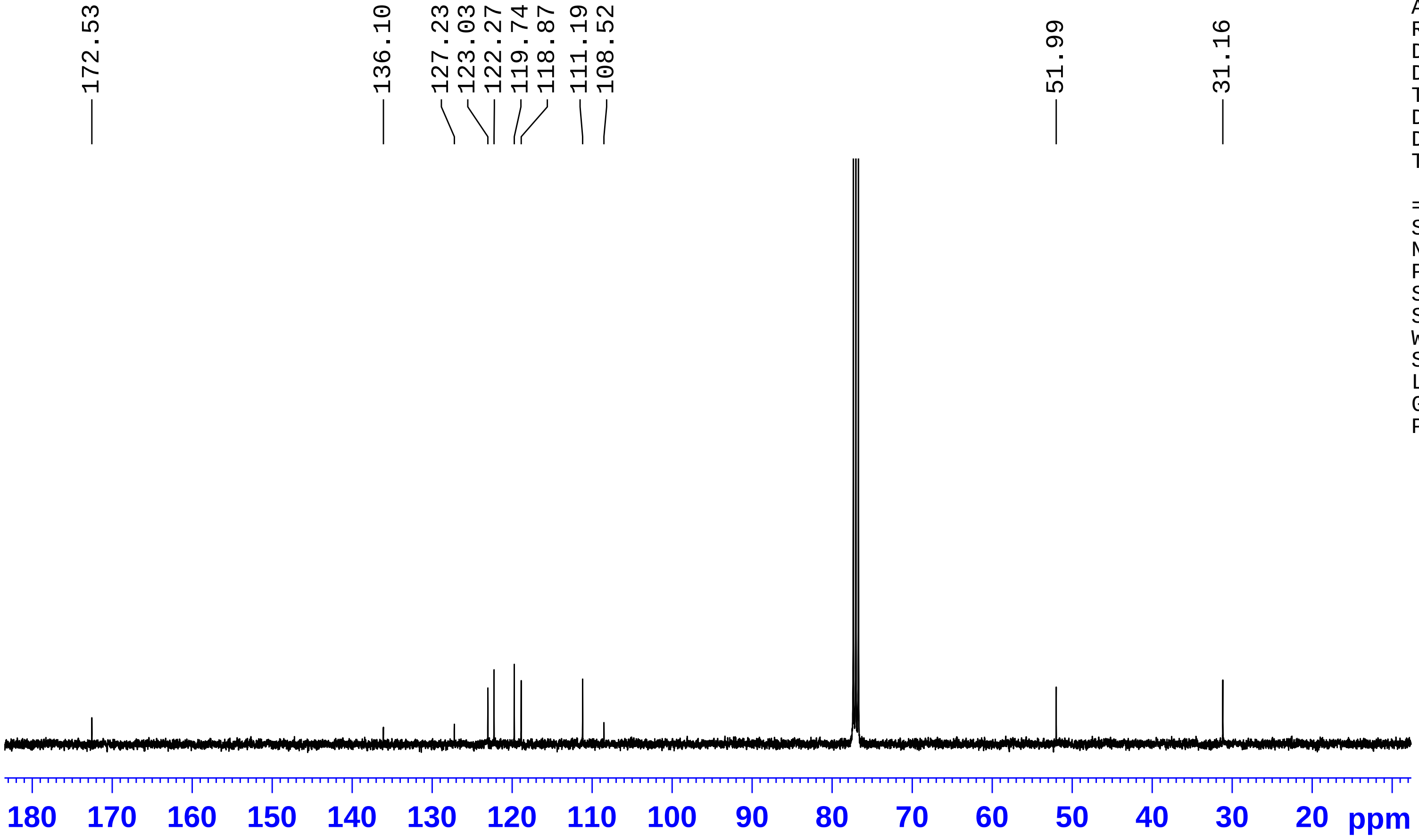

NAME	HB2-8 C 2mg
EXPNO	2
PROCNO	1
Date_	20200801
Time	10.43
INSTRUM	spect
PROBHD	5 mm PABBO BB-
PULPROG	zgpg30
TD	65536
SOLVENT	CDCl3
NS	530
DS	4
SWH	24038.461 Hz
FIDRES	0.366798 Hz
AQ	1.3631988 sec
RG	203
DW	20.800 usec
DE	6.50 usec
TE	296.4 K
D1	2.00000000 sec
D11	0.03000000 sec
TD0	1

======== CHANNEL f1 ========

SFO1	100.6228293 MHz
NUC1	13C
P1	12.37 usec
SI	32768
SF	100.6127685 MHz
WDW	EM
SSB	0
LB	1.00 Hz
GB	0
PC	1.40

Figure S33. ^{13}C NMR spectrum of **9**.

HB-C14 M 127mg H

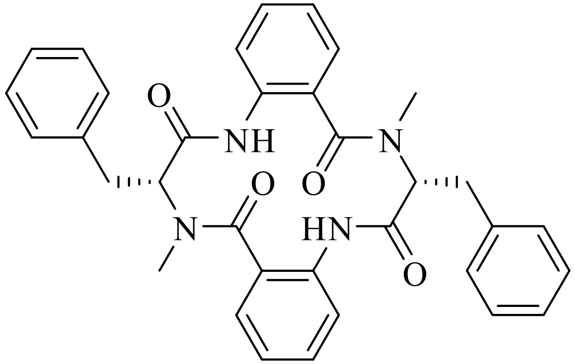

NAME	HB-C14 M 127mg
EXPNO	1
PROCNO	1
Date_	20200910
Time	20.24
INSTRUM	spect
PROBHD	5 mm PABBO BB-
PULPROG	zg30
TD	65536
SOLVENT	MeOD
NS	10
DS	2
SWH	8012.820 Hz
FIDRES	0.122266 Hz
AQ	4.0894966 sec
RG	32
DW	62.400 usec
DE	6.50 usec
TE	296.4 K
D1	1.00000000 sec
TD0	1

======== CHANNEL f1 ========

SFO1	400.1324710 MHz
NUC1	1H
P1	13.90 usec
SI	32768
SF	400.1300113 MHz
WDW	EM
SSB	0
LB	0.30 Hz
GB	0
PC	1.00

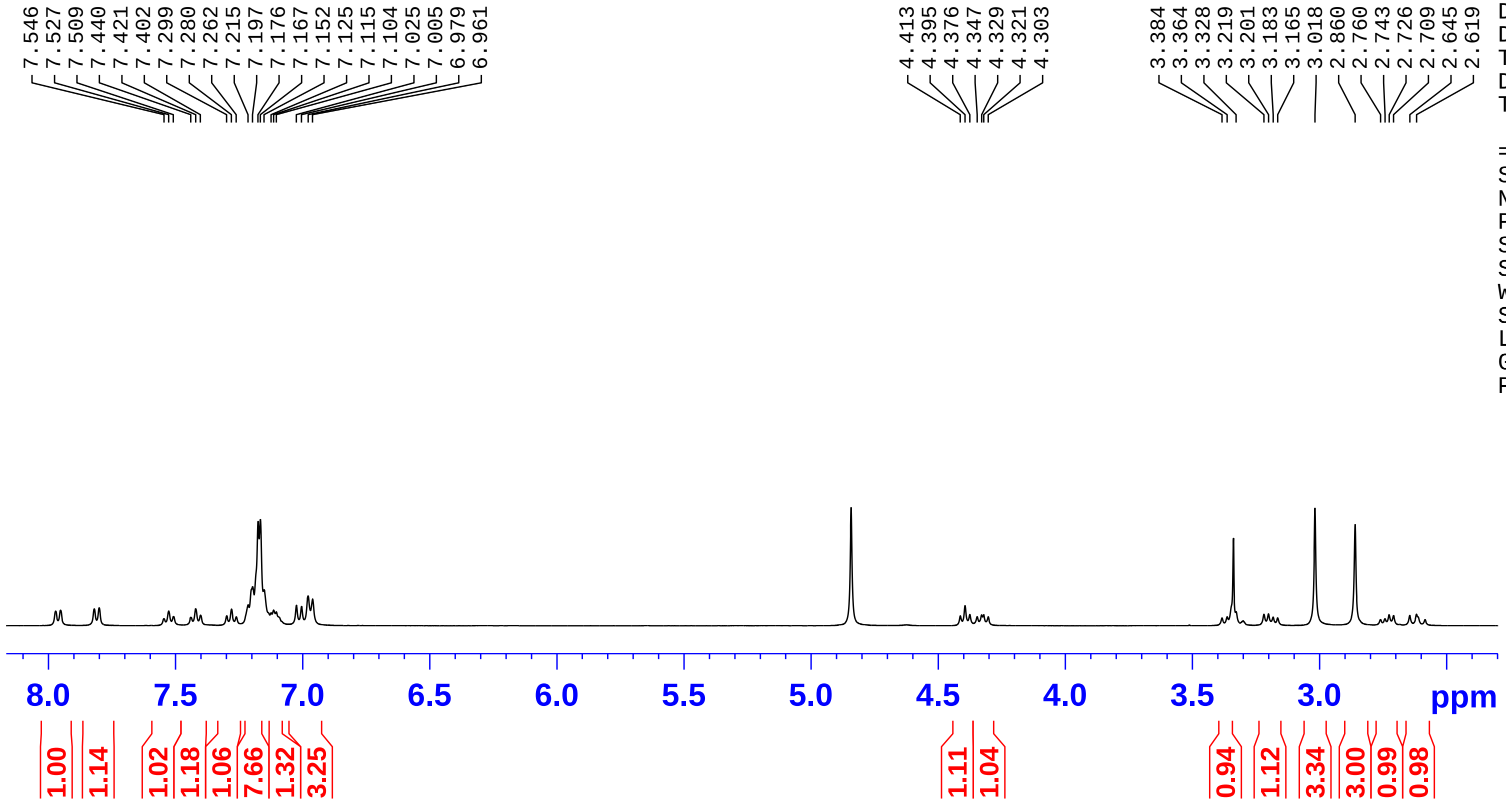

Figure S34. ^{1}H NMR spectrum of **10**.

HB-C14 M 127mg C

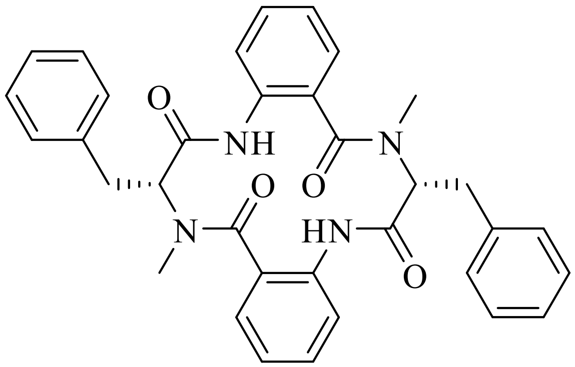

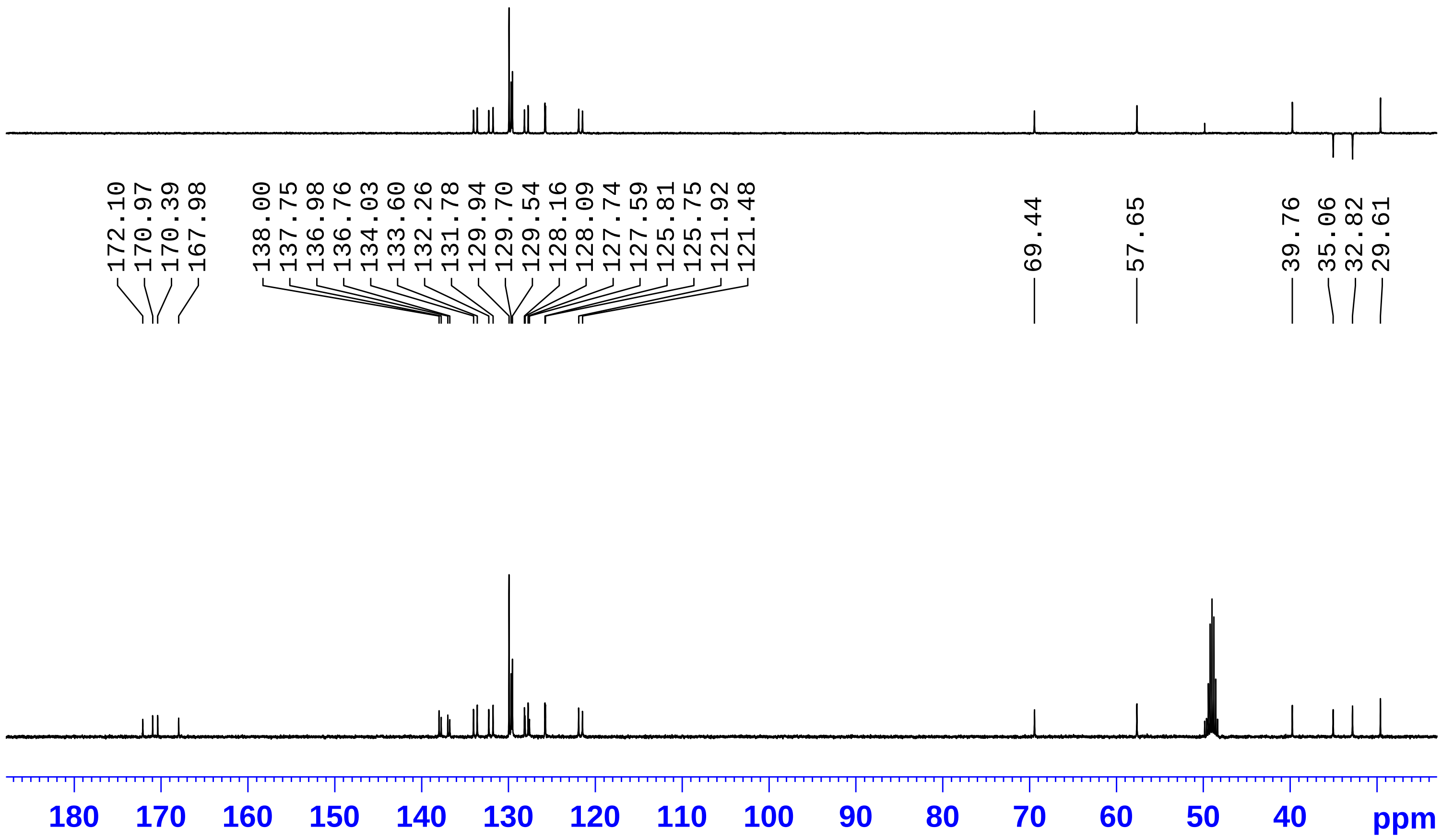

NAME	HB-C14 M 127mg
EXPNO	2
PROCNO	1
Date_	20200910
Time	20.27
INSTRUM	spect
PROBHD	5 mm PABBO BB-
PULPROG	zgpg30
TD	65536
SOLVENT	MeOD
NS	46
DS	4
SWH	24038.461 Hz
FIDRES	0.366798 Hz
AQ	1.3631988 sec
RG	203
DW	20.800 usec
DE	6.50 usec
TE	296.9 K
D1	2.00000000 sec
D11	0.03000000 sec
TD0	1

======== CHANNEL f1 ========

SFO1	100.6228293 MHz
NUC1	13C
P1	12.37 usec
SI	32768
SF	100.6126497 MHz
WDW	EM
SSB	0
LB	1.00 Hz
GB	0
PC	1.40

Figure S35. ^{13}C NMR spectrum of **10**.

HB-C8 M 2mg H

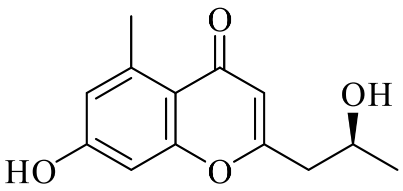

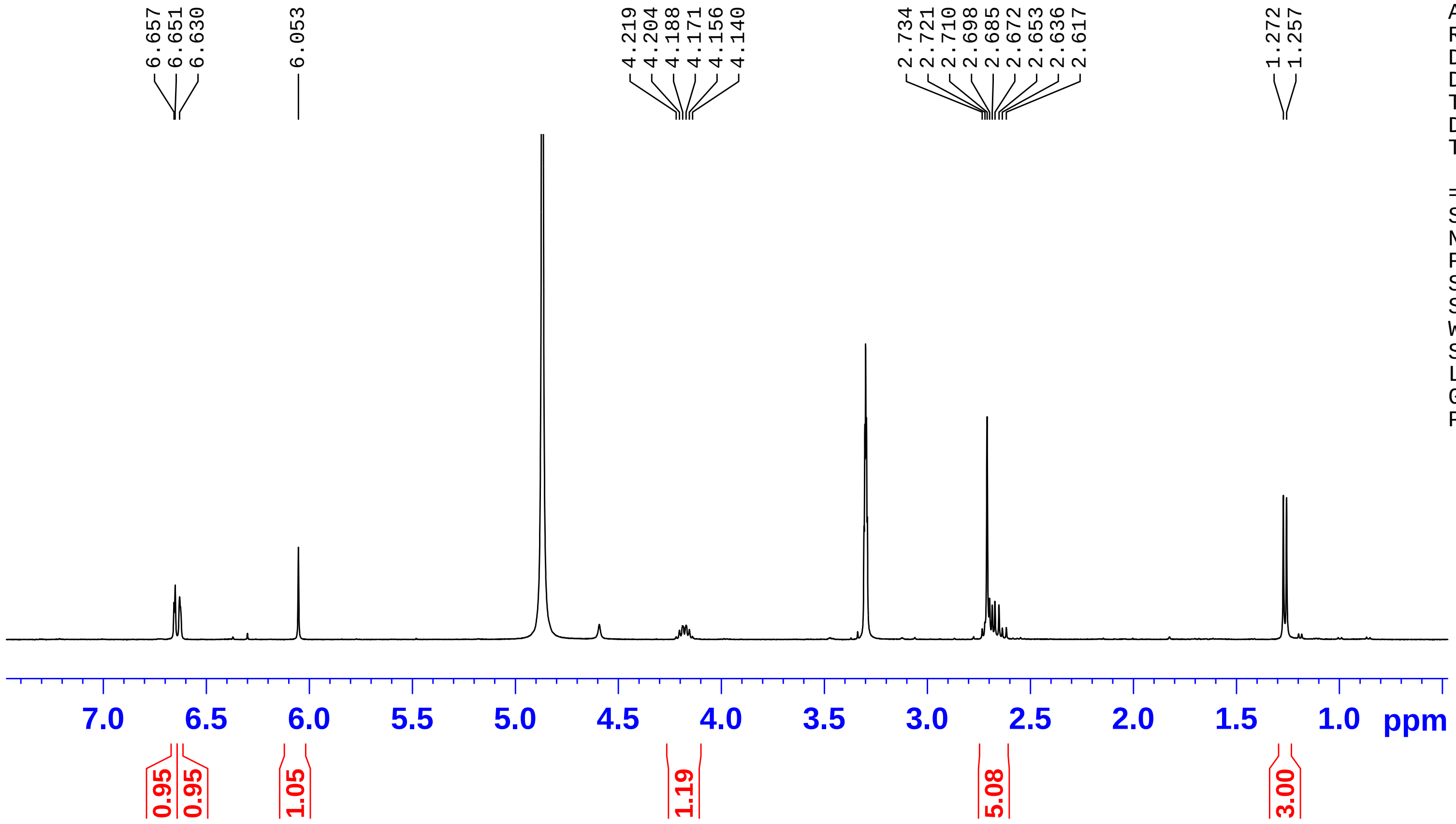

NAME	HB-C8 M 2mg
EXPNO	1
PROCNO	1
Date_	20200903
Time	20.56
INSTRUM	spect
PROBHD	5 mm PABBO BB-
PULPROG	zg30
TD	65536
SOLVENT	MeOD
NS	20
DS	2
SWH	8012.820 Hz
FIDRES	0.122266 Hz
AQ	4.0894966 sec
RG	203
DW	62.400 usec
DE	6.50 usec
TE	296.6 K
D1	1.00000000 sec
TD0	1

======== CHANNEL f1 ========

SFO1	400.1324710 MHz
NUC1	1H
P1	13.90 usec
SI	32768
SF	400.1300117 MHz
WDW	EM
SSB	0
LB	0.30 Hz
GB	0
PC	1.00

Figure S36. ^{1}H NMR spectrum of **11**.

HB-C8 M 2mg C

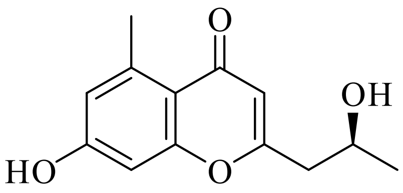

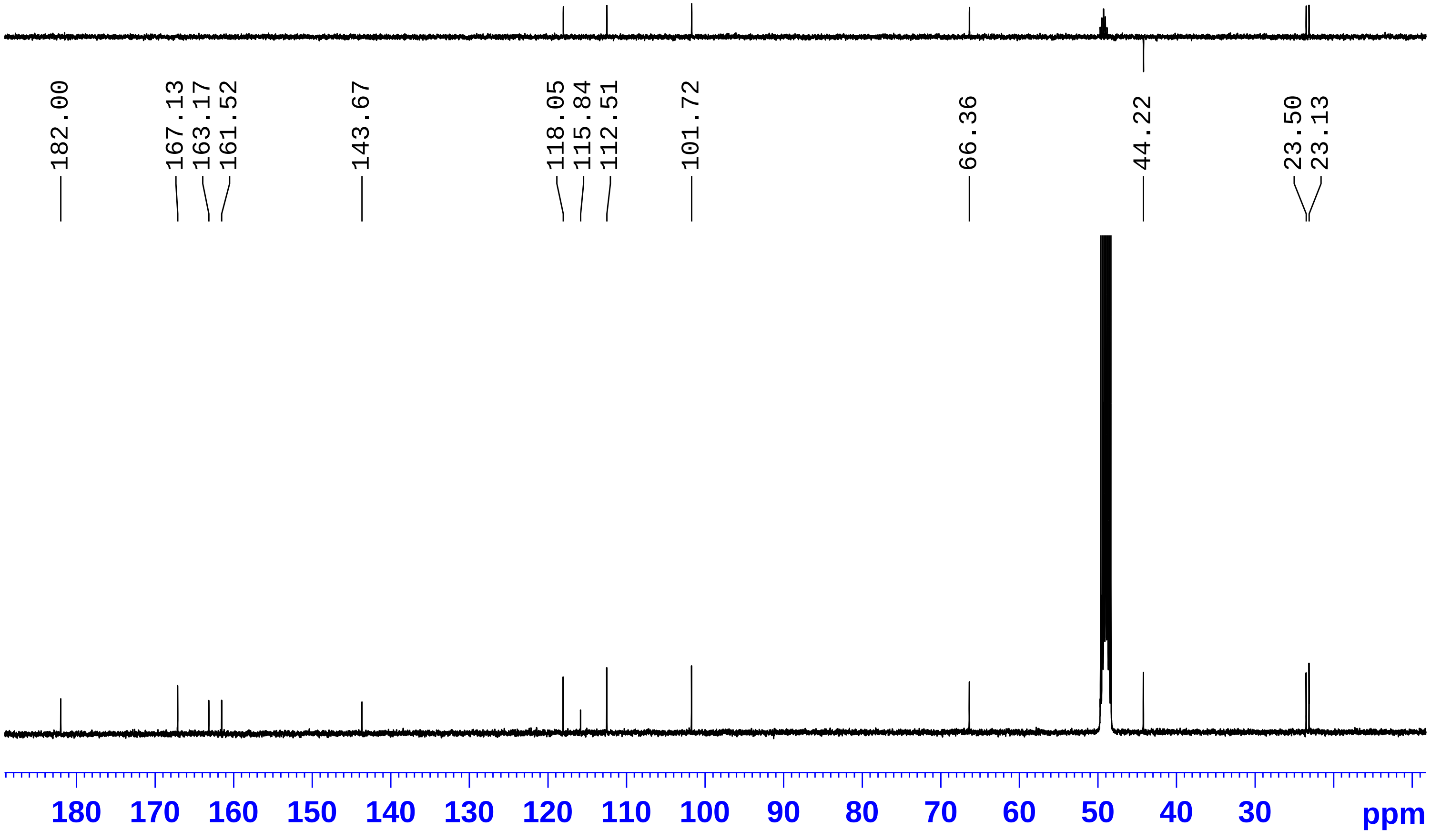

NAME	HB-C8 M 2mg	
EXPNO	2	
PROCNO	1	
Date_	20200903	
Time	21.00	
INSTRUM	spect	
PROBHD	5 mm PABBO BB-	
PULPROG	zgpg30	
TD	65536	
SOLVENT	MeOD	
NS	10240	
DS	4	
SWH	24038.461	Hz
FIDRES	0.366798	Hz
AQ	1.3631988	sec
RG	203	
DW	20.800	usec
DE	6.50	usec
TE	296.7	K
D1	2.00000000	sec
D11	0.03000000	sec
TD0	1	

======== CHANNEL f1 ========		
SFO1	100.6228293	MHz
NUC1	13C	
P1	12.37	usec
SI	32768	
SF	100.6126266	MHz
WDW	EM	
SSB	0	
LB	1.00	Hz
GB	0	
PC	1.40	

Figure S37. ^{13}C NMR spectrum of **11**.

HB2-3 M 2mg H

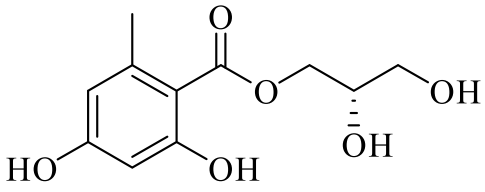

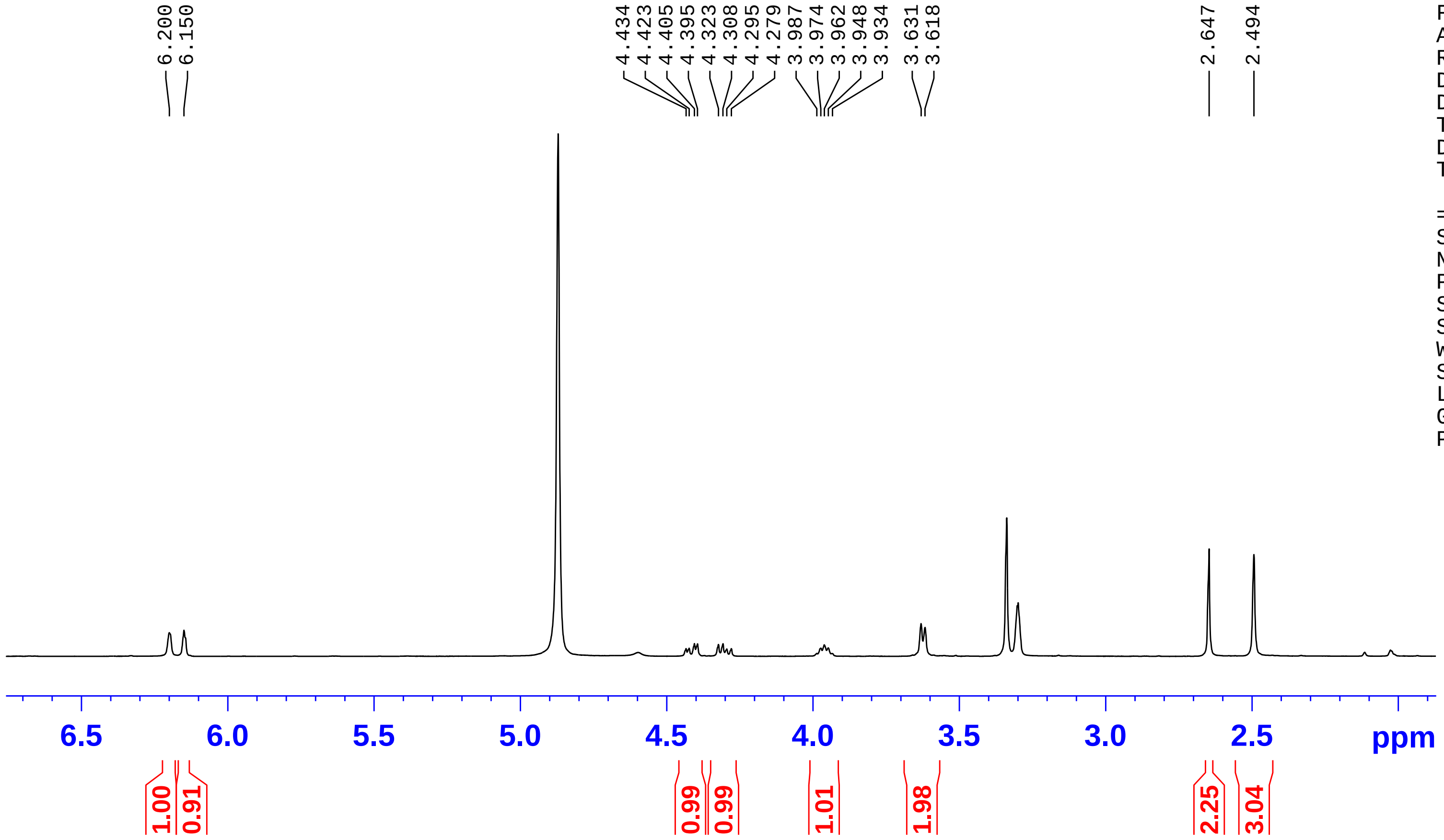

NAME	HB2-3 M 2mg
EXPNO	1
PROCNO	1
Date_	20200726
Time	17.36
INSTRUM	spect
PROBHD	5 mm PABBO BB-
PULPROG	zg30
TD	65536
SOLVENT	MeOD
NS	24
DS	2
SWH	8012.820 Hz
FIDRES	0.122266 Hz
AQ	4.0894966 sec
RG	203
DW	62.400 usec
DE	6.50 usec
TE	296.2 K
D1	1.00000000 sec
TD0	1

======== CHANNEL f1 ========

SFO1	400.1324710 MHz
NUC1	1H
P1	13.90 usec
SI	32768
SF	400.1300116 MHz
WDW	EM
SSB	0
LB	0.30 Hz
GB	0
PC	1.00

Figure S38. ^{1}H NMR spectrum of **12**.

HB2-3 M 2mg C

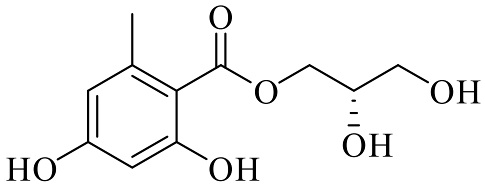

NAME	HB2-3 M 2mg
EXPNO	2
PROCNO	1
Date_	20200726
Time	17.38
INSTRUM	spect
PROBHD	5 mm PABBO BB-
PULPROG	zgpg30
TD	65536
SOLVENT	MeOD
NS	2767
DS	4
SWH	24038.461 Hz
FIDRES	0.366798 Hz
AQ	1.3631988 sec
RG	203
DW	20.800 usec
DE	6.50 usec
TE	296.4 K
D1	2.00000000 sec
D11	0.03000000 sec
TD0	1

======== CHANNEL f1 ========

SFO1	100.6228293 MHz
NUC1	13C
P1	12.37 usec
SI	32768
SF	100.6126282 MHz
WDW	EM
SSB	0
LB	1.00 Hz
GB	0
PC	1.40

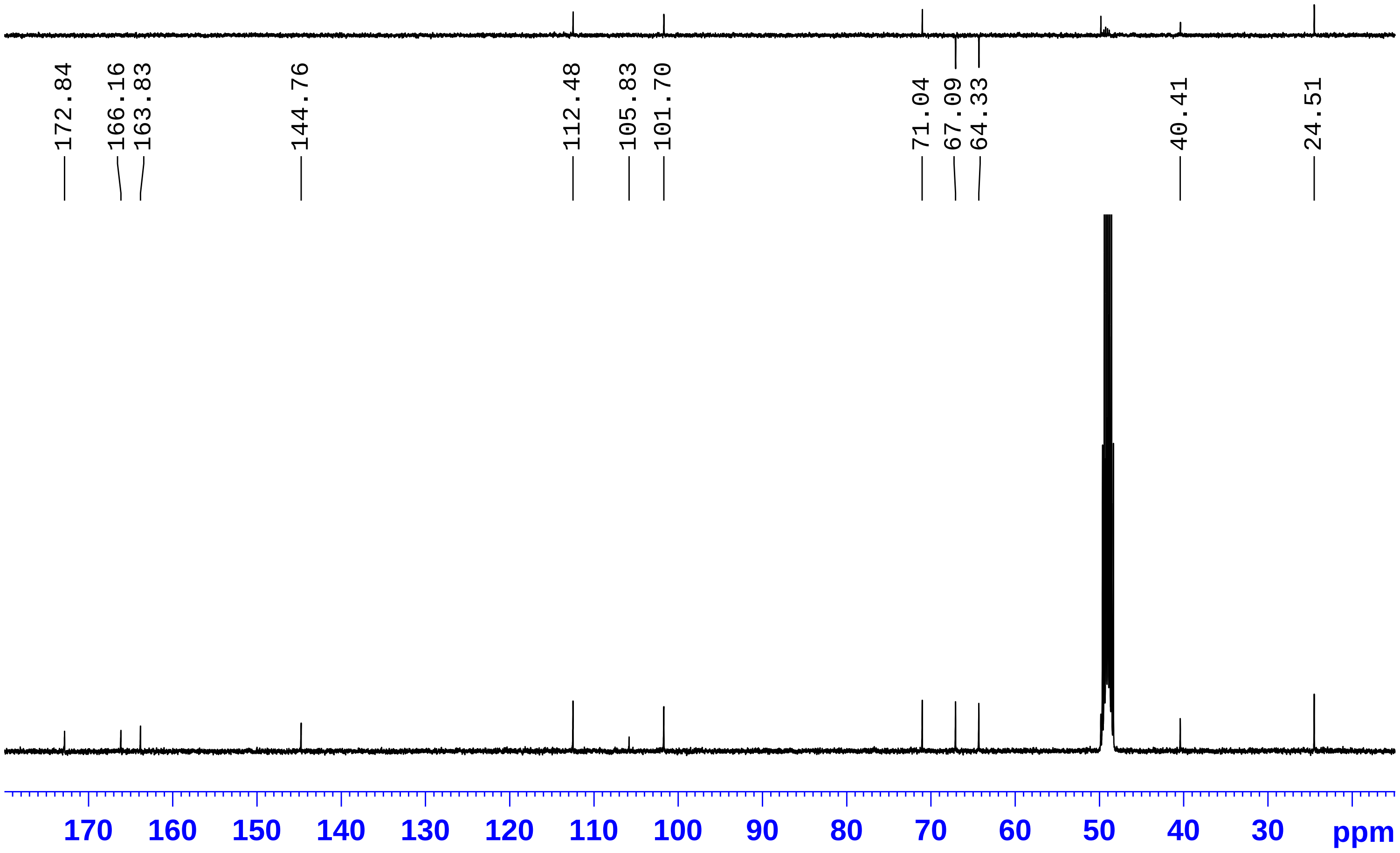

Figure S39. ^{13}C NMR spectrum of **12**.

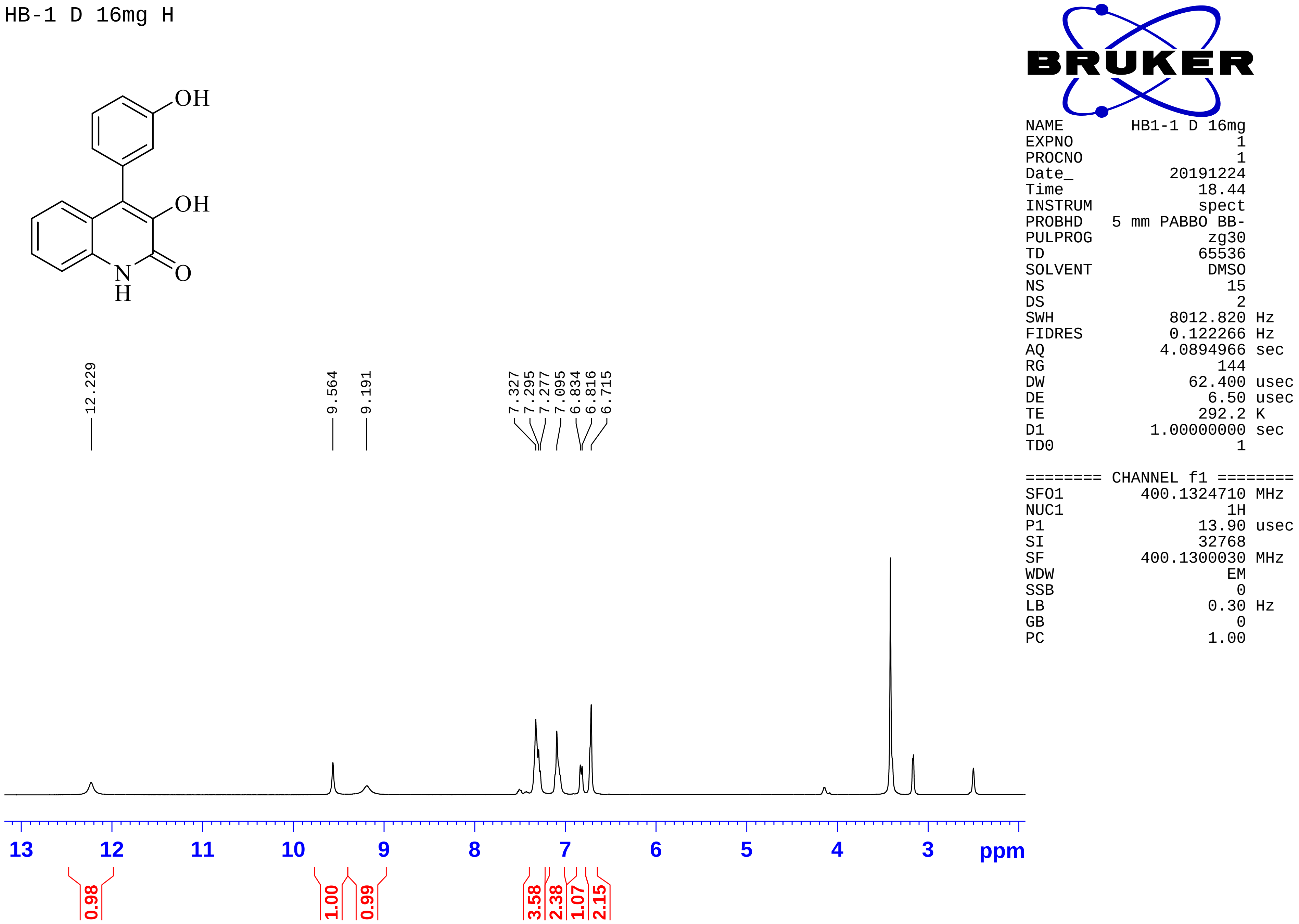

Figure S40. ^{1}H NMR spectrum of **13**.

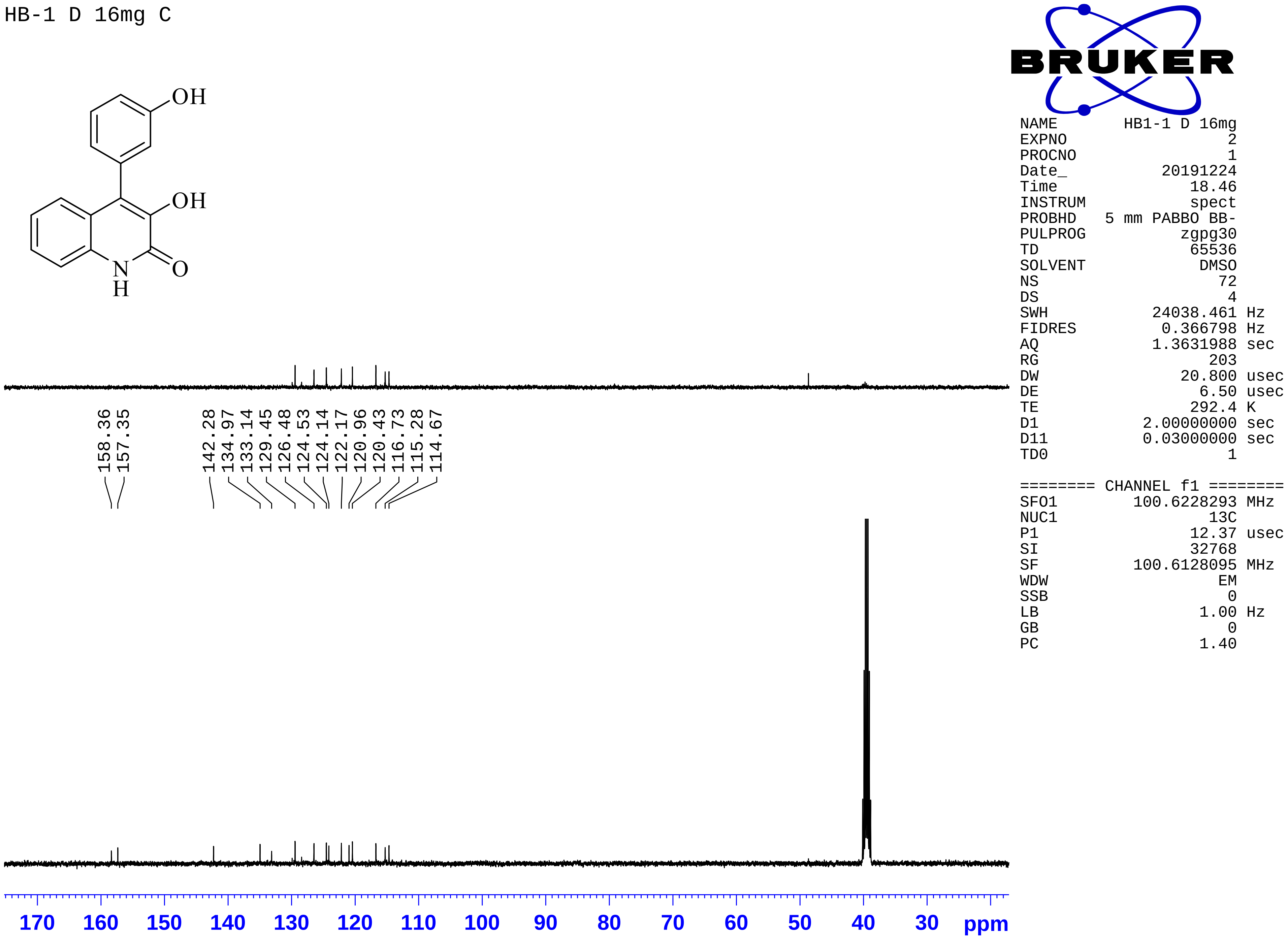

Figure S41. ^{13}C NMR spectrum of **13**.

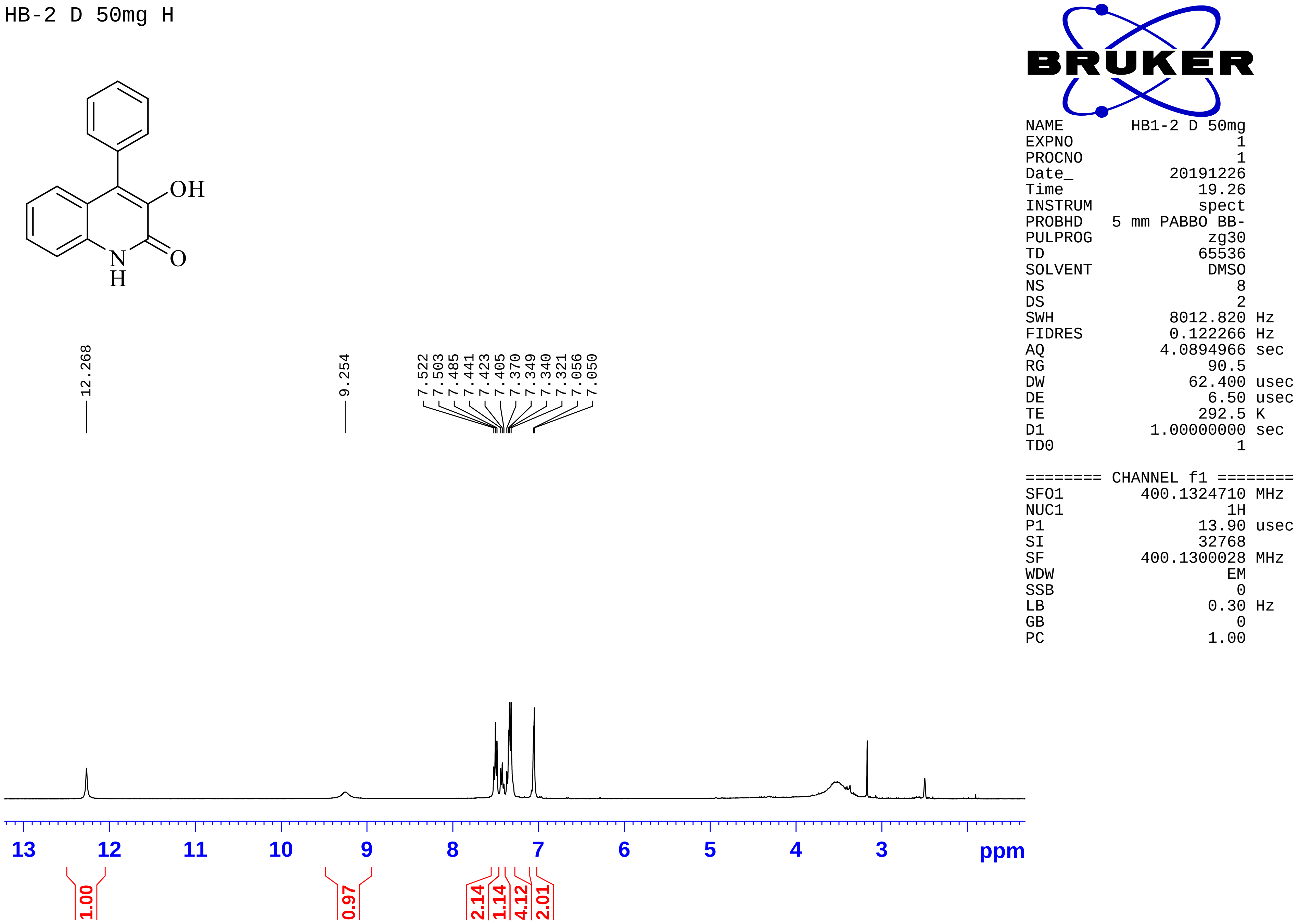

Figure S42. ^{1}H NMR spectrum of **14**.

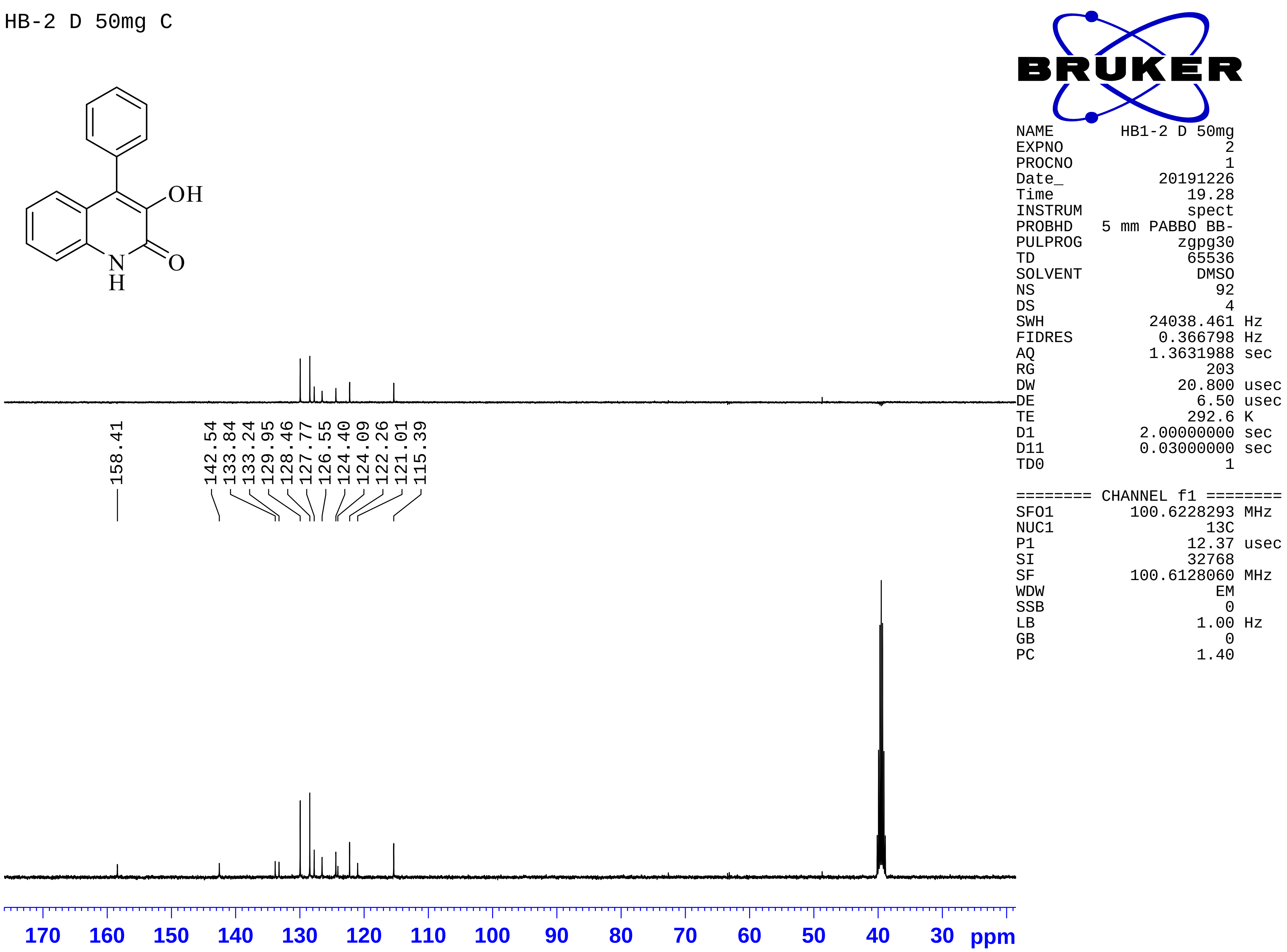

Figure S43. ^{13}C NMR spectrum of **14**.

HB2-2 M 3mg H

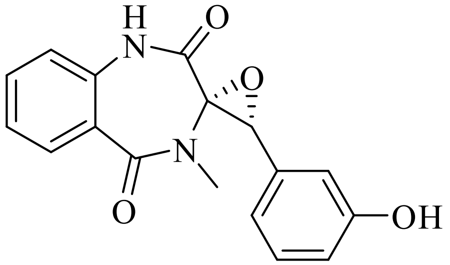

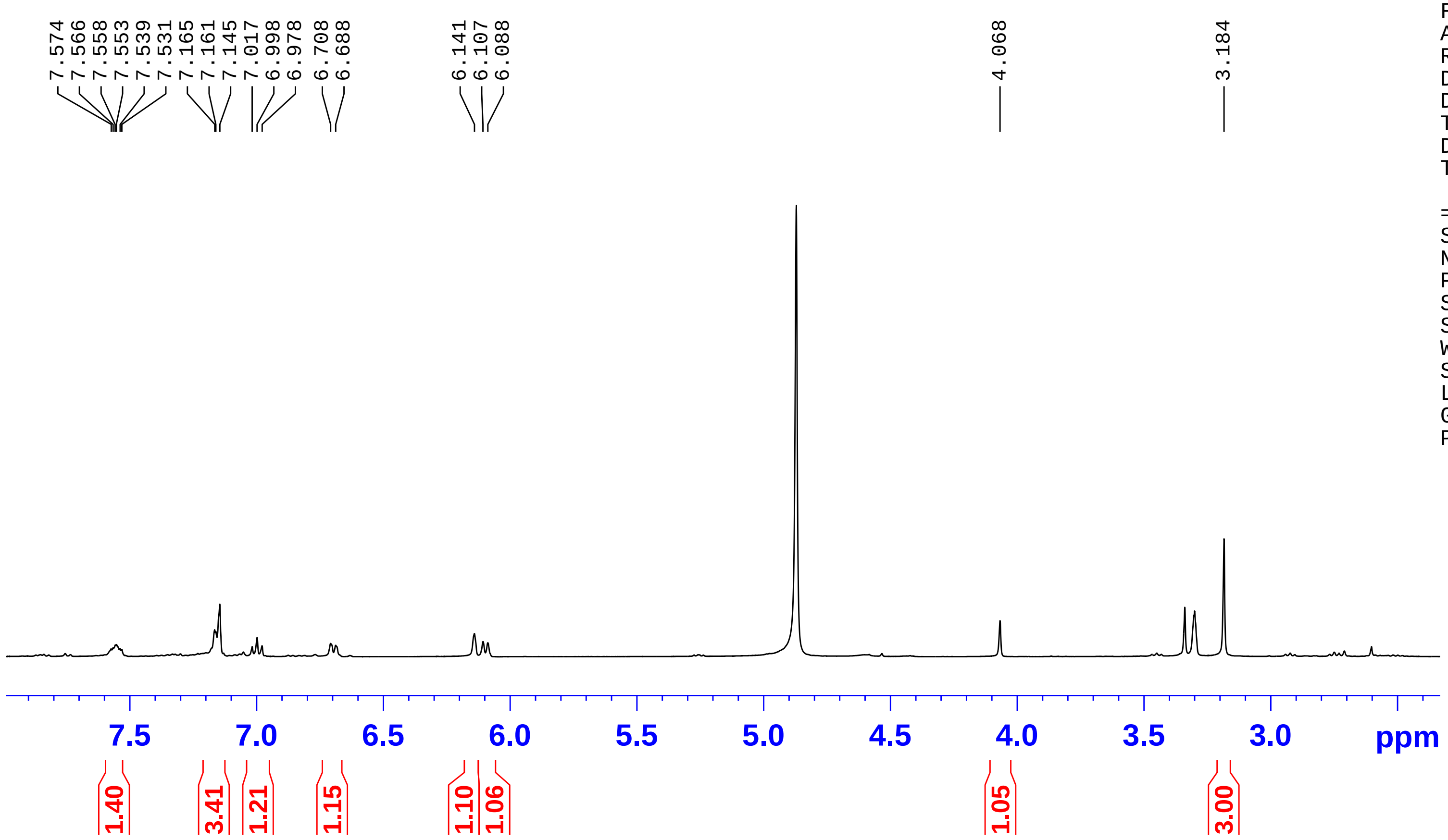

NAME	HB2-2 M 3mg	
EXPNO	1	
PROCNO	1	
Date_	20200726	
Time	13.45	
INSTRUM	spect	
PROBHD	5 mm PABBO BB-	
PULPROG	zg30	
TD	65536	
SOLVENT	MeOD	
NS	17	
DS	2	
SWH	8012.820	Hz
FIDRES	0.122266	Hz
AQ	4.0894966	sec
RG	203	
DW	62.400	usec
DE	6.50	usec
TE	296.3	K
D1	1.00000000	sec
TD0	1	

======== CHANNEL f1 ========

SFO1	400.1324710	MHz
NUC1	1H	
P1	13.90	usec
SI	32768	
SF	400.1300113	MHz
WDW	EM	
SSB	0	
LB	0.30	Hz
GB	0	
PC	1.00	

Figure S44. ^{1}H NMR spectrum of **15**.

HB2-2 M 3mg C

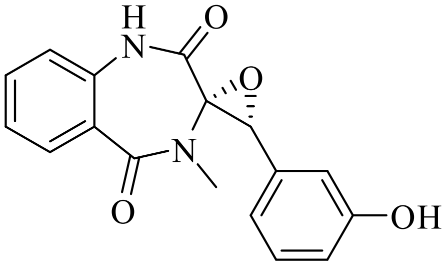

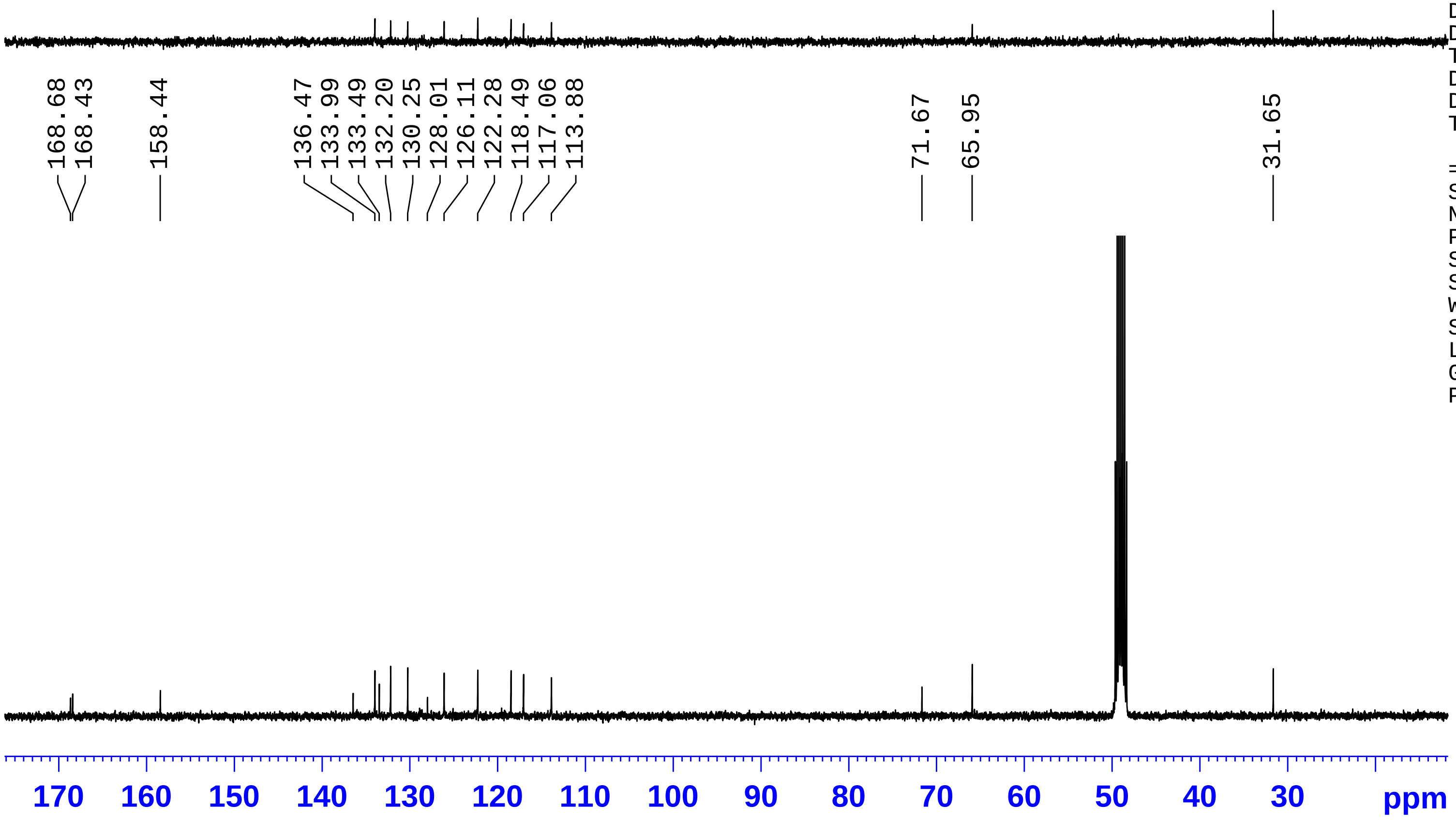

NAME	HB2-2 M 3mg	
EXPNO	2	
PROCNO	1	
Date_	20200726	
Time	13.48	
INSTRUM	spect	
PROBHD	5 mm PABBO BB-	
PULPROG	zgpg30	
TD	65536	
SOLVENT	MeOD	
NS	649	
DS	4	
SWH	24038.461	Hz
FIDRES	0.366798	Hz
AQ	1.3631988	sec
RG	203	
DW	20.800	usec
DE	6.50	usec
TE	296.4	K
D1	2.00000000	sec
D11	0.03000000	sec
TD0	1	
======== CHANNEL f1 ========		
SFO1	100.6228293	MHz
NUC1	13C	
P1	12.37	usec
SI	32768	
SF	100.6126288	MHz
WDW	EM	
SSB	0	
LB	1.00	Hz
GB	0	
PC	1.40	

Figure S45. ^{13}C NMR spectrum of **15**.

HB-C16 M 60mg H

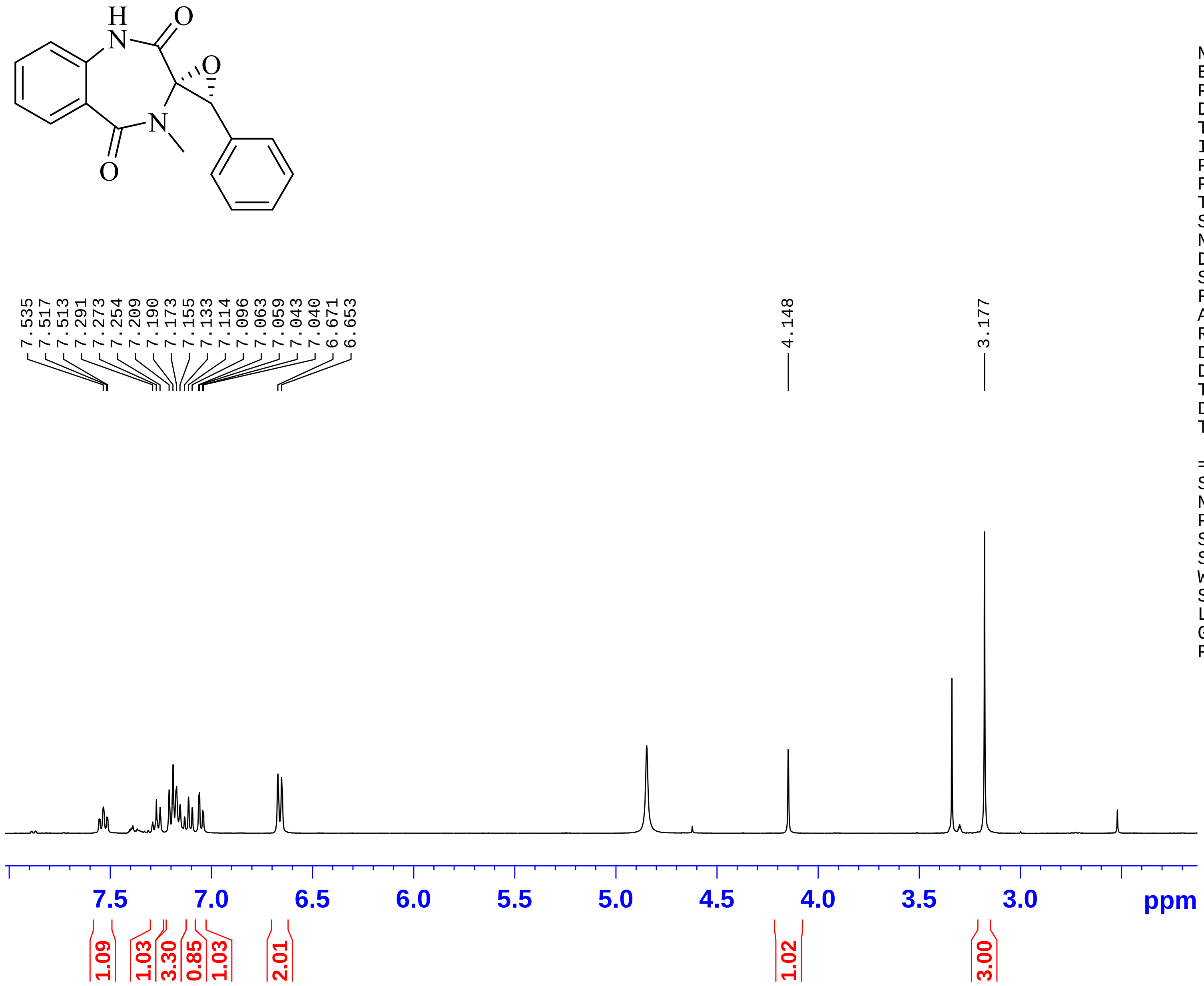


```
NAME          HB-C16 M 60mg
EXPNO                     1
PROCNO                    1
Date_              20200910
Time                  20.35
INSTRUM               spect
PROBHD     5 mm PABBO BB-
PULPROG                zg30
TD                    65536
SOLVENT                MeOD
NS                        9
DS                        2
SWH             8012.820 Hz
FIDRES          0.122266 Hz
AQ             4.0894966 sec
RG                       64
DW               62.400 usec
DE                 6.50 usec
TE                  296.4 K
D1           1.00000000 sec
TD0                       1

======== CHANNEL f1 ========
SFO1        400.1324710 MHz
NUC1                     1H
P1                13.90 usec
SI                    32768
SF          400.1300116 MHz
WDW                      EM
SSB                       0
LB                  0.30 Hz
GB                        0
PC                     1.00
```

Figure S46. ^{1}H NMR spectrum of **16**.

HB-C16 M 60mg C

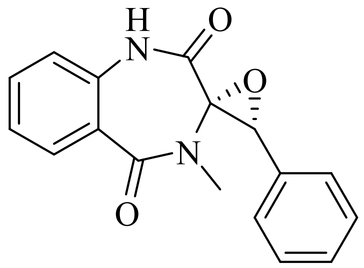

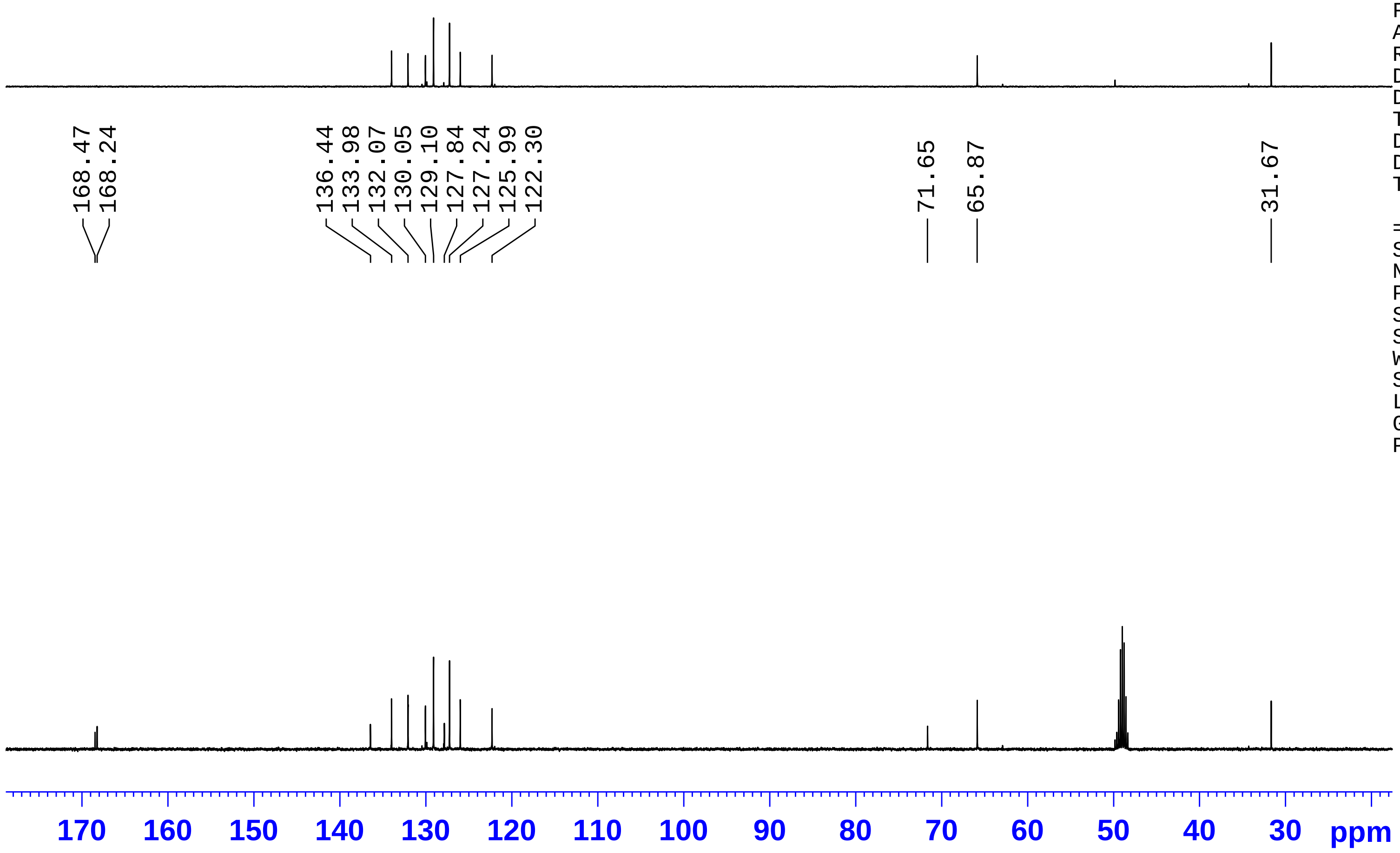

NAME	HB-C16 M 60mg
EXPNO	2
PROCNO	1
Date_	20200910
Time	20.38
INSTRUM	spect
PROBHD	5 mm PABBO BB-
PULPROG	zgpg30
TD	65536
SOLVENT	MeOD
NS	36
DS	4
SWH	24038.461 Hz
FIDRES	0.366798 Hz
AQ	1.3631988 sec
RG	203
DW	20.800 usec
DE	6.50 usec
TE	296.8 K
D1	2.00000000 sec
D11	0.03000000 sec
TD0	1

======== CHANNEL f1 ========

SFO1	100.6228293 MHz
NUC1	13C
P1	12.37 usec
SI	32768
SF	100.6126394 MHz
WDW	EM
SSB	0
LB	1.00 Hz
GB	0
PC	1.40

Figure S47. ^{13}C NMR spectrum of **16**.

HB-C11 M 8mg H

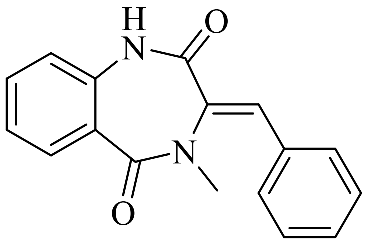

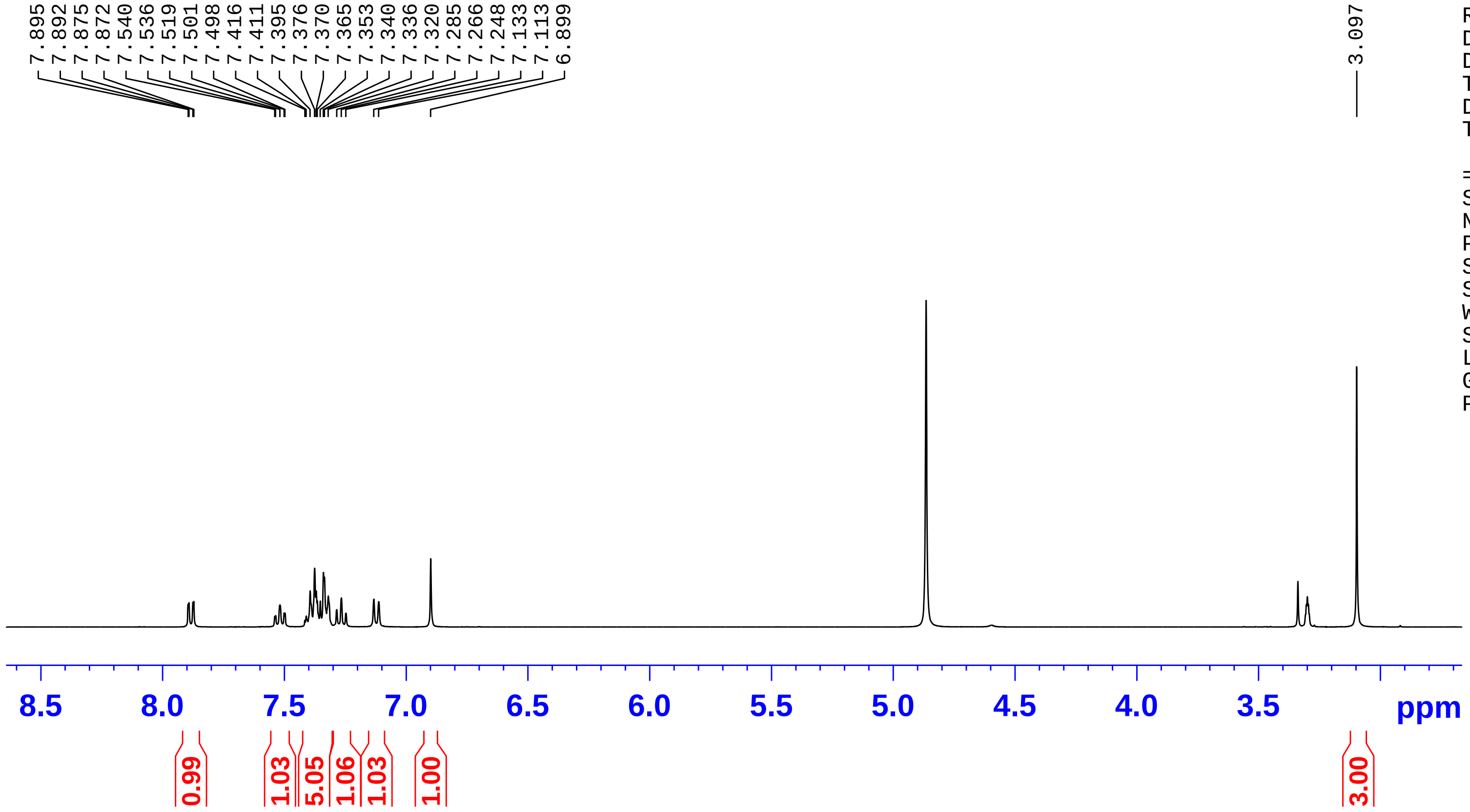

NAME	HB-C11 M 8mg
EXPNO	1
PROCNO	1
Date_	20200903
Time	19.01
INSTRUM	spect
PROBHD	5 mm PABBO BB-
PULPROG	zg30
TD	65536
SOLVENT	MeOD
NS	18
DS	2
SWH	8012.820 Hz
FIDRES	0.122266 Hz
AQ	4.0894966 sec
RG	181
DW	62.400 usec
DE	6.50 usec
TE	296.6 K
D1	1.00000000 sec
TD0	1
======== CHANNEL f1 ========	
SFO1	400.1324710 MHz
NUC1	1H
P1	13.90 usec
SI	32768
SF	400.1300116 MHz
WDW	EM
SSB	0
LB	0.30 Hz
GB	0
PC	1.00

Figure S48. ^{1}H NMR spectrum of **17**.

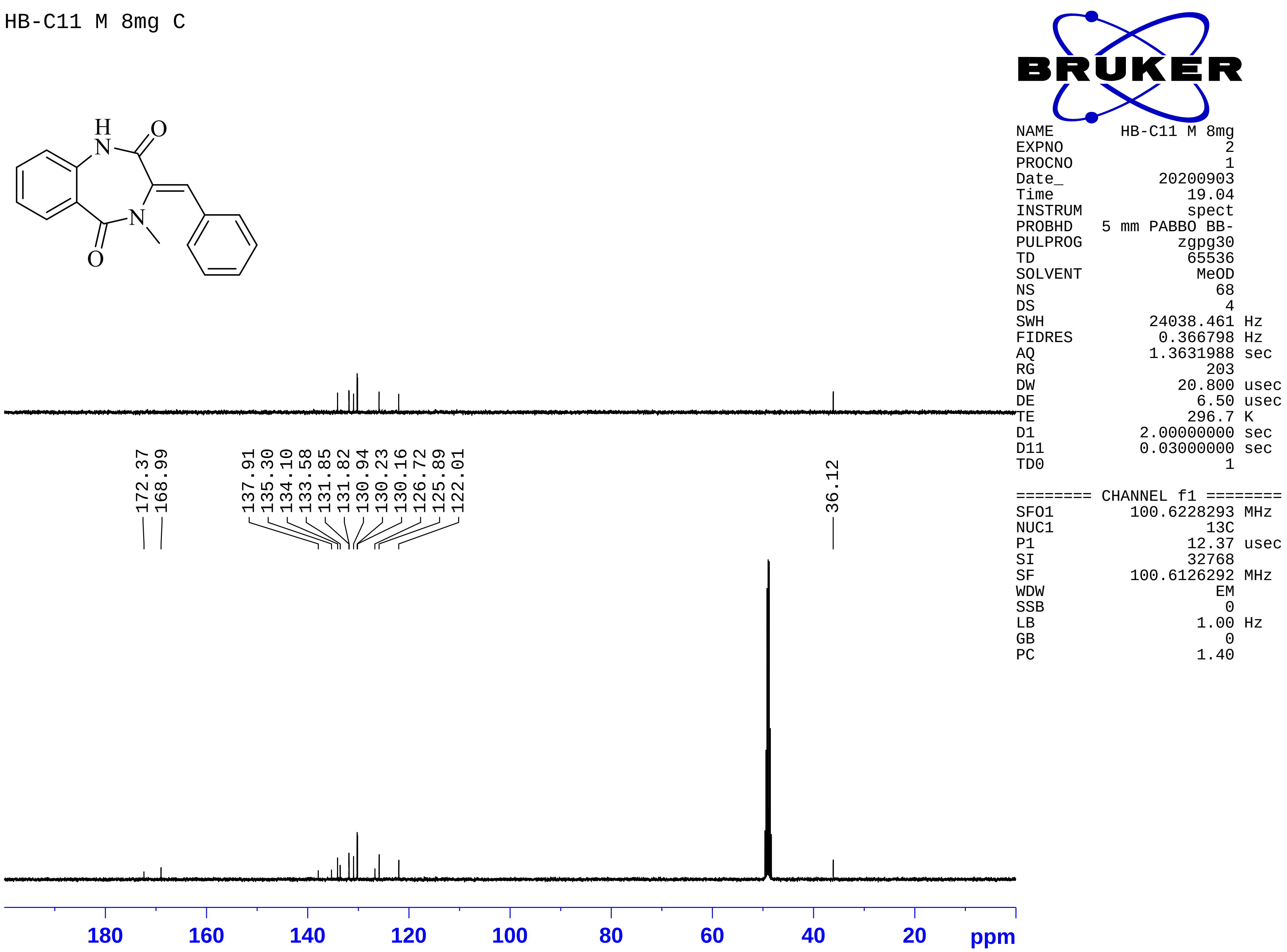

Figure S49. ^{13}C NMR spectrum of **17**.

HB2-11 D 3mg H

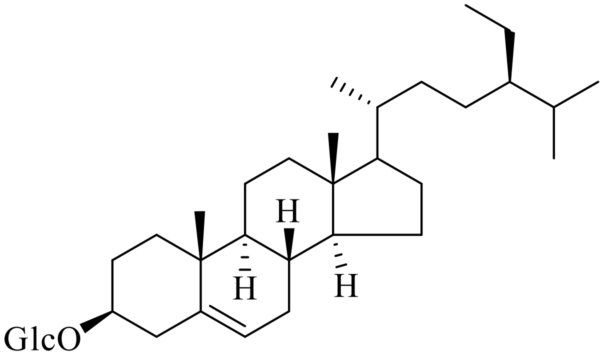

NAME	HB2-11 D 3mg	
EXPNO	1	
PROCNO	1	
Date_	20200805	
Time	10.32	
INSTRUM	spect	
PROBHD	5 mm PABBO BB-	
PULPROG	zg30	
TD	65536	
SOLVENT	DMSO	
NS	18	
DS	2	
SWH	8012.820	Hz
FIDRES	0.122266	Hz
AQ	4.0894966	sec
RG	181	
DW	62.400	usec
DE	6.50	usec
TE	295.9	K
D1	1.00000000	sec
TD0	1	

======== CHANNEL f1 ========		
SFO1	400.1324710	MHz
NUC1	1H	
P1	13.90	usec
SI	32768	
SF	400.1300032	MHz
WDW	EM	
SSB	0	
LB	0.30	Hz
GB	0	
PC	1.00	

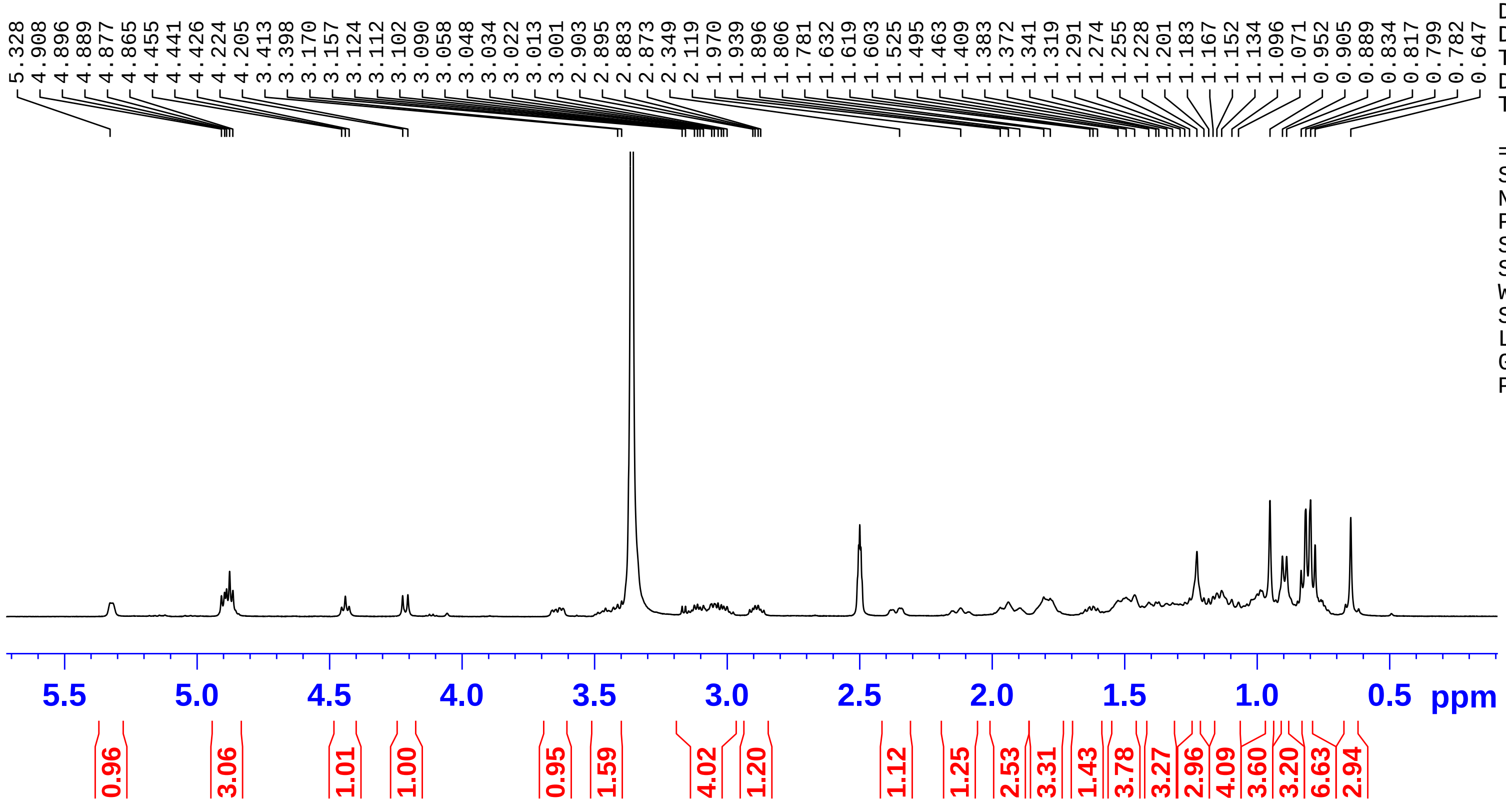

Figure S50. ^{1}H NMR spectrum of **18**.

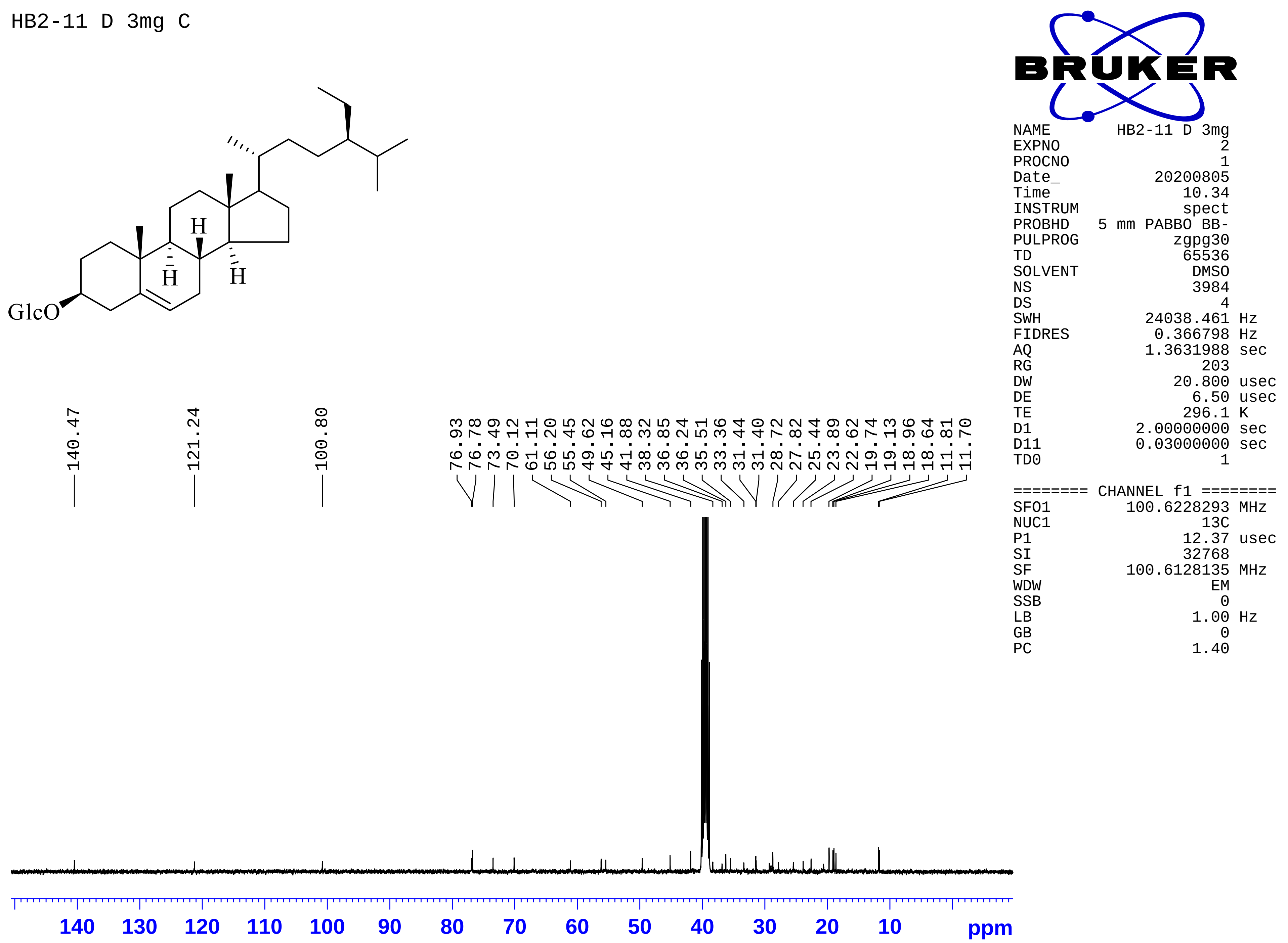

Figure S51. ^{13}C NMR spectrum of **18**.

HB2-1 M 7mg H

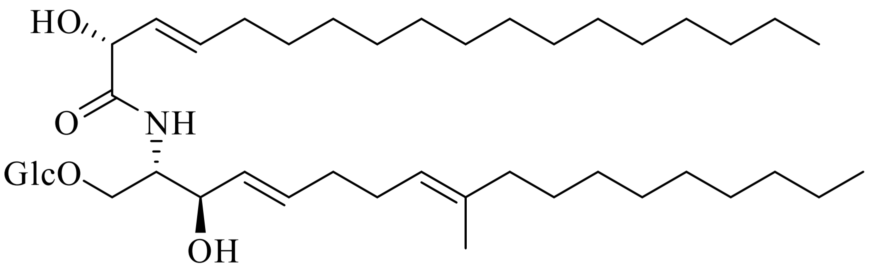

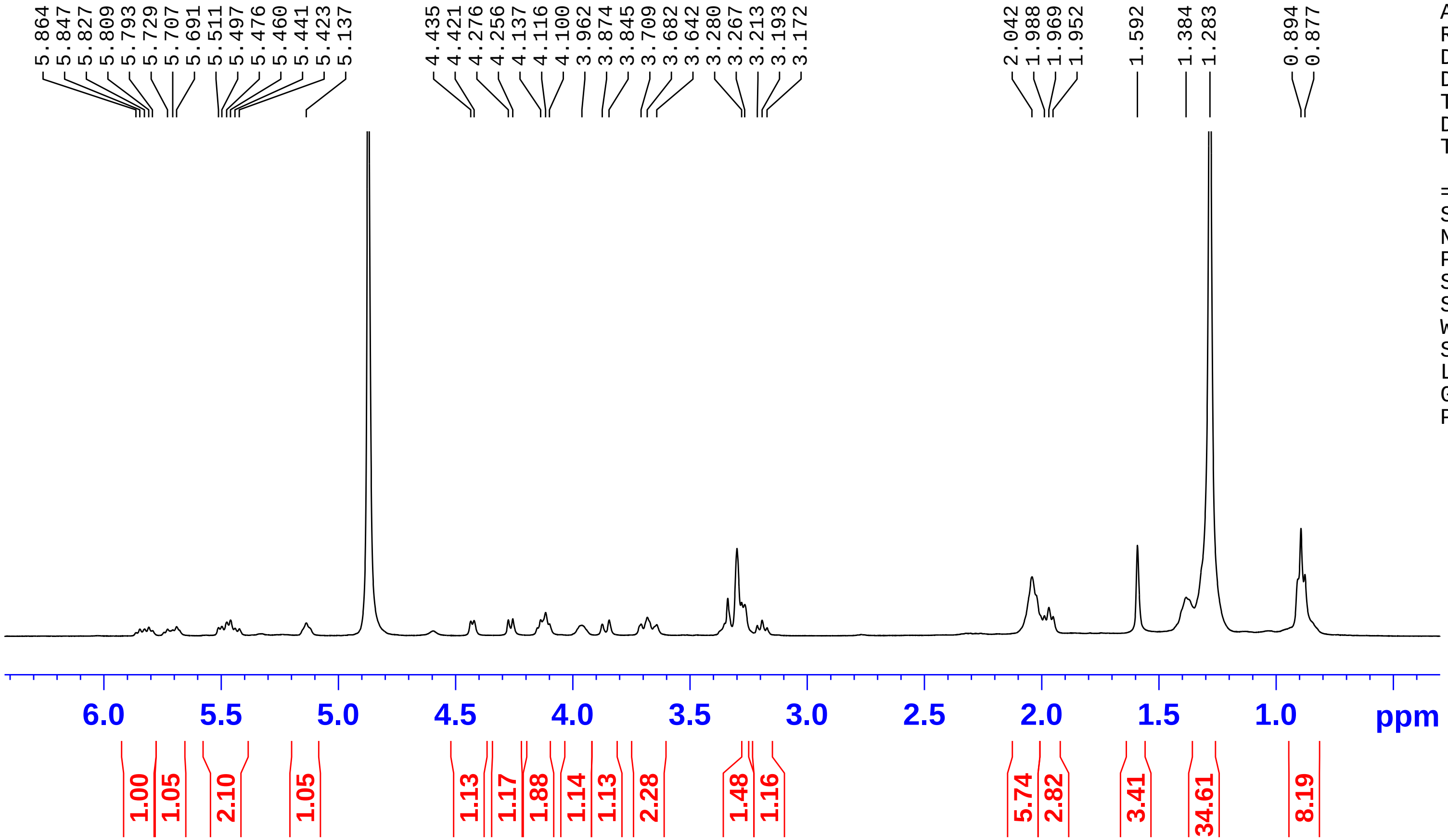

NAME	HB2-1 M 7mg
EXPNO	1
PROCNO	1
Date_	20200723
Time	19.44
INSTRUM	spect
PROBHD	5 mm PABBO BB-
PULPROG	zg30
TD	65536
SOLVENT	MeOD
NS	19
DS	2
SWH	8012.820 Hz
FIDRES	0.122266 Hz
AQ	4.0894966 sec
RG	144
DW	62.400 usec
DE	6.50 usec
TE	295.9 K
D1	1.00000000 sec
TD0	1

======== CHANNEL f1 ========

SFO1	400.1324710 MHz
NUC1	1H
P1	13.90 usec
SI	32768
SF	400.1300115 MHz
WDW	EM
SSB	0
LB	0.30 Hz
GB	0
PC	1.00

Figure S52. ^{1}H NMR spectrum of **19**.

HB2-1 M 7mg C

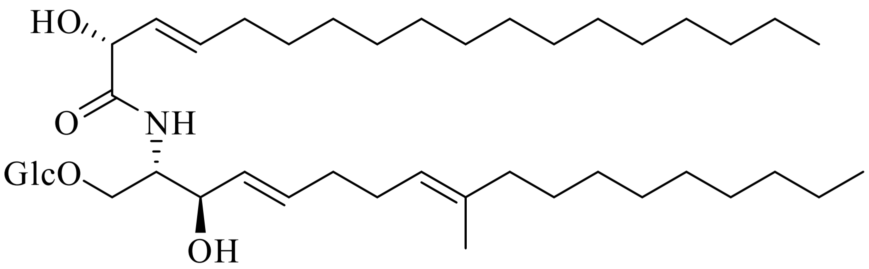

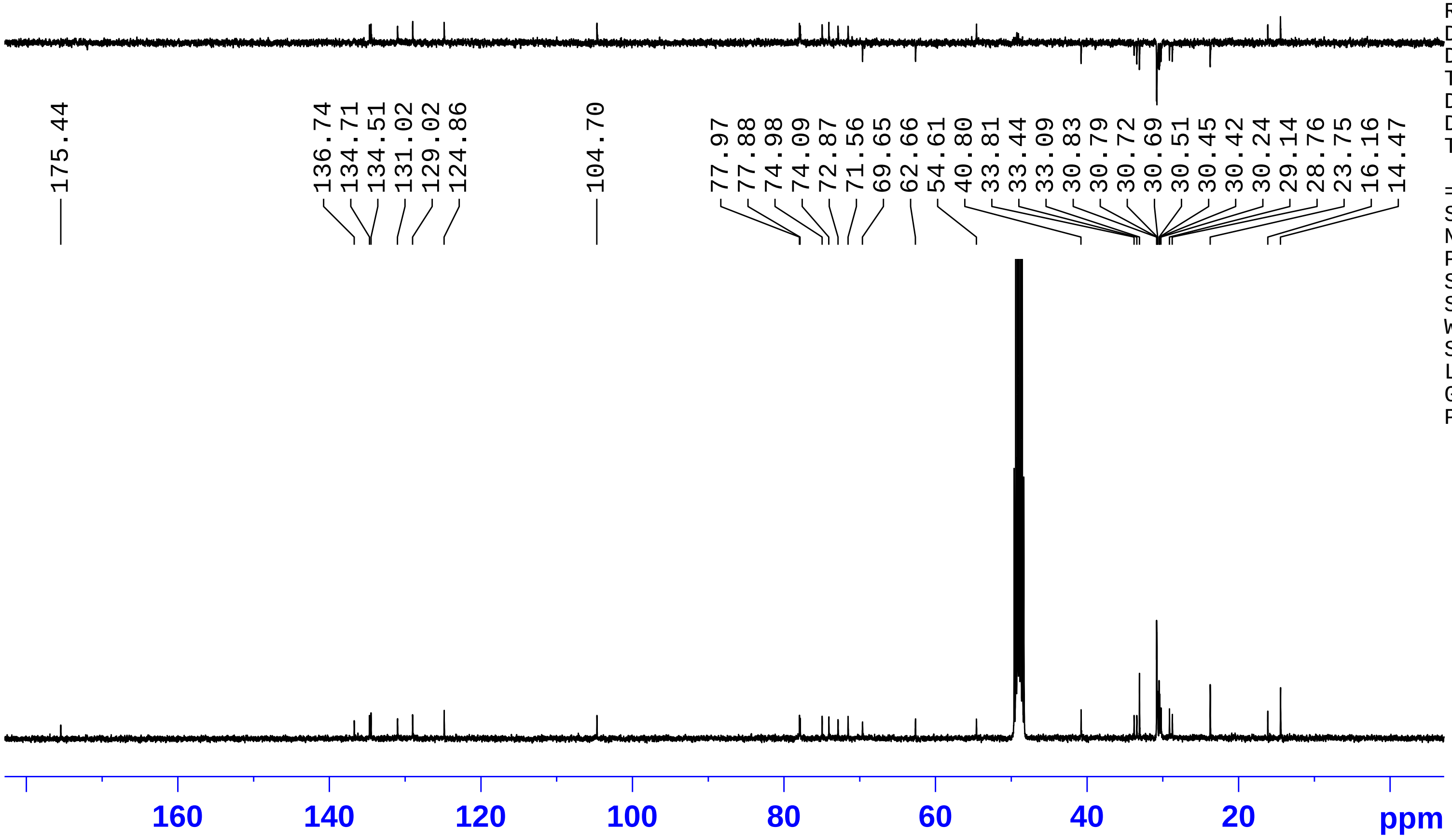

BRUKER

NAME	HB2-1 M 7mg
EXPNO	2
PROCNO	1
Date_	20200723
Time	19.47
INSTRUM	spect
PROBHD	5 mm PABBO BB-
PULPROG	zgpg30
TD	65536
SOLVENT	MeOD
NS	1494
DS	4
SWH	24038.461 Hz
FIDRES	0.366798 Hz
AQ	1.3631988 sec
RG	203
DW	20.800 usec
DE	6.50 usec
TE	296.2 K
D1	2.00000000 sec
D11	0.03000000 sec
TD0	1

======== CHANNEL f1 ========

SFO1	100.6228293 MHz
NUC1	13C
P1	12.37 usec
SI	32768
SF	100.6126284 MHz
WDW	EM
SSB	0
LB	1.00 Hz
GB	0
PC	1.40

Figure S53. ^{13}C NMR spectrum of **19**.

HB-C17 M 8mg H

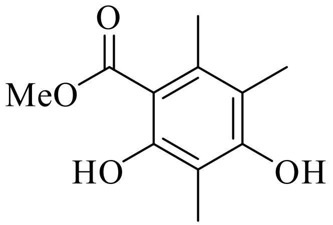

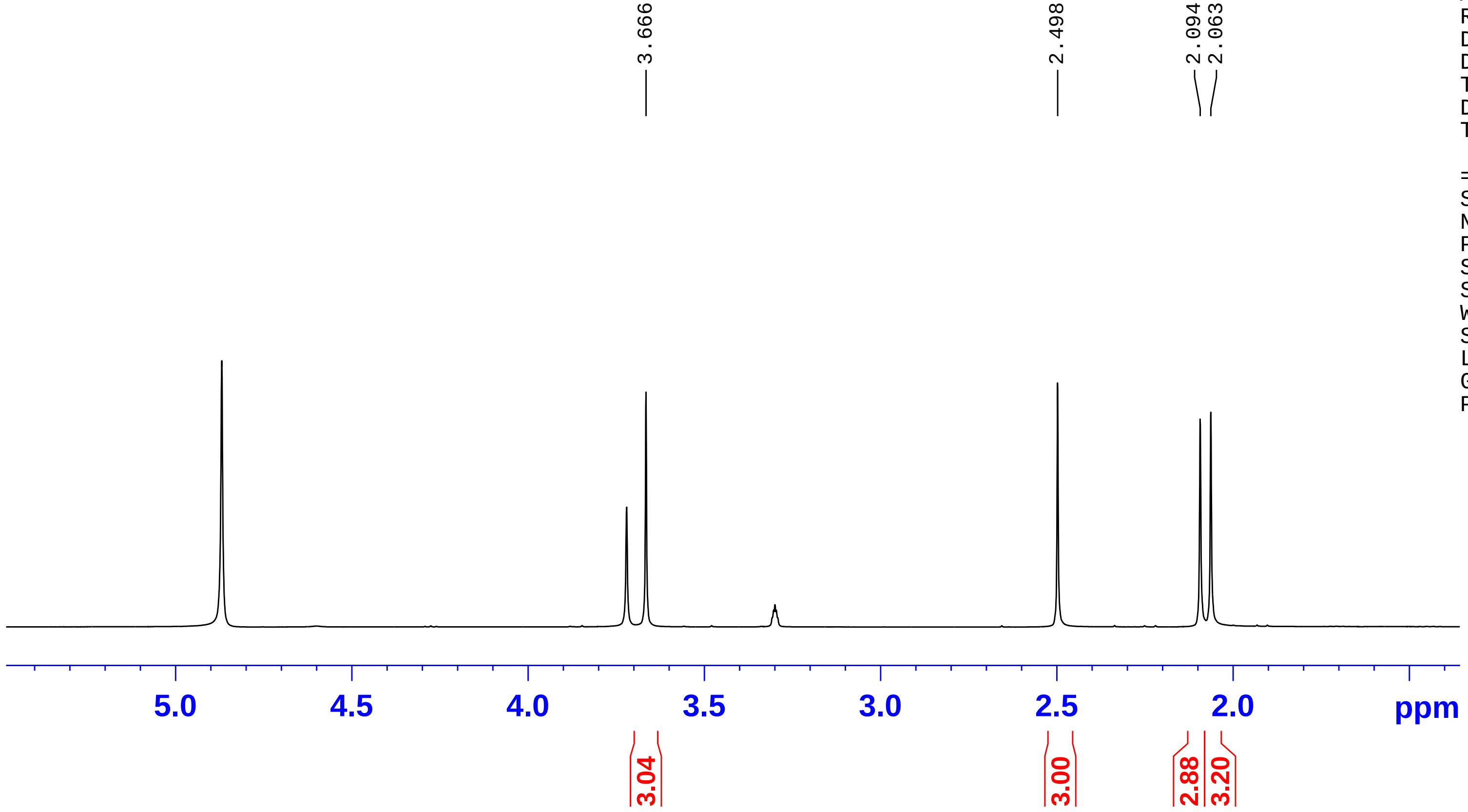


```
NAME          HB-C17 M 8mg
EXPNO                    1
PROCNO                   1
Date_             20201203
Time                 20.33
INSTRUM              spect
PROBHD    5 mm PABBO BB-
PULPROG               zg30
TD                   65536
SOLVENT               MeOD
NS                      15
DS                       2
SWH            8012.820 Hz
FIDRES         0.122266 Hz
AQ            4.0894966 sec
RG                     161
DW              62.400 usec
DE                6.50 usec
TE                 295.8 K
D1          1.00000000 sec
TD0                      1

======== CHANNEL f1 ========
SFO1        400.1324710 MHz
NUC1                    1H
P1                13.90 usec
SI                   32768
SF          400.1300116 MHz
WDW                     EM
SSB                      0
LB                 0.30 Hz
GB                       0
PC                    1.00
```

Figure S54. ^{1}H NMR spectrum of **20**.

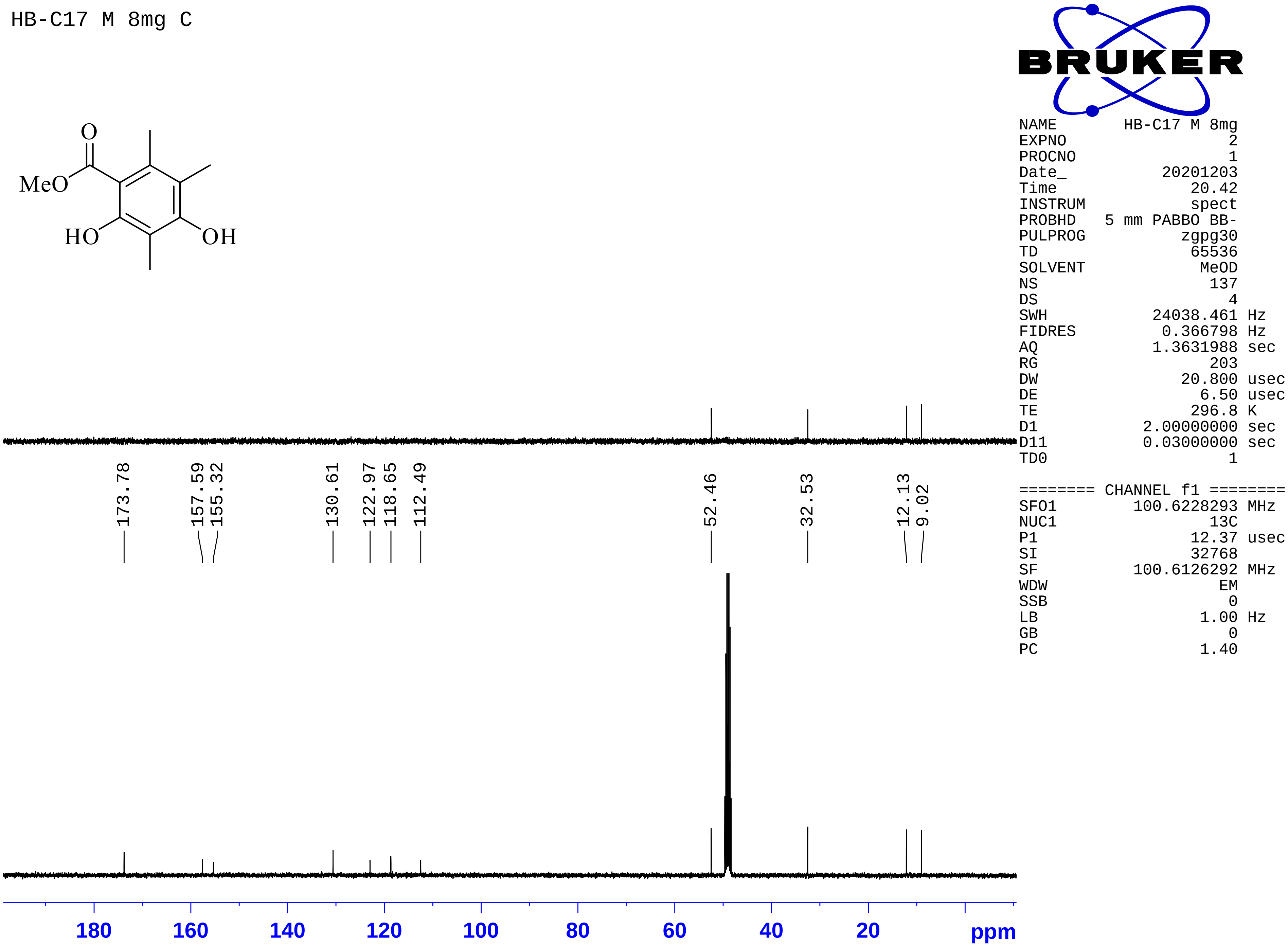

Figure S55. ^{13}C NMR spectrum of **20**.

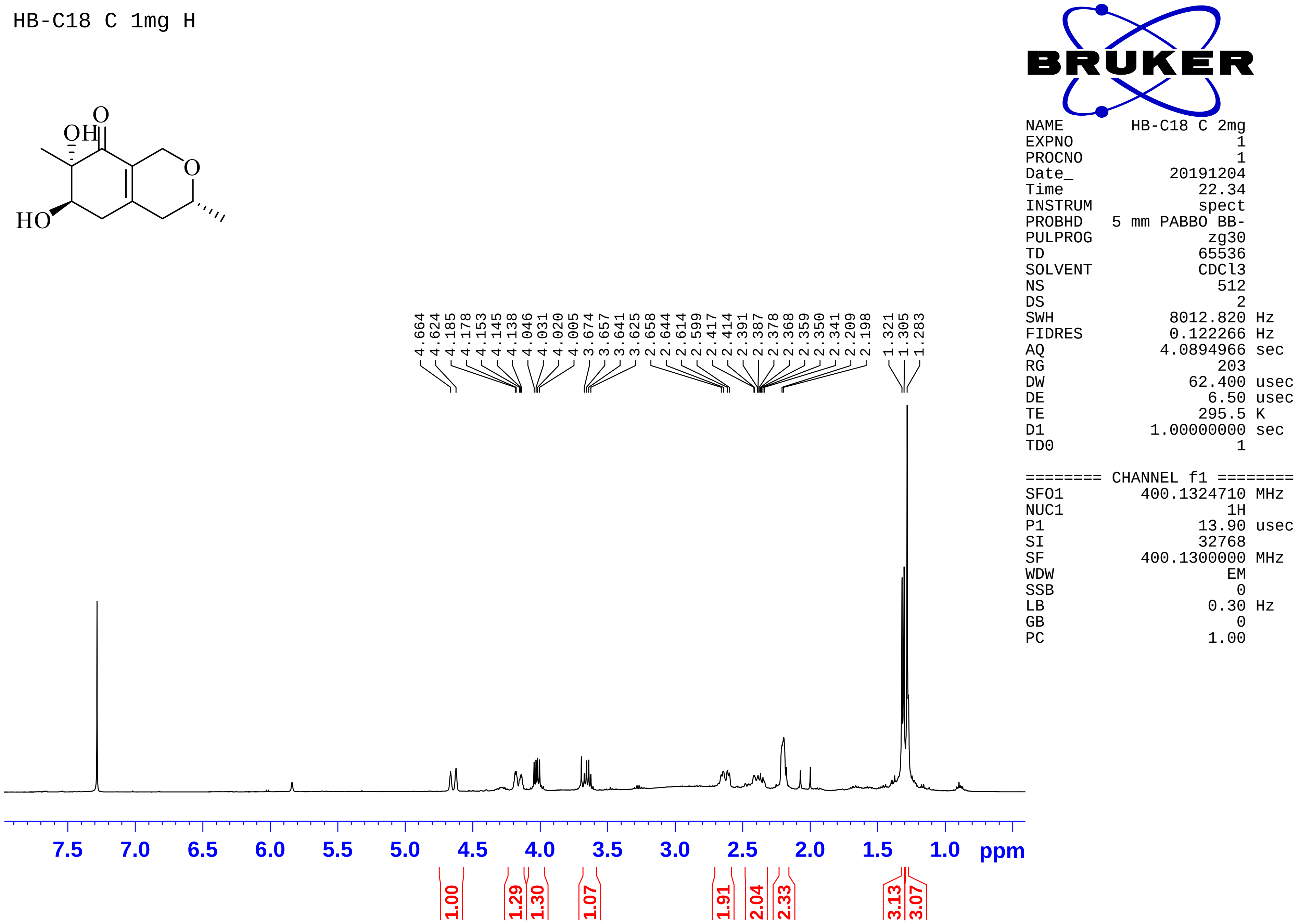

Figure S56. ^{1}H NMR spectrum of **21**.

HB-C18 C 1mg C

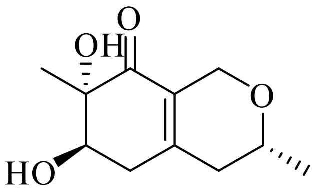

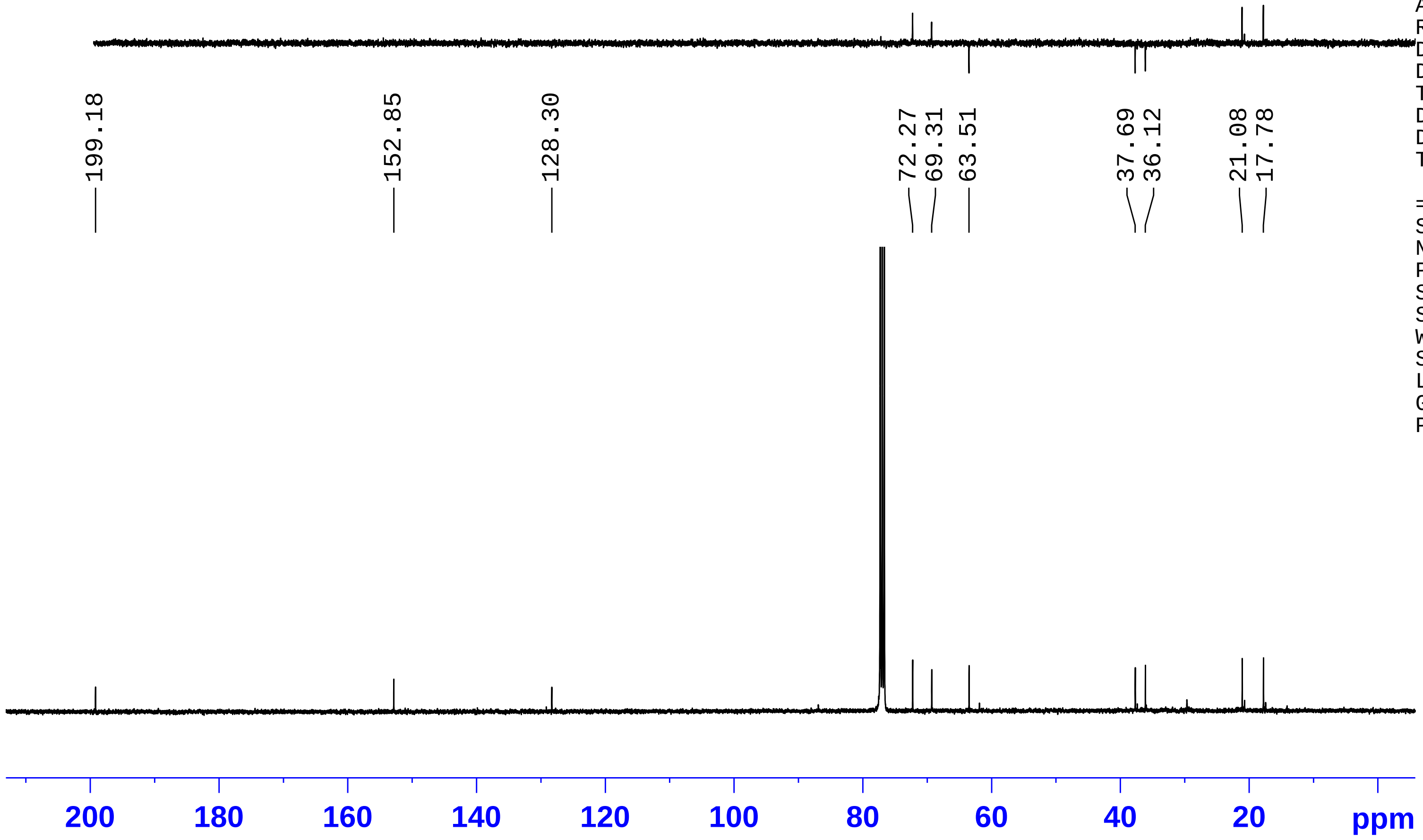

Figure S57. ^{13}C NMR spectrum of **21**.

HB-C19 C 1mg H

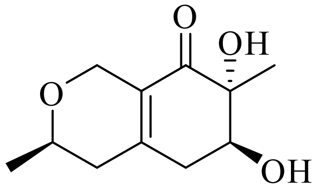

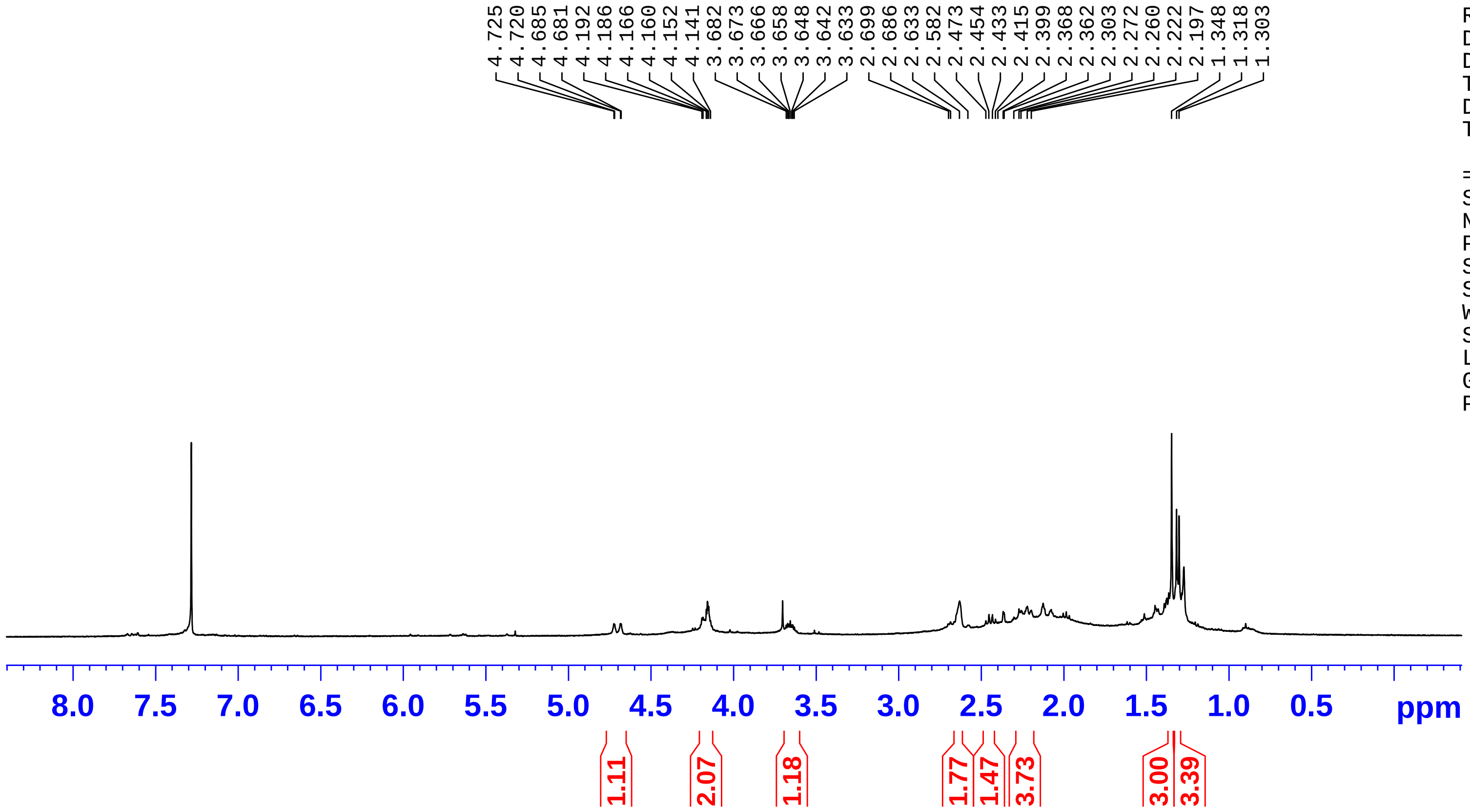

NAME	HB-C19 C 1.5mg
EXPNO	1
PROCNO	1
Date_	20200920
Time	14.47
INSTRUM	spect
PROBHD	5 mm PABBO BB-
PULPROG	zg30
TD	65536
SOLVENT	CDCl3
NS	27
DS	2
SWH	8012.820 Hz
FIDRES	0.122266 Hz
AQ	4.0894966 sec
RG	203
DW	62.400 usec
DE	6.50 usec
TE	296.0 K
D1	1.00000000 sec
TD0	1

======== CHANNEL f1 ========

SFO1	400.1324710 MHz
NUC1	1H
P1	13.90 usec
SI	32768
SF	400.1300000 MHz
WDW	EM
SSB	0
LB	0.30 Hz
GB	0
PC	1.00

Figure S58. ^{1}H NMR spectrum of **22**.

HB-C19 C 1mg C

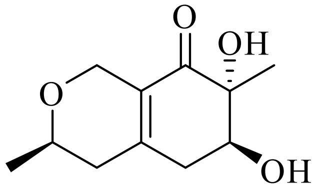

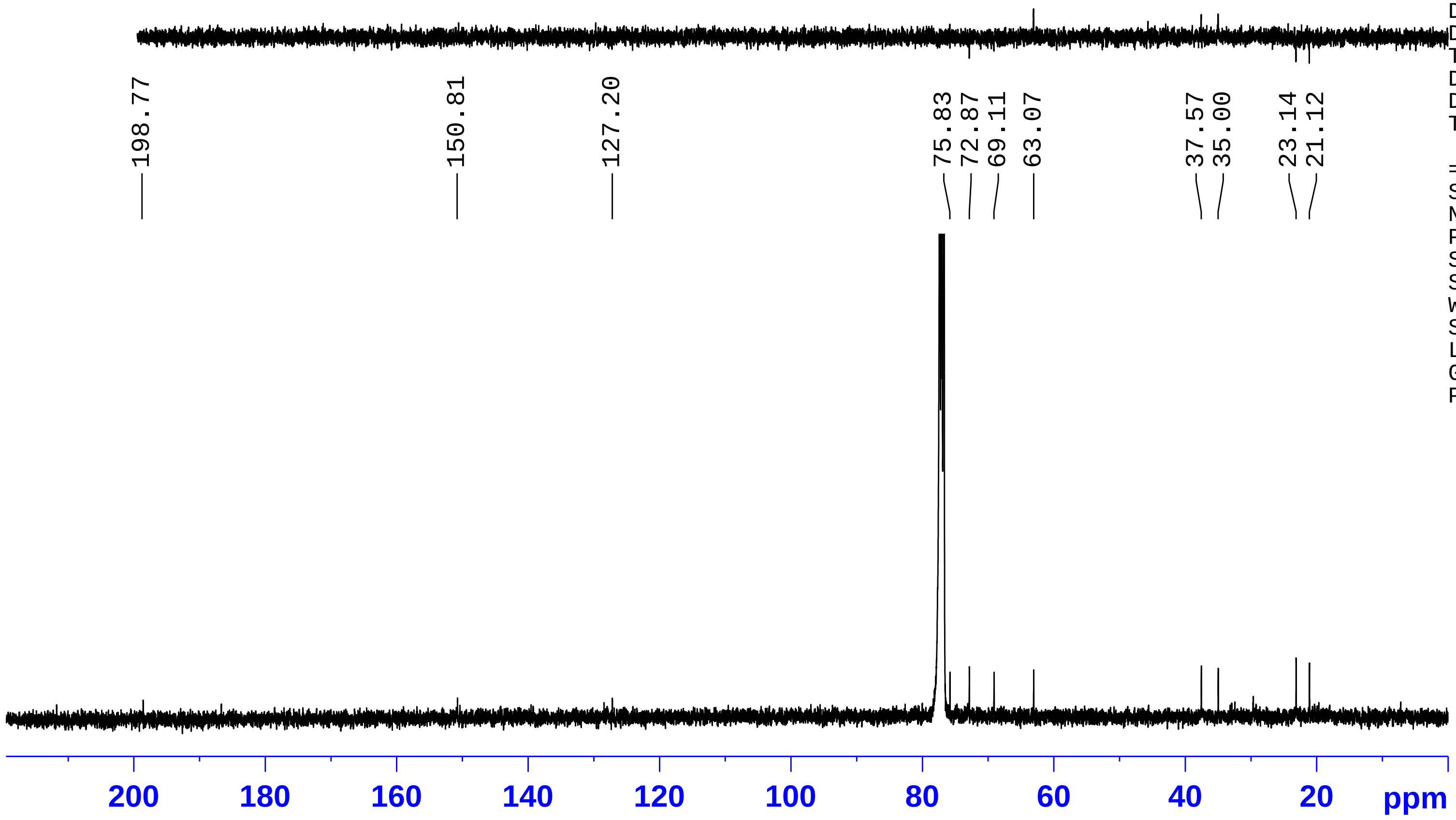

NAME	HB-C19 C 1.5mg
EXPNO	2
PROCNO	1
Date_	20200921
Time	6.33
INSTRUM	spect
PROBHD	5 mm PABBO BB-
PULPROG	zgpg30
TD	65536
SOLVENT	CDCl3
NS	15360
DS	4
SWH	24038.461 Hz
FIDRES	0.366798 Hz
AQ	1.3631988 sec
RG	203
DW	20.800 usec
DE	6.50 usec
TE	297.1 K
D1	2.00000000 sec
D11	0.03000000 sec
TD0	1

======== CHANNEL f1 ========	
SFO1	100.6228293 MHz
NUC1	13C
P1	12.37 usec
SI	32768
SF	100.6127685 MHz
WDW	EM
SSB	0
LB	1.00 Hz
GB	0
PC	1.40

Figure S59. ^{13}C NMR spectrum of **22**.

HB-C15 M 2mg H

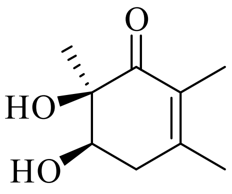

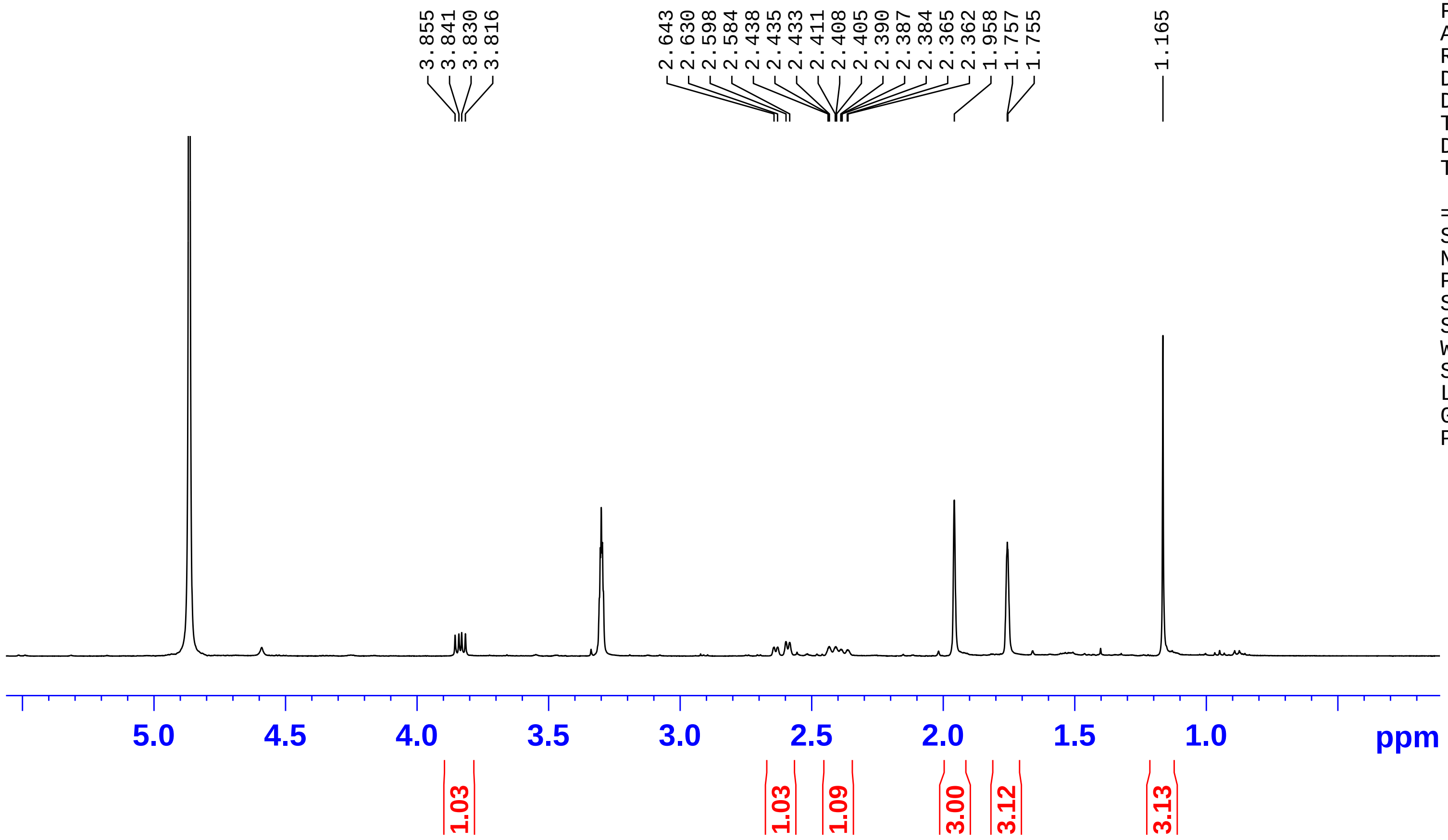

NAME	HB-C15 M 2mg
EXPNO	1
PROCNO	1
Date_	20200910
Time	21.08
INSTRUM	spect
PROBHD	5 mm PABBO BB-
PULPROG	zg30
TD	65536
SOLVENT	MeOD
NS	21
DS	2
SWH	8012.820 Hz
FIDRES	0.122266 Hz
AQ	4.0894966 sec
RG	203
DW	62.400 usec
DE	6.50 usec
TE	296.5 K
D1	1.00000000 sec
TD0	1

======== CHANNEL f1 ========

SFO1	400.1324710 MHz
NUC1	1H
P1	13.90 usec
SI	32768
SF	400.1300117 MHz
WDW	EM
SSB	0
LB	0.30 Hz
GB	0
PC	1.00

Figure S60. ^{1}H NMR spectrum of **23**.

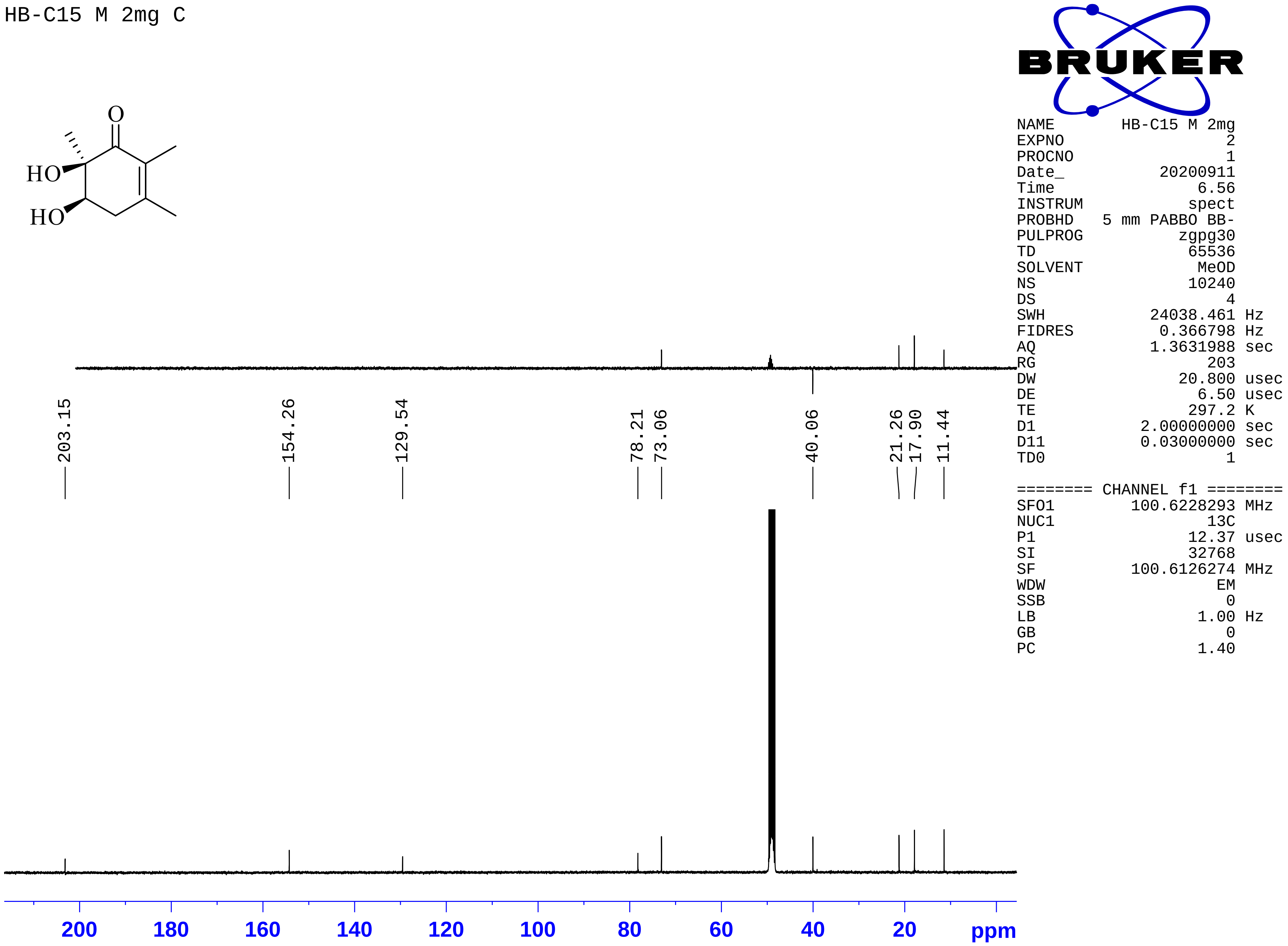

Figure S61. ^{13}C NMR spectrum of **23**.

HB4-9 C 5mg H

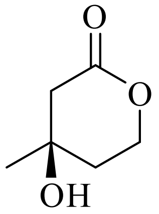

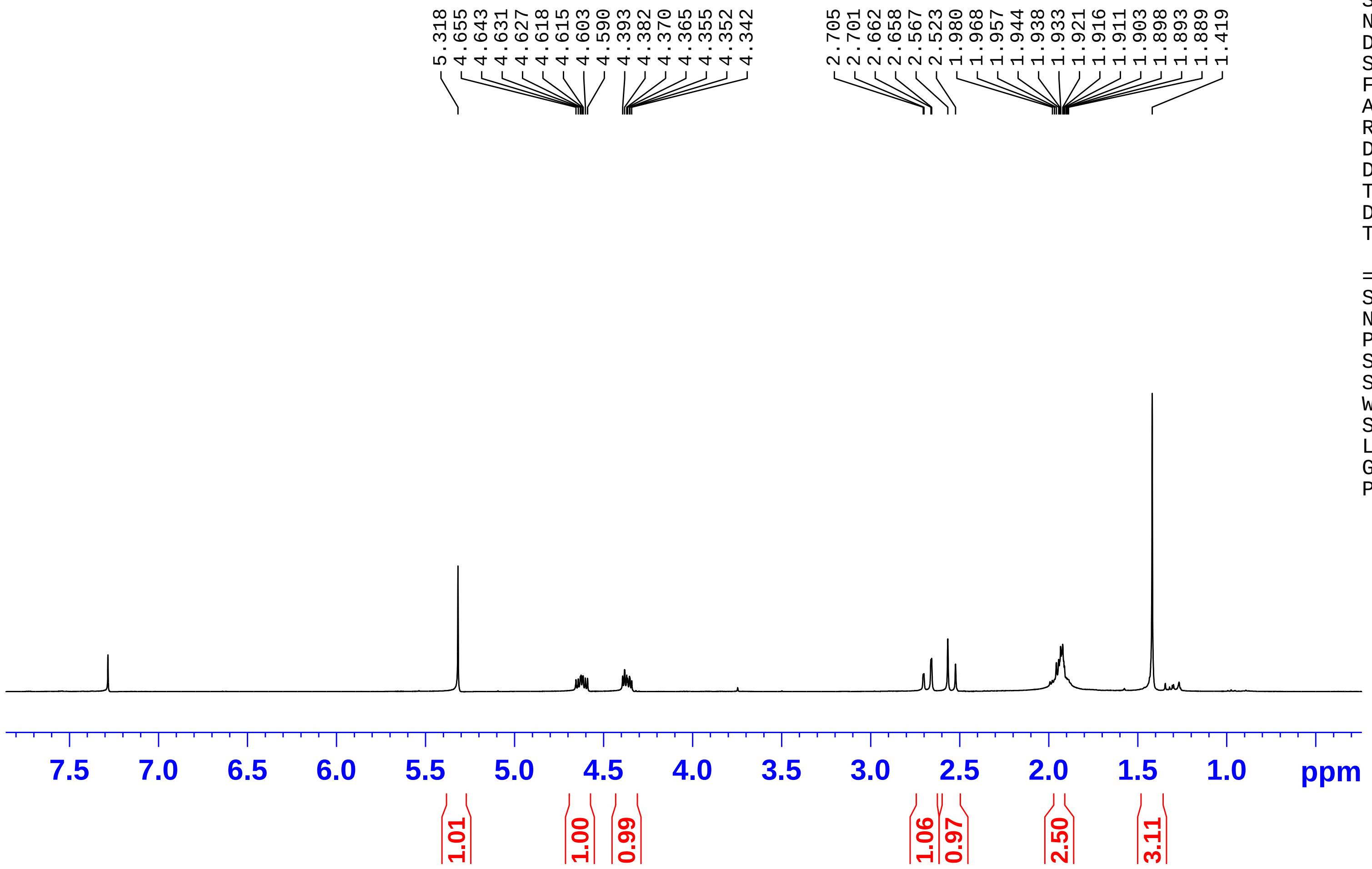

NAME	HB4-9 C 5mg	
EXPNO	1	
PROCNO	1	
Date_	20200611	
Time	15.03	
INSTRUM	spect	
PROBHD	5 mm PABBO BB-	
PULPROG	zg30	
TD	65536	
SOLVENT	CDCl3	
NS	27	
DS	2	
SWH	8012.820	Hz
FIDRES	0.122266	Hz
AQ	4.0894966	sec
RG	203	
DW	62.400	usec
DE	6.50	usec
TE	296.8	K
D1	1.00000000	sec
TD0	1	

======== CHANNEL f1 ========

SFO1	400.1324710	MHz
NUC1	1H	
P1	13.90	usec
SI	32768	
SF	400.1300000	MHz
WDW	EM	
SSB	0	
LB	0.30	Hz
GB	0	
PC	1.00	

Figure S62. ^{1}H NMR spectrum of **24**.

HB4-9 C 5mg C

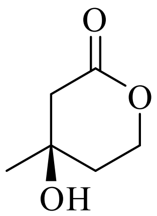

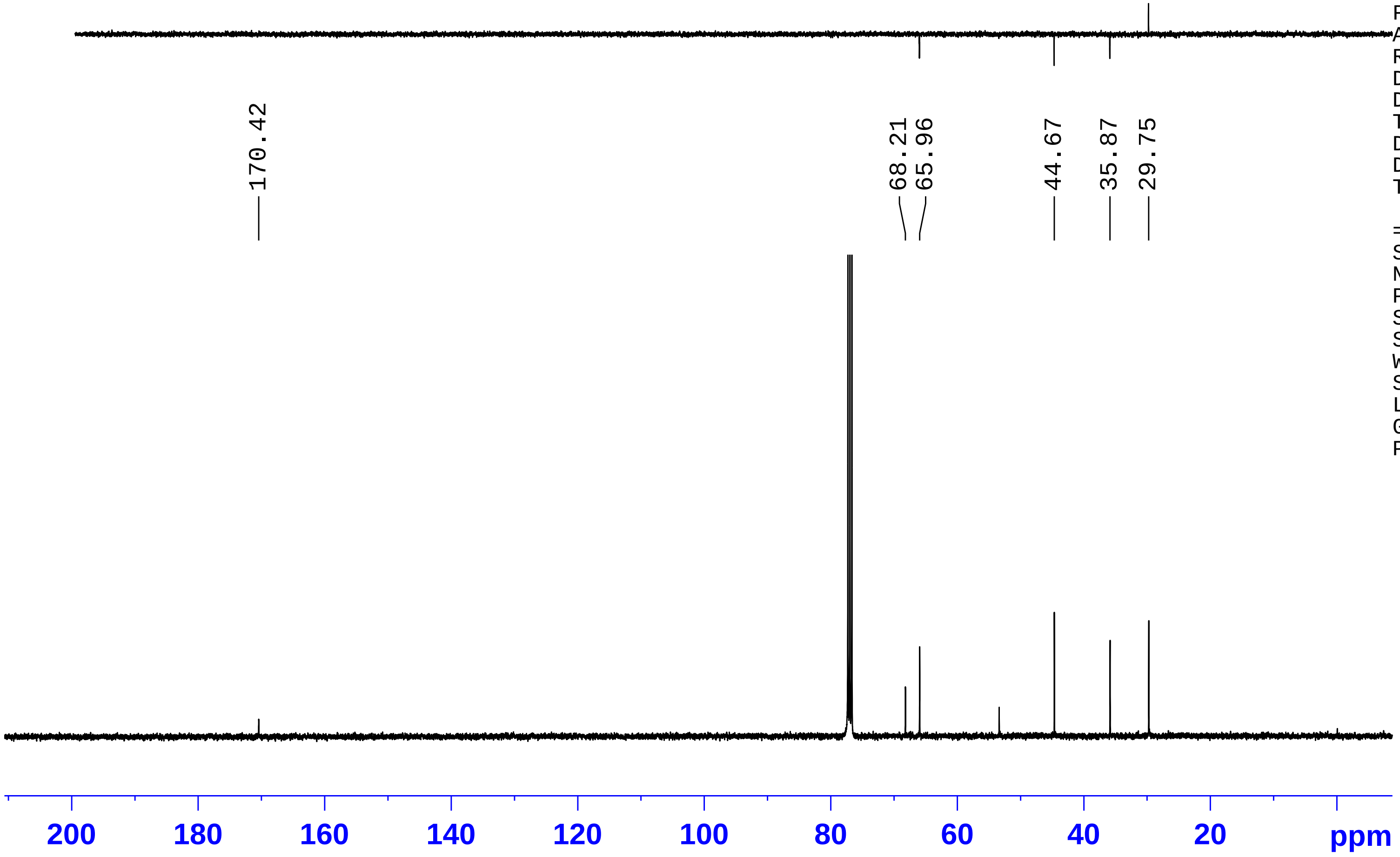

NAME HB4-9 C 5mg
EXPNO 2
PROCNO 1
Date_ 20200611
Time 15.07
INSTRUM spect
PROBHD 5 mm PABBO BB-
PULPROG zgpg30
TD 65536
SOLVENT CDCl3
NS 1020
DS 4
SWH 24038.461 Hz
FIDRES 0.366798 Hz
AQ 1.3631988 sec
RG 203
DW 20.800 usec
DE 6.50 usec
TE 297.0 K
D1 2.00000000 sec
D11 0.03000000 sec
TD0 1

======== CHANNEL f1 ========
SFO1 100.6228293 MHz
NUC1 13C
P1 12.37 usec
SI 32768
SF 100.6127729 MHz
WDW EM
SSB 0
LB 1.00 Hz
GB 0
PC 1.40

Figure S63. ^{13}C NMR spectrum of **24**.

HB-C12 M 26mg H

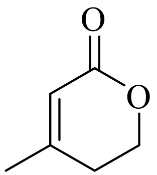

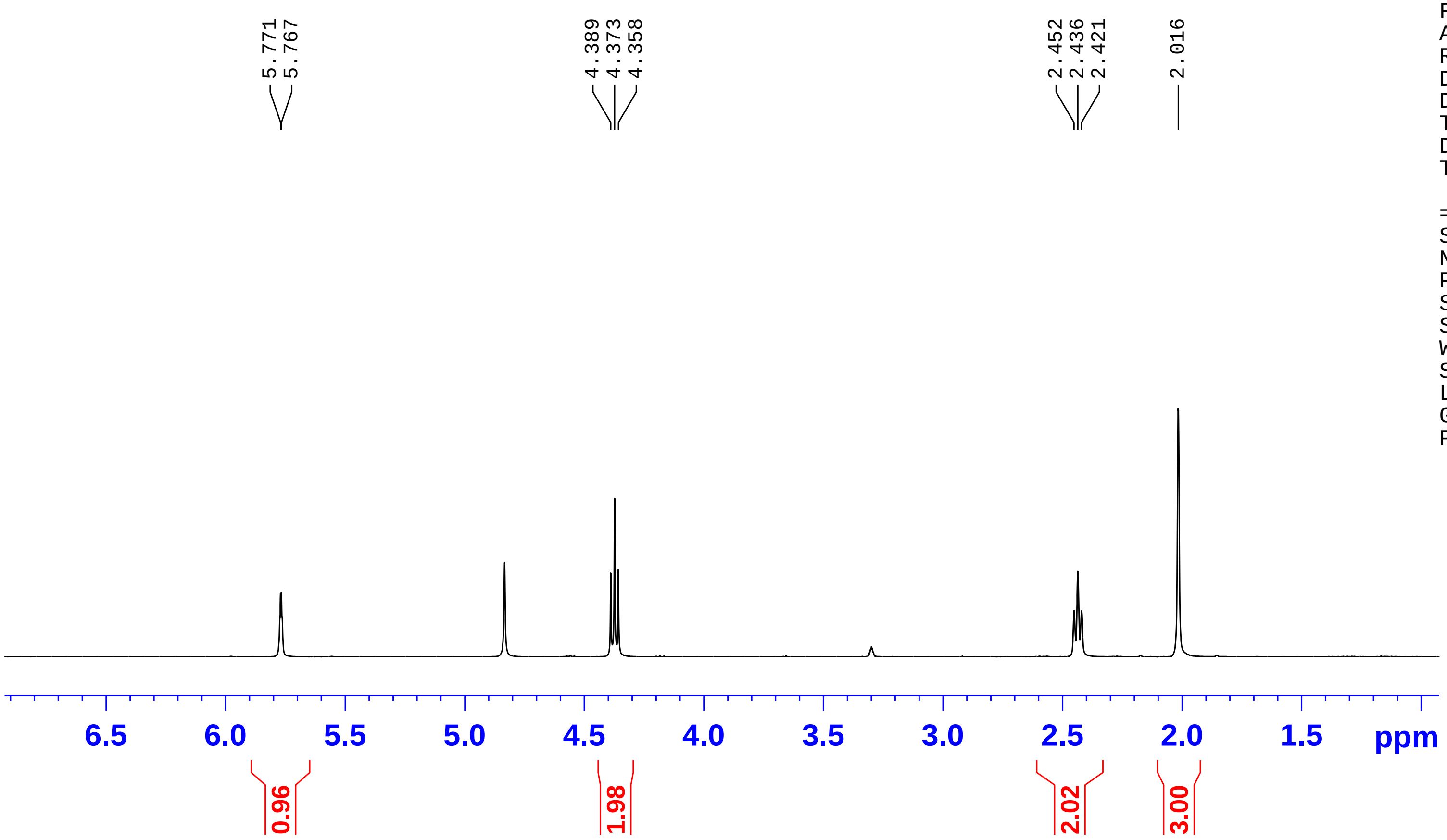

NAME	HB-C12 M 26mg
EXPNO	1
PROCNO	1
Date_	20200906
Time	13.43
INSTRUM	spect
PROBHD	5 mm PABBO BB-
PULPROG	zg30
TD	65536
SOLVENT	MeOD
NS	14
DS	2
SWH	8012.820 Hz
FIDRES	0.122266 Hz
AQ	4.0894966 sec
RG	101
DW	62.400 usec
DE	6.50 usec
TE	296.0 K
D1	1.00000000 sec
TD0	1

======== CHANNEL f1 ========

SFO1	400.1324710 MHz
NUC1	1H
P1	13.90 usec
SI	32768
SF	400.1300117 MHz
WDW	EM
SSB	0
LB	0.30 Hz
GB	0
PC	1.00

Figure S64. ^{1}H NMR spectrum of **25**.

HB-C12 M 26mg C

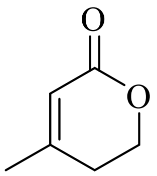

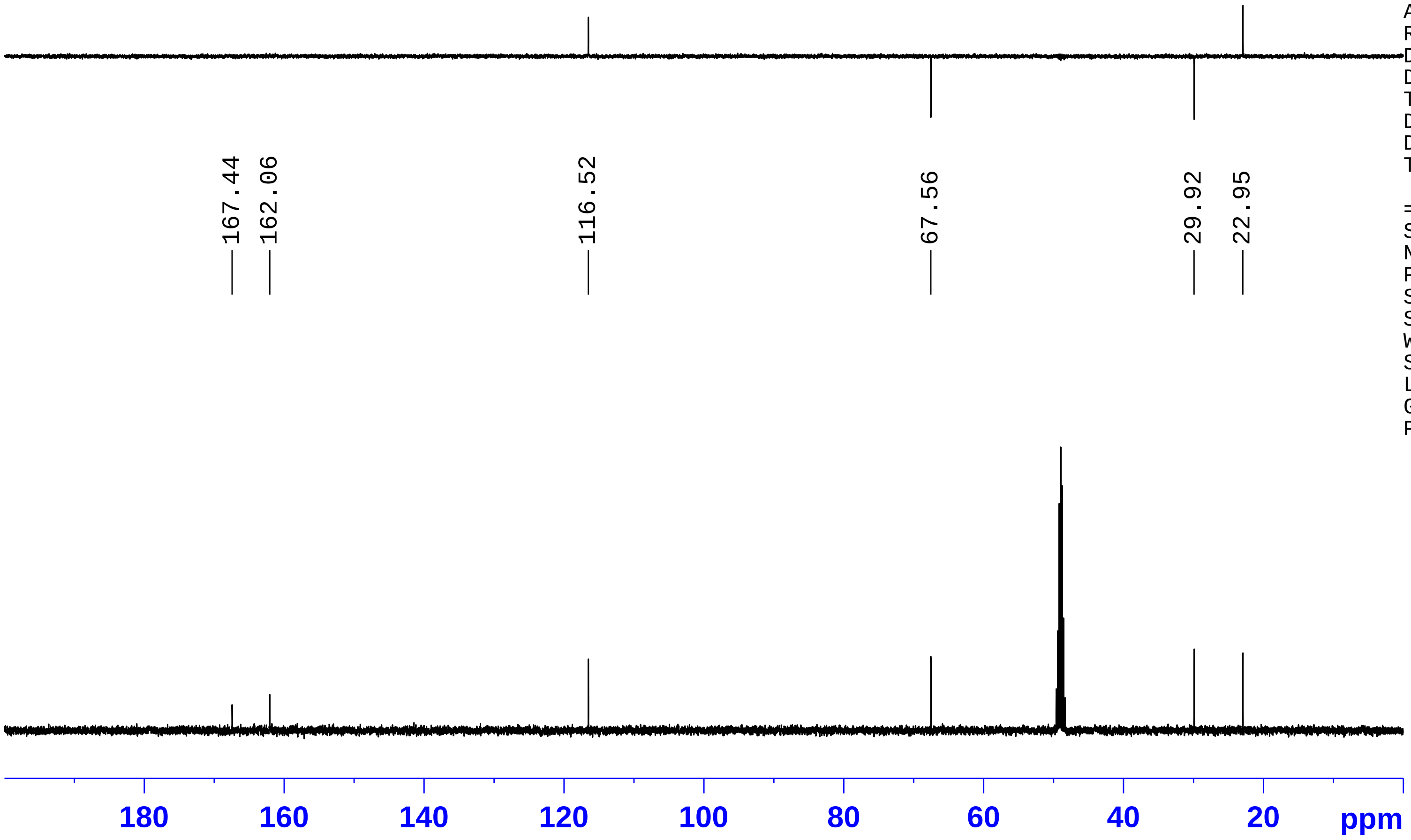

NAME	HB-C12 M 26mg
EXPNO	2
PROCNO	1
Date_	20200906
Time	13.45
INSTRUM	spect
PROBHD	5 mm PABBO BB-
PULPROG	zgpg30
TD	65536
SOLVENT	MeOD
NS	11
DS	4
SWH	24038.461 Hz
FIDRES	0.366798 Hz
AQ	1.3631988 sec
RG	203
DW	20.800 usec
DE	6.50 usec
TE	296.1 K
D1	2.00000000 sec
D11	0.03000000 sec
TD0	1

======== CHANNEL f1 ========

SFO1	100.6228293 MHz
NUC1	13C
P1	12.37 usec
SI	32768
SF	100.6126314 MHz
WDW	EM
SSB	0
LB	1.00 Hz
GB	0
PC	1.40

Figure S65. ^{13}C NMR spectrum of **25**.
